# Excited-state configuration controls the ability of nitroarenes to act as energy transfer catalysts

**DOI:** 10.1038/s41929-025-01453-z

**Published:** 2025-12-17

**Authors:** Martin Rihtaršič, Byeongseok Kweon, Piotr T. Błyszczyk, Alessandro Ruffoni, Enrique M. Arpa, Daniele Leonori

**Affiliations:** 1 Institute of Organic Chemistry, RWTH Aachen University, Aachen, Germany; 2 Institute for Organic Chemistry, University of Münster, Münster, Germany

**Keywords:** Photocatalysis, Photocatalysis

## Abstract

Energy transfer (EnT) catalysis enables the selective population of triplet excited states without previous singlet excitation, thus eliminating the need for high-energy irradiation. Traditionally, EnT catalysis has been approached by developing specific photosensitizers with triplet energies (*E*_T_) that match those of the targeted substrates. Here we introduce an alternative approach to EnT using widely available nitroarenes as photocatalysts. Our findings reveal that their catalytic efficiency is governed by the localization of their excited state rather than *E*_T_. Specifically, ^3^*π*,π* nitroarenes, where the excitation is centred on the aromatic core rather than the nitro group, exhibit superior catalytic performance compared with their ^3^*n*,π* counterparts. We have demonstrated the utility of this concept for nitroarene photocatalysis in contra-thermodynamic *E*-to-*Z* alkene isomerization and [2 + 2] cycloadditions. Additionally, we use the energetic descriptor Δ*E*_TT_ as easy tool to distinguish the preferential population of ^3^*n*,π* versus ^3^*π*,π* triplet states and therefore accelerate the identification of novel photosensitizers.

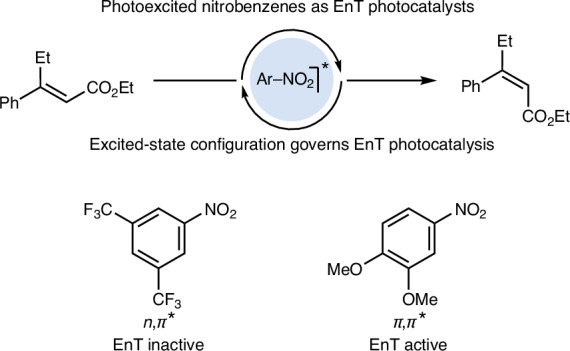

## Abstract (web summary)

Photoexcited nitroarenes are traditionally consumed as reactive intermediates in transformations. Now, it is shown that simple and cheap nitroarenes can be used as energy transfer photocatalysts in reactions such as *E*-to-*Z* alkene isomerization and [2 + 2] intramolecular cycloadditions.

## Main

**Fig. 1:**
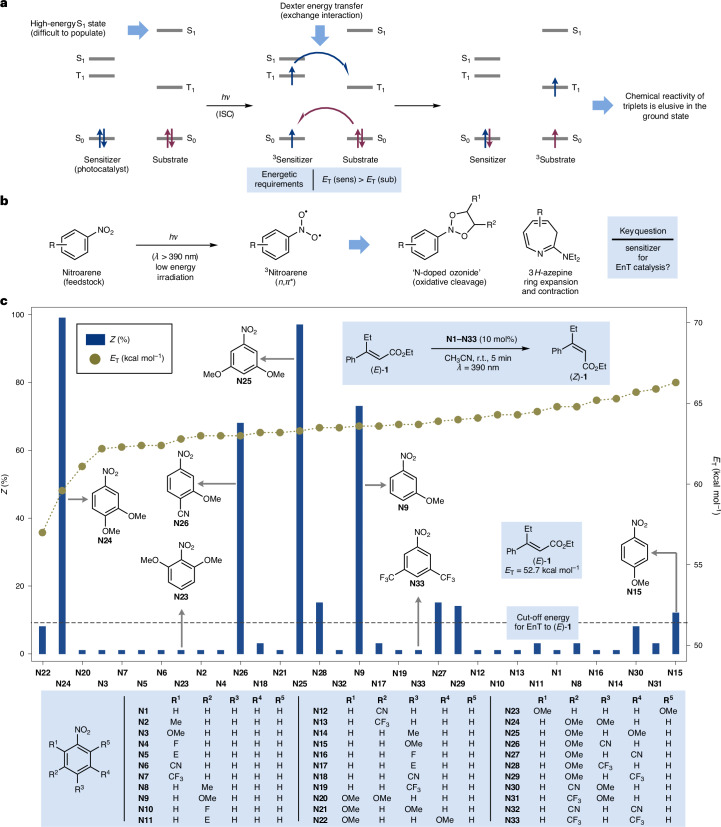
EnT catalysis using triplet nitroarenes. **a**, EnT photocatalysis exploits the ability of triplet sensitizers to transfer their energy to the substrate, providing it has a lower *E*_T_. **b**, Nitroarenes can be photoexcited to their triplet states using visible light. These species can be used in many processes but their ability to be employed as photocatalysts in EnT remains unexplored. **c**, *E*-to-*Z* photoisomerization efficiency of cinnamate (*E*)-**1** for all the tested nitroarenes sorted by increasing triplet energy (*E*_T_). The adiabatic energy for the lowest-lying triplet state of each nitroarene was calculated at the M06-2X/cc-pVTZ/SMD(MeCN)//M06-2X/cc-pVDZ level of theory. Et, ethyl; Me, methly; Ph, phenyl.

The population of electronically excited states enables reactivity patterns that are inaccessible in the ground state^1,2^. However, direct photoexcitation of organic molecules is often hindered by their low absorbance profiles which requires the use of high-energy irradiation (for example, UV-B). This requires the use of specialized equipment and can compromise compatibility with many functional groups^3,4^. Energy transfer (EnT) catalysis offers a powerful alternative by directly populating triplet excited states (T_1_), bypassing the higher-energy singlet states (S_1_) and using irradiation wavelengths outside the substrate’s ground-state absorbance profile^3,5–7^. In EnT catalysis, a photocatalyst functions as a light-harvesting system, transferring energy to the substrate through a Dexter energy transfer mechanism^8^ (Fig. 1a). Effective photosensitizers are defined by several key properties: an absorption profile suitable for low-energy irradiation (preferably visible light), strong spectral overlap with the substrate’s absorption spectrum, high intersystem crossing (ISC) quantum yield, and a long-lived triplet state to facilitate bimolecular quenching^5^. Importantly, the triplet energy (*E*_T_) of the photocatalyst must exceed that of the substrate to drive an exergonic EnT process.

Recent developments in EnT catalysis have focused on designing both transition metal and organic photosensitizers where fine-tuning of their structural features modulates their *E*_T_ and expands their synthetic utility^9–14^. In this study, we introduce an alternative approach to EnT catalysis in which the sensitization process is governed by the nature of the excited-state configuration. By switching the localization of the excited state within the photosensitizer we demonstrate the effective use of nitroarenes—some of the simplest and most abundant feedstocks—as versatile EnT photocatalysts (Fig. 1b). This strategy establishes the use of nitroarenes as catalysts and provides a practical alternative to systems reliant on precious metals or synthetically demanding scaffolds.

## Results

### *E*-to-*Z* isomerization of cinnamates

Our group and others have recently demonstrated the ability of triplet nitroarenes to participate in various synthetically valuable transformations, such as the oxidative cleavage of alkenes, C(*sp*^3^)–H oxidation, and aromatic ring expansion and contraction reactions^15–22^ (Fig. 1b). Upon photoexcitation, nitroarenes populate the T_1_ state, characterized by a distinct ^3^*n*,π* configuration, localized on the NO_2_ group causing loss of its planarity. This excited-state geometry causes the nitro group to behave as a double oxygen radical species that can engage in radical-type cascade reactions with alkenes^15–17^, activated C(*sp*^3^)–H bonds^18,19^ and P(III) reagents^20–22^. So far, triplet nitroarenes have been used in stoichiometric reactivity modes resulting in their conversion into other chemical species. We recently became interested in understanding if their triplet character could also be exploited in catalytic applications for EnT^23–25^. Given the wide commercial availability of nitroarenes, we were intrigued by the potential to tailor their reactivity by rational modification of their substitution patterns. Moreover, nitroarenes possess favourable properties for such applications, including strong absorption in the UV-A/visible range and highly efficient ISC (*Φ* ≈ 0.8)^26^.

We initiated this study by investigating the contra-thermodynamic *E*-to-*Z* isomerization of cinnamate (*E*)-**1**, a process for which the current state-of-the-art utilizes several types of photocatalysts, generally over extended reaction times (∼24 h)^27–31^. Leveraging the broad availability of nitroarenes, we rapidly screened a wide range of mono- and disubstituted systems (**N1**–**N33**, 10 mol%) (Fig. 1c). Pleasingly, we found that the desired isomerization could be achieved with several reagents under irradiation with purple light-emitting diodes (LEDs, *λ* = 390 nm) at room temperature in CH_3_CN solvent. Pleasingly, the reaction times were reduced to just a few minutes, demonstrating the high catalytic efficiency of some specific triplet nitroarenes.

A particularly intriguing finding was the dramatic variation in catalytic performance among different nitroarenes based on their substitution patterns. For example, 3,5-(CF_3_)_2_-nitroarene **N33**, which is highly effective in the oxidative cleavage of alkenes^15^, showed no capacity for isomerization. In contrast, substrates bearing electron-donating OMe groups—typically unreactive in this transformation—exhibited good-to-excellent reactivity. Specifically, *p*-substituted **N15** achieved 12% *Z* isomerization, while the *m*-substituted **N9** increased this to 73%. Derivatives featuring two OMe groups performed best, with *m*,*m*-functionalized **N25** yielding 97% *Z* isomerization and the *m*,*p* derivative **N24** emerging as the optimal catalyst, achieving a *Z*:*E* ratio of 99:1. All reactions tested resulted in quantitative mass balances.

### Investigating differences in EnT efficiency

To understand the varying EnT effectiveness of these nitroarenes, we recorded their UV/visible absorption spectra, confirming they could all be photoexcited with purple LEDs (Supplementary Fig. 4). We then used computational chemistry to calculate their triplet energies. This study revealed that all nitroarenes have similar *E*_T_ values, in the range of *E*_T_ = 57–67 kcal mol^−1^, which makes them energetically similar to the widely used [Ir(dF(CF_3_)ppy)_2_(dtbbpy)(PF_6_)] (*E*_T_ = 61.8 kcal mol^−1^)^32^ and in most cases superior to many commonly used organic dyes such as 4CzIPN (*E*_T_ = 58.3 kcal mol^−1^)^33^. Interestingly, although the *E*_T_ of all nitroarenes is higher than that of (*E*)-**1** (*E*_T_ = 52.5 kcal mol^−1^), implying favourable energetic profiles, stark differences in catalytic reactivity were observed. This unexpected outcome challenges the current understanding of EnT catalysis, which largely emphasizes the energetic alignment of excited states as the primary determinant for sensitization.

**Fig. 2:**
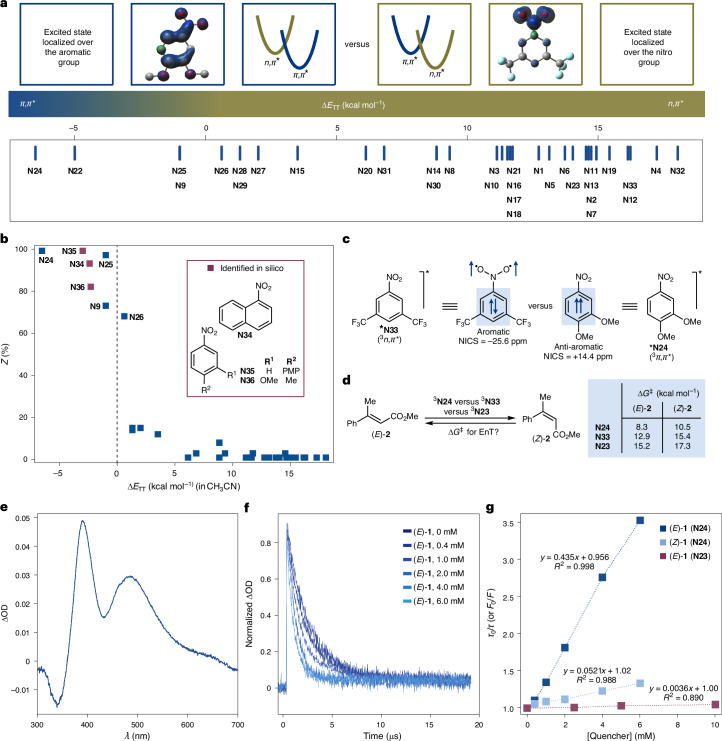
Mechanistic studies for triplet nitroarene-catalysed *E*-to-*Z* isomerizations. **a**, Top: spin densities of ^3^*π*,π* (**N24**) and ^3^*n*,π* (**N33**); bottom: scale of Δ*E*_TT_ values of **N1**–**N33**. Red, oxygen; grey, carbon; blue, nitrogen; cyan, fluorine; hydrogens omitted for clarity. **b**, Contra-thermodynamic *E*-to-*Z* isomerization of (*E*)-**1** with **N1**–**N33** plotted against their Δ*E*_TT_ values. **c**, Calculated nucleus-independent chemical shifts, NICSzz(1), values for selected ^3^*π*,π* (**N24**) and ^3^*n*,π* (**N33**) nitroarenes at the M06-2X/cc-pVTZ/SMD(MeCN)//M06-2X/cc-pVDZ level of theory. **d**, Activation barriers for EnT using Marcus theory calculated at the M06-2X/cc-pVTZ/SMD(CH_3_CN)//M06-2X/cc-pVDZ level of theory. **e**, Transient absorption spectrum for **N24** in deaerated CH_2_Cl_2_ (*λ*_exc_ = 355 nm). **f**, Normalized transient decay traces for **N24** in deaerated CH_2_Cl_2_ (*λ*_exc_ = 355 nm) monitored at 390 nm upon addition of increasing amounts of (*E*)-**1**. In all cases, the decays were fitted to a monoexponential function. **g**, Stern–Volmer plots for quenching of photoexcited **N24** by (*E*)-**1**, **N24** by (*Z*)-**1**, and **N23** by (*E*)-**1**. PMP, *para*-methoxyphenyl.

To better understand the photochemical behaviour of nitroarenes, we conducted additional computational investigations aimed at characterizing their excited-state geometries (Supplementary Table 1). This analysis revealed that the excited-state configurations of these species changed on the basis of their substitution patterns. Specifically, photoexcited nitroarenes can populate either ^3^*n*,π* or ^3^*π*,π* triplet states, differing in the localization of the unpaired electrons: the nitro group for ^3^*n*,π* and the aromatic ring for ^3^*π*,π* (ref. ^34^; Fig. 2a). To quantify the energetic preference between these states, we used the descriptor Δ*E*_TT_ = *E*_T_(^3^*π*,π*) – *E*_T_(^3^*n*,π*) and used this energetic parameter to construct an excited-state configuration scale. According to this scale, nitroarenes with Δ*E*_TT_ > 0 preferentially populate the ^3^*n*,π* state, and those with Δ*E*_TT_ < 0 preferentially populate the ^3^*π*,π* state. Plotting reaction efficiency against Δ*E*_TT_ revealed two distinct reactivity clusters: the ^3^*π*,π* nitroarenes were associated with high *E*-to-*Z* isomerization, while the ^3^*n*,π* exhibited low or no reactivity (Fig. 2b).

We wondered whether we could leverage this classification to identify new nitroarenes active in EnT photocatalysis. An in silico screen of several other nitroarenes allowed us to identify three additional ^3^*π*,π* species not included in our initial screening (**N34**, Δ*E*_TT_ = –2.4 kcal mol^−1^; **N35**, –3.0 kcal mol^−1^; and **N36**, –2.3 kcal mol^−1^). These nitroarenes exhibited high *E*-to-*Z* isomerization efficiency (82–99%), validating excited-state configuration as a key predictor EnT reactivity. It should be noted that although **N34** and **N35** are structurally quite different from the nitroarenes studied initially, they were nonetheless classified correctly based on the value of Δ*E*_TT_, demonstrating the strength of this index in predicting photosensitizing capabilities.

The stark differences in EnT reactivity between ^3^*π*,π* and ^3^*n*,π* nitroarenes are not fully understood but appear to stem from kinetic factors influencing the sensitization process. One notable difference between these two species is their excited-state lifetimes: ^3^*π*,π* nitroarenes have notably longer lifetimes (*τ* ≈ μs) than ^3^*n*,π* nitroarenes (*τ* ≈ ps)^26^, which might favour applications in bimolecular processes. However, extending reaction times for ^3^*n*,π* nitroarenes (for example, **N33**, up to 6 h) in EnT reactions led to marginal improvements in their isomerization capacity. This behaviour contrasts with their performance in alkene ozonolysis, where longer reaction times lead to higher yields^15^.

Furthermore, ^3^*n*,π* and ^3^*π*,π* nitroarenes also exhibit structural differences: ^3^*n*,π* nitroarenes have a pyramidalized nitrogen atom, whereas ^3^*π*,π* nitroarenes adopt a planar geometry. These structural variations can influence sensitization reactivity by affecting the energy required for geometric reorganization. For EnT to occur, ^3^*n*,π* nitroarenes must first revert to planarity, whereas ^3^*π*,π* nitroarenes do not undergo such rearrangement. Consequently, the reorganization energy and activation barrier for EnT are expected to be higher for ^3^*n*,π* nitroarenes than for ^3^*π*,π* nitroarenes.

Another key factor that could influence the contrast in observed reactivity is related to the differences in excited-state aromaticity of the ^3^*n*,π* and ^3^*π*,π* states. Triplet ^3^*n*,π* states, exclusively localized over the nitro group, leave the electron density of the phenyl ring nearly unperturbed with respect to the ground state, thus rendering the molecule Hückel aromatic^35^. In complete contrast, the highly delocalized nature of ^3^*π*,π* states makes them Baird antiaromatic^36^. Therefore, the relief of the excited-state antiaromaticity of ^3^*π*,π* states might be favouring their reactivity in EnT reactions, a process that cannot take place for ^3^*n*,π* states^37,38^.

Although we cannot ascertain which of the factors discussed above (excited-state lifetime, structural reorganization, (anti)aromaticity, triplet energy) contributes the most in lowering the activation energies for EnT reactions, we decided to calculate these barriers to corroborate whether our computational methodology can correctly predict the outcome of such reactions. Using the procedure developed by Maseras and co-workers^39^, which extends Marcus theory to EnT, we calculated the activation energies for the *E*-to-*Z* and *Z*-to-*E* photoisomerization of cinnamate *E*-**2** using **N24** and **N33** as models for ^3^*π*,π* and ^3^*n*,π* nitroarenes, respectively (Supplementary Table 3). We found that the *E*-to-*Z* isomerization with **N24** has the lowest barrier (Fig. 2d, 8.3 kcal mol^−1^). All the other calculated barriers are at least 2 kcal mol^−1^ higher, which proves that the most facile process is the contra-thermodynamic *E*-to-*Z* isomerization in the presence of ^3^*π*,π* nitroarenes.

To further corroborate our understanding of the sensitization process, we performed nanosecond transient absorption spectroscopy studies using laser flash photolysis to monitor the behaviour of several nitroarenes in the presence of (*E*)-**1** and (*Z*)-**1**. The triplet states of these species could be observed using the excitation wavelength *λ* = 355 nm and their decay measured through time-resolved experiments. For instance, **N24** produced a transient with an absorption maximum at *λ*_max_ = 390 nm and a lifetime of *τ* = 2.8 µs (Fig. 1e). Stern–Volmer quenching studies in the presence of (*E*)-**1** and (*Z*)-**1** revealed differing bimolecular quenching constants: *K*_q_ (*E*)-**1** = 1.67 × 10^8 ^M^−1^ s^−1^ and *K*_q_ (*Z*)-**1** = 0.22 × 10^8 ^M^−1^ s^−1^, which account for the observed EnT isomerization reactivity (Fig. 1f,g). Most ^3^*n*,π* nitroarenes exhibit shorter lifetimes, making them undetectable via laser flash photolysis in the nanosecond time domain. One exception is **N23** which has a long-lived ^3^*n*,π* transient (*λ*_max_ = 280 nm and *τ* = 310 ns). Although the lifetime is sufficiently long for application in triplet sensitization, it was not active in *Z* isomerization under our photocatalytic conditions. Quenching studies of **N23** with (*E*)-**1** yielded a bimolecular quenching constant of 1.2 × 10^7 ^M^−1^ s^−1^, more than an order of magnitude lower than **N24** (Fig. 1g). This is in line with the calculated activation barrier for energy transfer to (*E*)-**2**, which is 6.9 kcal mol^−1^ higher for ^3^**N23** compared with ^3^**N24** (Fig. 2d). This further underscores the limited capability of ^3^*n*,π* nitroarenes to serve as EnT catalysts despite their potentially matching *E*_T_ and also lifetime.

### Scope of *E*-to-*Z* isomerization

**Fig. 3:**
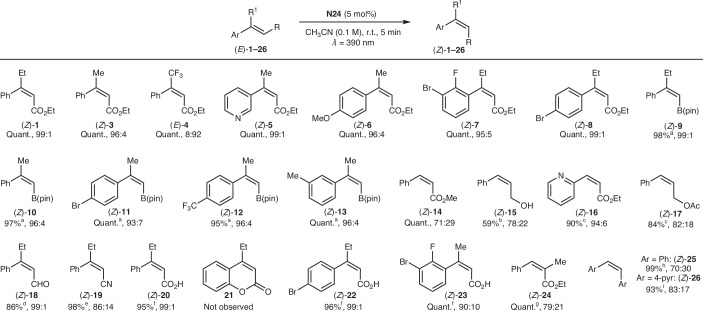
Scope for the triplet nitroarene-catalysed *E*-to-*Z* isomerizations. ^a^**N24** (10 mol%), CH_3_CN (0.03 M), 1-h reaction time. ^b^**N34** (10 mol%), CH_3_CN (0.05 M), 60-h reaction time. ^c^**N24** (10 mol%), CH_3_CN (0.05 M), 6-h reaction time. ^d^**N24** (10 mol%). ^e^**N24** (10 mol%), 10-min reaction time. ^f^**N24** (10 mol%), CH_3_CN (0.05 M), 30-min reaction time. ^g^**N35** (10 mol%), 1-h reaction time. ^h^**N24** (20 mol%), 10-min reaction time. ^i^**N24** (20 mol%), 16-h reaction time. Ar, aryl; Ac, acetyl; pin, pinacolato; pyr, pyridine; quant., quantitative.

We then evaluated the generality of the contra-thermodynamic isomerization process using **N24** as the EnT catalyst (Fig. 3). Pleasingly, we successfully engaged a variety of differentially functionalized cinnamate derivatives, with substitutions at both the alkene and aromatic units (**1**, **3**–**7**). In all cases, the processes were quantitative, resulting in high *E*-to-*Z* isomerization efficiency. The aromatic unit could be changed with a pyridine ring (**5**) and with a poly functionalized benzenoid system featuring bromine atoms for further cross-coupling applications (**7**, **8**). Furthermore, we extended the reactivity to *E*-styrenyl pinacol boronic esters (**9**–**13**). The high isomerization observed for the boronates offers a convenient alternative to current methods that use Ir(ppy)_3_ as the EnT photocatalyst and typically require extended reaction times (for example, 24 h)^40,41^. The EnT could also be applied to derivatives lacking an α-substituted styrenyl unit as demonstrated by the use of acrylate (**14**), cinnamyl alcohol (**15**), pyridyl acrylate (**16**) and cinnamyl acetate (**17**). Notably, for compound **15**, higher isomerization was obtained using **N34**.

This catalytic system could also be successfully applied to the *E/Z* isomerization of other cinnamyl derivatives bearing aldehyde (**18**), nitrile (**19**) and carboxylic acid (**20**, **22**, **23**) functionalities. In the case of cinnamic acids, the fact that triplet nitroarenes can undergo EnT but not redox chemistry prevents concomitant coumarin formation (for example, **21** from (*Z*)-**20**)^42^. This results in a different outcome compared with standard EnT photocatalysts that also operate under photoredox conditions. This same characteristic enables compatibility with redox-active functionalities, such as electron-deficient aryl bromides, which could otherwise undergo single-electron transfer reduction^43^.

Furthermore, we evaluated the reactivity of β-methylcinnamate (**24**) and stilbenes (**25**, **26**). Derivatives such as **24** are typically challenging in this transformation due to the absence of the critical 1,3-allylic strain between the *ortho* proton of the aryl ring and the α-substituent of the alkene^5,27^. Stilbenes provide lower *E/Z* ratios because both the *E* and *Z* isomers have accessible triplet energies for sensitization^6^. Nevertheless, moderate to good levels of isomerization were observed.

As for limitations, we were unable to engage alkyl-substituted acrylates or dialkyl olefins because they feature high triplet energies with negligible difference between the two isomers. This effectively precludes selective accumulation of one isomer over the other^5^ (Supplementary Note 13).

### Application to other photocatalytic EnT processes

**Fig. 4:**
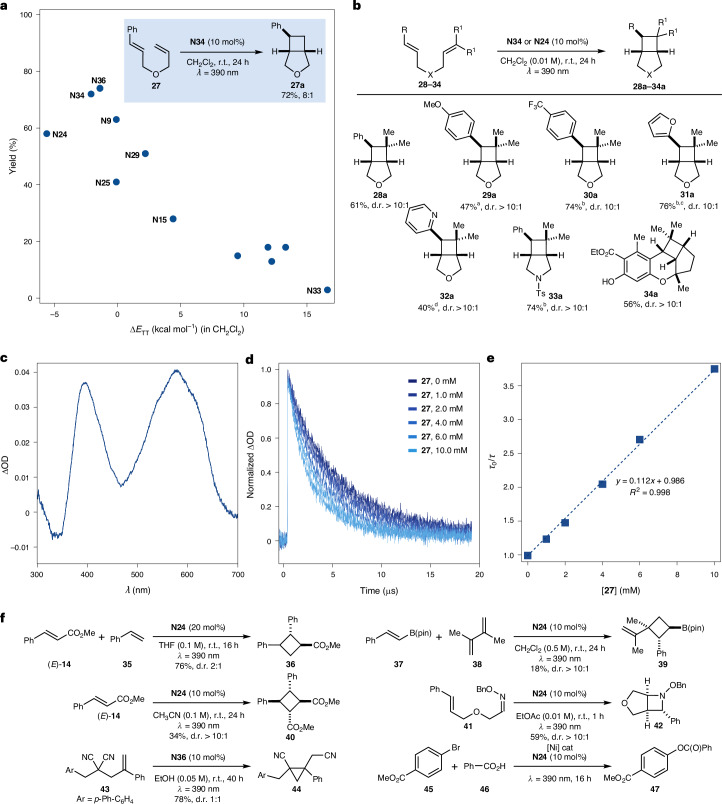
Nitroarene photocatalysis can be used in other EnT processes. **a**, Plot of yield versus Δ*E*_TT_ for the intramolecular [2 + 2] cycloaddition of **27**. **b**, Reaction scope for [2 + 2] cycloaddition. **c**, Transient absorption spectrum for **N34** in deaerated CH_2_Cl_2_ (*λ*_exc_ = 355 nm). **d**, Normalized transient decay traces for **N34** in deaerated CH_2_Cl_2_ (*λ*_exc_ = 355 nm) monitored at 566 nm upon addition of increasing amounts of **27**. In all cases, the decays were fitted to a monoexponential function. **e**, Corresponding Stern–Volmer plot. **f**, Nitroarene photocatalysis applied to other cycloaddition reactions: ^a^**N24** (5 mol%); ^b^**N24** (10 mol%); ^c^6-h reaction time; ^d^**N24** (15 mol%), 48-h reaction time. Bn, benzyl; THF, tetrahydrofuran; Ts, tosyl.

To establish the generality of ^3^*π*,π* nitroarenes as EnT catalysts, we evaluated their performance in other synthetically relevant transformations^44^. One such example is the photochemical intramolecular [2 + 2] cycloaddition of styrene **27**, which features a pendant olefin (Fig. 4a). Current state-of-the-art methods for this reaction use [Ir(dF(CF₃)ppy)_2_(dtbbpy)(PF_6_)] or tailor-made flavin as the sensitizer, under blue light irradiation (*λ* = 402 nm)^45,46^.

Pleasingly, our screening of nitroarenes across a range of Δ*E*_TT_ values revealed a similar situation whereby reactivity is independent of the matching *E*_T_ of the various nitroarenes. This provided the same reactivity trend where ^3^*π*,π* derivatives consistently demonstrated high reactivity, while ^3^*n*,π* nitroarenes exhibited considerably lower reaction efficiency. From this screening, nitroarene **N34** emerged as the optimal catalyst and was used for further mechanistic studies. As illustrated in Fig. 4b, nitroarene photocatalysis enabled the rapid assembly of a series of bicyclic derivatives (**27a**–**33a**). These products spanned a wide range of substitution patterns, including the formation of quaternary centres (**28a**–**34a**), incorporation of both electron-rich furan (**31a**) and electron-poor pyridine (**32a**) residues, and the assembly of structurally complex tetracyclic derivative (**34a**), an intermediate to (±)-cannabiorcicyclolic acid^45^. It is important to note that in some cases, **N24** provided slightly superior reaction efficiency, further demonstrating the benefits of using broadly available feedstocks as sensitizers.

In terms of mechanistic understanding, transient absorption spectroscopy, performed using flash laser photolysis with excitation centred at *λ* = 355 nm, enabled the observation of the **N34** triplet state, which featured a lifetime of *τ* = 4.0 μs (Fig. 4c). Stern–Volmer quenching studies further revealed a bimolecular quenching constant of 2.3 × 10^7^ M^−1^ s^−1^ with **27**, confirming its effectiveness as a triplet sensitizer (Fig. 4d,e).

To further demonstrate the potential of ^3^*π*,π* nitroarenes as photocatalysts, we explored their reactivity in a broader range of EnT transformations. These included intermolecular [2 + 2] cycloadditions involving cinnamate (*E*-**14**) and styrenyl boronate (**37**) as energy acceptors, combined with styrene (**35**) and dimethylbutadiene (**38**) as olefin partners, respectively^47,48^. The resulting cyclobutanes (**36**, **39**) were obtained in good to moderate yields, with high diastereoselectivity. Additionally, triplet excited nitroarenes effectively catalysed the cyclodimerization of cinnamate (*E*-**14**) to afford a tetrasubstituted cyclobutane (**40**) in moderate yield and with excellent stereocontrol^49^. We also applied these photocatalysts in an aza Paternò–Büchi reaction using oxime **41** to deliver the bicyclic azetidine **42** (ref. ^50^), and in a radical translocation process involving substrate **43** to furnish the corresponding cyclopropane derivative **44** (ref. ^51^). Collectively, these results highlight the broad synthetic utility of ^3^*π*,π* nitroarenes in diverse EnT-mediated transformations, reinforcing their potential as versatile and sustainable photocatalysts. In terms of current limitations, we could not extend this reactivity to the sensitization of organometallic complexes, as demonstrated in a dual-catalytic approach for the coupling of aryl bromides and carboxylic acids, where success was not achieved^52^.

## Conclusions

The field of energy transfer photocatalysis has traditionally focused on designing sensitizers with triplet energies (*E*_T_) closely matching those of the target substrates. Our findings reveal that triplet nitroarenes can act as powerful sensitizers for EnT applications. Crucially, despite having similar *E*_T_ values, these species exhibit drastically different reactivity profiles on the basis of their excited-state geometry. This means that excited-state localization can be a crucial factor to consider when approaching the development of novel sensitizers. We demonstrated that nitroarenes with ^3^*π*,π* configurations display superior catalytic efficiency than their ^3^*n*,π* counterparts. This configuration-dependent reactivity highlights the pivotal role of the excited-state electronic configuration, which influences key factors such as lifetimes, transition-state barriers, reorganization barriers and (anti)aromaticity.

Moreover, this work emphasizes the practical and sustainable advantages of using readily available and structurally diverse nitroarenes as photocatalysts. These feedstocks provide an economical and environmentally friendly alternative to traditional sensitizers that rely on precious metals or synthetically complex scaffolds. We hope that this approach will serve as a foundation for the broader identification of feedstock materials, leveraging their excited-state configurations to advance the pursuit of sustainable and efficient photocatalysis.

## Methods

### General procedure for the *E*-to-*Z* isomerization of alkenes

An oven-dried microwave vial containing a stir-bar was charged with the *E* isomer (1.0 equiv.) and **N24** (5 mol%). The vial was capped with a Supelco aluminium crimp seal with septum (PTFE/butyl) and was evacuated and refilled with argon (×3). Dry and degassed CH_3_CN (0.1 M) was added, and the reaction mixture was stirred (>500 r.p.m.) under irradiation with a 390-nm Kessil LED lamp (5-cm distance, 100% intensity, fan on) for the specified time. Quantitative ^1^H NMR spectroscopy was used to obtain the *E*/*Z* ratio. The solvent was removed under reduced pressure and the residue purified by flash column chromatography to give the pure product.

### General procedure for the intramolecular [2 + 2] cycloaddition

An oven-dried microwave vial containing a stir-bar was charged with 1,6-diene (1.0 equiv.) and **N24** or **N34** (10 mol%). The vial was capped with a Supelco aluminium crimp seal with septum (PTFE/butyl) and was evacuated and refilled with argon (×3). Dry and degassed CH_2_Cl_2_ (0.01 M) was added, and the reaction mixture was stirred (>500 r.p.m.) under irradiation with a 390-nm Kessil LED lamp (5-cm distance, 100% intensity, fan on) for 24 h. Quantitative ^1^H NMR spectroscopy was used to obtain the ratio of diastereomers. The solvent was removed under reduced pressure and the residue purified by flash column chromatography to give the pure product.

## Supplementary information

Supplementary Information Supplementary Figs. 1–21, Tables 1–8 and Notes 1–14.

Supplementary Computational Data 1 The atomic coordinates of optimized structures.

## Data Availability

The authors declare that the data supporting the findings of this study are available within the paper and its Supplementary Information files. Should any raw data files be needed in another format they are available from the corresponding authors upon reasonable request.
