# Supplementary material for: Excited-state configuration controls the ability of nitroarenes to act as energy transfer catalysts
Source: Nat Catal. 2025 Dec 17;8(12):1361–9. doi: 10.1038/s41929-025-01453-z (PMC12727535; doi:10.1038/s41929-025-01453-z)
Supplement: Supplementary file 1 — Supplementary Figs. 1–21, Tables 1–8 and Notes 1–14. [file 41929_2025_1453_MOESM1_ESM.pdf]

nature catalysis

Supplementary information https://doi.org/10.1038/s41929-025-01453-z

Excited-state configuration controls the ability of nitroarenes to act as energy transfer catalysts

In the format provided by the authors and unedited

Table of Contents

Supplementary Methods 2

Supplementary Note 1: General Information 2

Supplementary Note 2: General Procedures 4

Supplementary Note 3: Preparation of Starting Materials 7

Supplementary Note 4: Emission Spectra of Kessil LED lamps 19

Supplementary Note 5: Pictures of Reaction Set-up 20

Supplementary Note 6: List of Nitroarenes Used in the Study 21

Supplementary Note 7: Characterisation of Nitroarene Triplet Energies & NICS Values 22

Supplementary Note 8: Isomerization of α,β-Unsaturated Esters 24

Supplementary Note 9: Mechanistic Investigations 27

Absorption Profiles of N1-36 27

Detection of Ground State EDA Complexes 34

Calculation of the Activation Barriers for Energy Transfer 36

Laser Flash Photolysis and Stern-Volmer experiments 38

Computational Absorption Spectra of ^{3}N23 48

Supplementary Note 10: Intramolecular [2+2] Cycloaddition 49

Supplementary Note 11: Optimization of other EnT transformations 53

Intermolecular [2+2] cycloaddition 53

Aza-Paternò–Büchi 55

Translocation 56

Supplementary Note 12: Substrate Scope 57

Supplementary Note 13: EnT Reactivities of Unactivated Substrates 68

Supplementary Note 14: NMR Spectra 69

Supplementary References 84

Supplementary Methods

Supplementary Note 1: General Information

Experimental

All fine chemicals were used directly without purification unless otherwise stated. All air- and moisture-sensitive reactions were carried out under an Ar or N_2 atmosphere using standard Schlenk manifold techniques. All solvents were purchased from Acros as 99.8% purity or purified using MBraun SPS-800 purification system. Solvents were degassed by Ar or N_2 bubbling prior to using in photochemical reactions unless otherwise specified. ^{1}H, ^{13}C and ^{19}F Nuclear Magnetic Resonance (NMR) spectra were recorded on a Bruker Avance Neo 600 MHz, Bruker Avance 400 MHz, Varian VNMRS 600 MHz or Varian VNMRS 400 MHz. ^{1}H and ^{13}C Nuclear Magnetic Resonance (NMR) spectra were acquired at various field strengths as indicated and were referenced to $CHCl_3$ (7.27 and 77.16 ppm for ^{1}H and ^{13}C, respectively) or DMSO-d_6 (2.50 and 39.5 ppm for ^{1}H and ^{13}C, respectively), or by the instrument internally after locking and shimming to the deuterated solvent (for ^{19}F). ^{1}H NMR coupling constants (J) are reported in Hertz (Hz) and refer to apparent multiplicities and not true coupling constants. Data is reported as follows: chemical shift (δ), integration, multiplicity (s = singlet, br s = broad singlet, d = doublet, t = triplet, q = quartet, quint. = quintet, sx = sextet, sp = septet, m = multiplet, dd = doublet of doublets, etc.). Isomeric ratios were determined by quantitative ^{1}H NMR (delay = 10 s) conducted on crude reaction mixtures and do not refer to the ratios following purification. High-resolution mass spectra were obtained using a Thermo Scientific LTQ Orbitrap XL spectrometer or a Finnigan MAT 95. Spectra were obtained using electron impact ionization (EI) or positive electrospray (ESI) techniques. Analytical TLC: aluminum backed plates pre-coated (0.25 mm) with Merck Silica Gel 60 F254. Compounds were visualized by exposure to UV-light or by dipping the plates in cerium ammonium molybdate (CAM) or potassium permanganate ($KMnO_4$) stain solutions, followed by heating. Flash column chromatography was performed using Merck Silica Gel 60 (40–63 μm). All mixed solvent eluents are reported as v/v solutions. Steady-state absorption spectra were obtained using a Shimadzu (UV-2600) UV-vis spectrophotometer with glass or quartz cuvettes. Duetta Fluorescence and Absorbance Spectrometer by Horiba was used for steady-state photoluminescence studies. The LP980KS setup from Edinburgh Instruments equipped with an Nd:YAG laser from Litron (Nano LG 300-10) was employed for transient absorption (TA) spectroscopy. The frequency tripled or quadrupled output with a wavelength of 355 nm or 266 nm served as the excitation source. The laser pulse duration was ~5 ns. The typical pulse energy used for transient absorption studies was 15 mJ. Detection of transient absorption spectra occurred on an iCCD camera from Andor. Kinetic traces at selected wavelengths were recorded using a photomultiplier tube. The spectroscopic experiments were performed at 293 K using a cuvette holder that allows temperature control. TAS samples were bubbled with N_2 before acquisition. For quenching experiments, the appropriate volumes of a freshly prepared organic quencher substrates were added to the purged nitroarene solution. All the photophysical measurements were performed in dichloromethane solution at room temperature in a quartz cuvette of 1.0 cm optical path length from Hellma Analytics**.** All the reactions were conducted in CEM 9 mL glass microwave tubes. The LEDs used are Kessil PR 160 390 nm.

Computational

All calculations have been carried out in the framework of density functional theory (DFT) using *Gaussian 16* software.[1] Specifically, the M06-2X exchange-correlation functional,[2] in combination with the cc-pVDZ basis set in gas phase,[3] was employed for geometry optimizations and the calculation of harmonic vibrational frequencies. All the optimized structures presented in this work were identified as potential-energy minima as they show all-real vibrational frequencies. More accurate single-point electronic energies and nucleus-independent chemical shifts (NICS) were computed using the same functional but with the larger cc-pVTZ basis set and including solvent effects (CH_3CN) with the SMD model.[4] For the NICS values,[5] we located the probe atoms 1 Å above the geometric center of the ring and following the perpendicular direction and we considered exclusively the zz component of the magnetic shielding tensor to minimize the effect of σ electrons, hence denoted $NICS_{zz}(1)$.

Supplementary Note 2: General Procedures

General Procedure for the synthesis of α,β-unsaturated esters – GP1

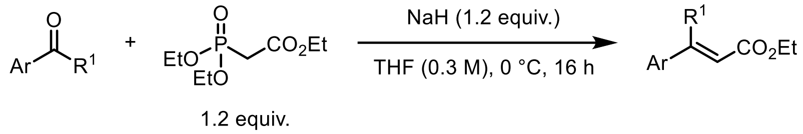

A suspension of NaH (192 mg, 4.8 mmol, 1.2 equiv., 60% in mineral oil) in dry THF (0.3 M, 13 mL) under Ar at 0 °C was treated dropwise with ethyl 2-(diethoxyphosphoryl)acetate (1.08 g, 952 μL, 4.8 mmol, 1.2 equiv.) and the mixture stirred for 1 h at 0 °C before the addition of ketone (4.0 mmol, 1.0 equiv.). The reaction was stirred for 1 h at 0 °C before being warmed to r.t. and stirred for an additional 24 h. H_2O (20 mL) and Et_2O (20 mL) were added, phases separated, and the organics were extracted with Et_2O (3 x 10 mL). The combined organic layers were dried ($MgSO_4$), filtered and evaporated. The crude residue was purified by flash column chromatography to provide the pure product.

General Procedure for the synthesis of α-substituted styrene boronic acid pinacol esters – GP2

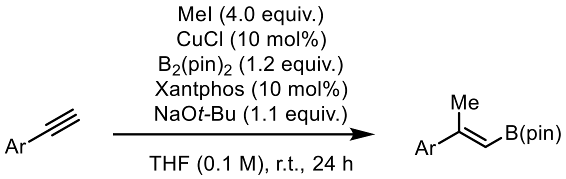

A mixture of CuCl (40 mg, 0.4 mmol, 10 mol%), XantPhos (230 mg, 0.4 mmol, 10 mol%), and $B_2(pin)_2$ (1.12 g, 4.4 mmol, 1.1 equiv.) in dry THF (0.1 M, 40 mL) at r.t. under Ar was stirred at for 5 min. NaO*t*-Bu (423 mg, 4.4 mmol, 1.1 equiv.) was added and the mixture stirred for additional 5 min at r.t. The alkyne (4.0 mmol, 1.0 equiv.) and MeI (2.27 g, 16.0 mmol, 4.0 equiv.) were added sequentially and the mixture was stirred for 24 h. The mixture was diluted with Et_2O (50 mL) and filtered through a layer of Celite. The solvent was evaporated, and the crude residue was purified by flash column chromatography to provide the pure product.

General Procedure for the preparation of cinnamic acids from cinnamates – GP3

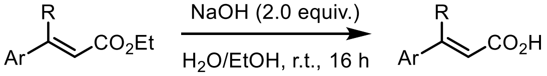

A solution of cinnamate (1 mmol, 1 eq.) in ethanol (2 mL) was treated with a 1 M aq. NaOH (2 mL) and the mixture stirred for 16 h at r.t.. The resulting solution was acidified to pH = 1 using 2 M aq. HCl forming a visible precipitate that was isolated via filtration, washed with H_2O and dried under high vacuum to give desired cinnamic acid without further purification.

General Procedure for the synthesis of 1,5-dienes – GP4

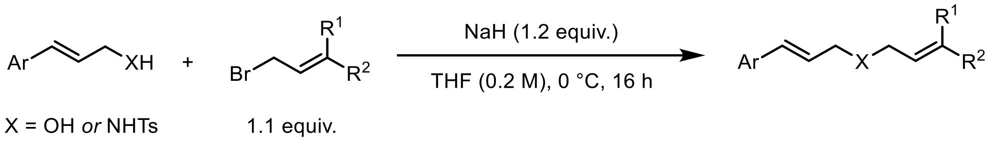

A solution of the nucleophile (3.0 mmol, 1.0 equiv.) in dry THF (0.33 M) at 0 °C was treated with NaH (60% in mineral oil, 144 mg, 3.6 mmol, 1.2 equiv.) and the mixture stirred for 1 h at 0 °C. The allylic bromide (3.3 mmol, 1.1 equiv.) was added dropwise as a solution in dry THF (6 mL) at 0 °C. The mixture was warmed up to r.t. and stirred for 16 h. The reaction was diluted with sat. aq. NH_4Cl (20 mL). The phases were separated, and the aqueous layer extracted with Et_2O (2 x 20 mL). The combined organic layers were washed with brine (20 mL), dried ($MgSO_4$), filtered and evaporated. The residue was purified by flash column chromatography to give pure compounds.

General Procedure for preparation of vinyl cinnamyl ethers from cinnamaldehydes- GP5

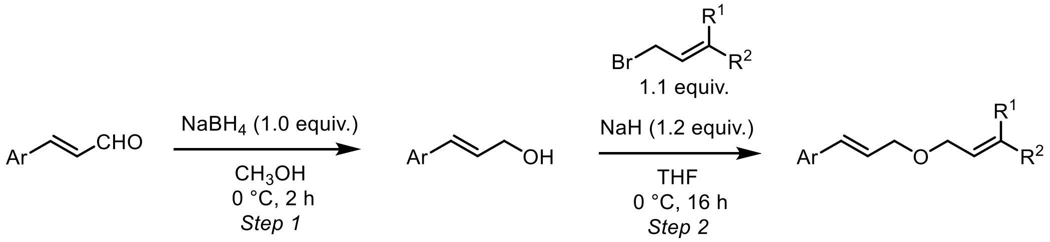

Step 1 – A solution of the cinnamaldehyde starting material (3.0 mmol, 1.0 equiv.) in MeOH (6 mL) was cooled to 0 °C and treated with $NaBH_4$ (113 mg, 3.0 mmol, 1.0 equiv.). The mixture was warmed to r.t. and stirred for 2 h. The mixture was diluted with Et_2O (20 mL), cooled to 0 °C and treated with sat. aq. NH_4Cl (20 mL). The phases were separated, and the aqueous layer extracted with Et_2O (2 x 20 mL). The combined organic layers were washed with brine (30 mL), dried ($MgSO_4$), filtered and evaporated. The crude product was used in the next step without further purification.
Step 2 – see **GP4**.

General Procedure for the isomerization of α,β-unsaturated esters – GP6

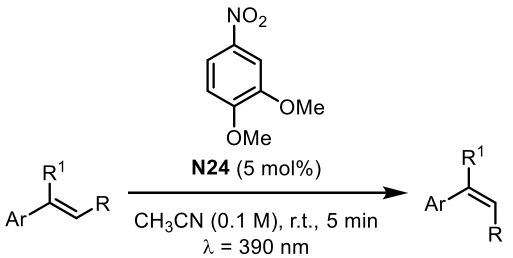

An oven-dried microwave vial containing a stir-bar was charged with the *E*-isomer (0.1 mmol, 1.0 equiv.) and **N24** (1 mg, 5 mol%). The vial was capped with a Supelco aluminium crimp seal with septum (PTFE/butyl) and was evacuated and refilled with Ar (x 3). Dry and degassed CH_3CN (1.0 mL, 0.1 M) was added and the reaction mixture was stirred (>500 rpm) under irradiation with a 390 nm Kessil LED lamp (5 cm distance, 100% intensity, fan on) for 5 min. A solution of 1,3-

dinitrobenzene (0.5 mL of 0.2 M $CDCl_3$ solution) was added to the reaction mixture. An aliquot (100 µL) of the mixture was transferred to a NMR tube, diluted with 600 µL of $CDCl_3$ and analyzed using quantitative ^1H NMR to obtain the ^1H NMR yield and the *E*/*Z* ratio. The solvent was evaporated and the residue purified by flash column chromatography to give the pure product.

General Procedure for the isomerization of α-substituted styrene boronic acid pinacol esters – GP7

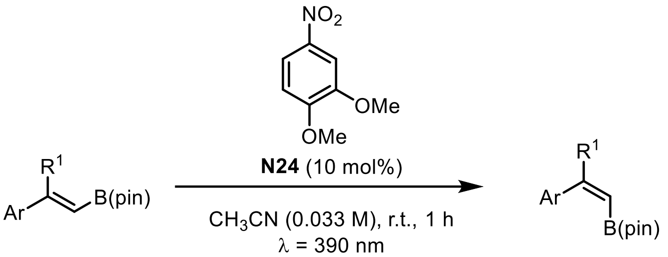

An oven-dried microwave vial containing a stir-bar was charged with the *E*-isomer (0.1 mmol, 1.0 equiv.) and **N24** (2 mg, 10 mol%). The vial was capped with a Supelco aluminium crimp seal with septum (PTFE/butyl) and was evacuated and refilled with Ar (x 3). Dry and degassed CH_3CN (3.0 mL, 0.033 M) was added and the reaction mixture was stirred (>500 rpm) under irradiation with a 390 nm Kessil LED lamp (5 cm distance, 100% intensity, fan on) for 1 h. A solution of 1,3-dinitrobenzene (0.5 mL of 0.2 M $CDCl_3$ solution) was added to the reaction mixture. An aliquot (100 µL) of the mixture was transferred to a NMR tube, diluted with 600 µL of $CDCl_3$ and analyzed using quantitative ^1H NMR to obtain NMR yield and *E*/*Z* ratio. The solvent was evaporated and the residue purified by flash column chromatography to give the pure product.

General Procedure for the intramolecular [2+2] cycloaddition – GP8

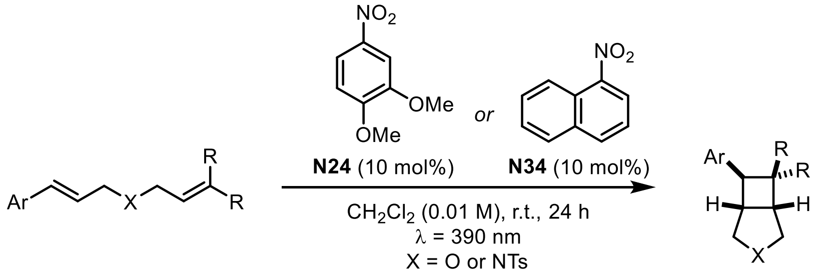

An oven-dried microwave vial containing a stir-bar was charged with 1,6-diene (0.05 mmol, 1.0 equiv.) and **N24** or **N34** (10 mol%). The vial was capped with a Supelco aluminium crimp seal with septum (PTFE/butyl) and was evacuated and refilled with Ar (x 3). Dry and degassed CH_2Cl_2 (0.01 M) was added, and the reaction mixture was stirred (>500 rpm) under irradiation with a 390 nm Kessil LED lamp (5 cm distance, 100% intensity, fan on) for 24 h. A solution of 1,3-dinitrobenzene (250 µL of 0.2 M CH_3CN solution) was added to the reaction mixture and the sample was concentrated. The residue was dissolved in 700 µL of $CDCl_3$ and analyzed using quantitative ^1H NMR to obtain NMR yield and the diastereoselectivity. The solvent was evaporated and the residue purified by flash column chromatography to give the pure product.

Supplementary Note 3: Preparation of Starting Materials

1,2-Dimethoxy-3-nitrobenzene (N20)

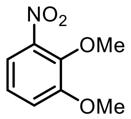

A stirred mixture of 2-methoxy-6-nitrophenol (85 mg, 0.5 mmol, 1 equiv.), K_2CO_3 (104 mg, 0.75 mmol, 1.5 equiv.), in DMF (1 mL) at r.t. was treated with Me_2SO_4 (70 µL, 0.75 mmol, 1.5 equiv.). The mixture was warmed to 75 °C and stirred for 16 h. The reaction was cooled down to r.t. then H_2O (7 mL) was added and the resulting mixture stirred for 5 min. To the resulting suspension Et_2O (15 mL) was added, phases separated, and the aqueous phase extracted with Et_2O (2 x 15 mL). The combined organics were washed with H_2O (5 x 15 mL), dried (Na_2SO_4), filtered and evaporated to give **N20** (82 mg, 90%) as a solid. R_f 0.39 [1:1 CH_2Cl_2/pentane]. ^{1}H NMR ($CDCl_3$, 400 MHz) δ 7.32 (1H, dd, J = 7.0, 2.6 Hz), 7.17–7.06 (2H, m), 3.98 (3H, s), 3.92 (3H, s); ^{13}C NMR ($CDCl_3$, 101 MHz) δ 154.3, 145.2, 143.0, 123.8, 116.2, 116.1, 62.1, 56.6. HRMS (ESI) Found $[M + Na]^+$ 206.0425, $C_8H_9O_4NNa$ requires 206.0424.

1,3-Dimethoxy-5-nitrobenzene (N25)

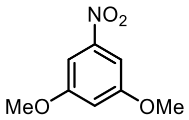

A solution of 3,5-dimethoxyaniline (766 mg, 5 mmol, 1 equiv.) and K_2CO_3 (691 mg, 5 mmol, 1 equiv.) in CH_3CN (15 mL) was treated with aq. H_2O_2 (30% v/v, 6.13 mL, 12 equiv.) and the mixture was stirred for 4 h at 40 °C. The mixture was cooled to r.t. and evaporated. The residue was purified using flash column chromatography to give **N25** (290 mg, 32%) as a solid. ^{1}H NMR ($CDCl_3$, 600 MHz) δ 7.36 (2H, d, J = 2.3 Hz), 6.74 (1H, t, J = 2.3 Hz), 3.86 (6H, s); ^{13}C NMR ($CDCl_3$, 151 MHz) δ 161.2, 150.2, 107.6, 101.8, 56.3. Data in accordance with the literature.[6]

5-Nitroisophthalonitrile (N32)

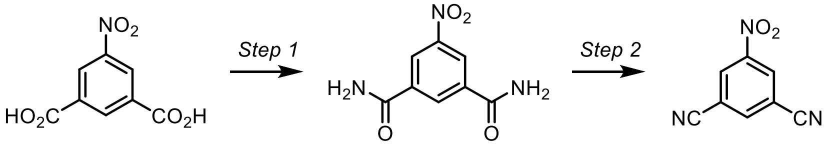

Step 1 – A solution of 5-nitroisophthalic acid (5.0 g, 23.7 mmol, 1 equiv.) in DMF (0.25 mL) was treated with $SOCl_2$ (3.5 mL), slowly heated up to 90 °C and stirred for 2 h. An additional portion of $SOCl_2$ (1.2 mL) was added and the mixture was stirred for 1 h. Excess $SOCl_2$ was removed by short path distillation and the crude acid chloride was added to a cooled solution of aq. NH_3 (30 mL, 25%) and stirred for 1 h at r.t. The precipitated solids were filtered, washed with H_2O (3 x 20 mL) and oven dried (110 °C) to give **S1** (4.45 g, 90%) as a solid. ^{1}H NMR (DMSO-d_6, 400 MHz) δ 8.84 (1H, s), 8.80 (2H,

s), 8.44 (2H, s), 7.79 (2H, s); ^{13}C NMR (DMSO-d_6, 101 MHz) δ 165.4, 148.0, 136.1, 132.7, 124.6. Found $[M + H]^+$ 210.0511, $C_8H_8O_4N_3$ requires 210.0509.

Step 2 – A solution of **S1** (4.0 g, 19.1 mmol, 1 equiv.) in DMF (10 mL) under Ar at r.t. was treated dropwise with $POCl_3$ (4.6 mL, 49.7 mmol, 2.6 equiv.) and stirred for 3 h at 45 °C. The reaction was quenched by adding dropwise to 150 mL of ice water and stirred until the ice melted. The precipitated solids were collected via filtration and oven dried (110 °C) to give **N32** (2.9 g, 88%) as a solid. ^1H NMR (DMSO-d_6, 400 MHz) δ 9.07 (2H, d, J = 1.1 Hz), 8.91 (1H, t, J = 1.2 Hz); ^{13}C NMR (DMSO-d_6, 101 MHz) δ 148.2, 142.1, 131.8, 115.7, 114.2. Found $[M]^+$ 173.0219, $C_8H_3O_2N_3$ requires 173.0220. Data in accordance with the literature.[7]

Ethyl (*E*)-3-Phenylpent-2-enoate (*E*-1)

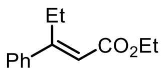

Following **GP1**, propiophenone (537 mg, 4.0 mmol, 1.0 equiv.) gave ***E*-1** (433 mg, 53%) as an oil. ^1H NMR (600 MHz, $CDCl_3$) δ 7.44 (2H, dd, J = 7.4, 2.1 Hz), 7.40–7.32 (3H, m), 6.02 (1H, s), 4.21 (2H, q, J = 7.1 Hz), 3.11 (2H, q, J = 7.5 Hz), 1.32 (3H, t, J = 7.1 Hz), 1.08 (3H, t, J = 7.5 Hz); ^{13}C NMR (151 MHz, $CDCl_3$) δ 166.6, 162.2, 141.3, 129.0, 128.6, 126.8, 116.9, 59.9, 24.5, 14.5, 13.7. Data in accordance with the literature.[8]

Ethyl (*E*)-3-Phenylbut-2-enoate (*E*-3)

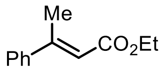

Following **GP1**, acetophenone (480 mg, 4.0 mmol, 1.0 equiv.) gave ***E*-3** (387 mg, 51%) as an oil. ^1H NMR (600 MHz, $CDCl_3$) δ 7.50–7.45 (2H, m), 7.40–7.32 (3H, m), 6.14 (1H, q, J = 1.3 Hz), 4.22 (2H, q, J = 7.1 Hz), 2.58 (3H, d, J = 1.3 Hz), 1.32 (3H, t, J = 7.1 Hz); ^{13}C NMR (151 MHz, $CDCl_3$) δ 167.0, 155.6, 142.4, 129.1, 128.6, 126.4, 117.3, 60.0, 18.1, 14.5. Data in accordance with the literature.[8]

Ethyl (*Z*)-4,4,4-Trifluoro-3-phenylbut-2-enoate (*Z*-4)

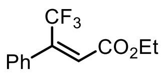

Following **GP1** but with a reaction time of 2 h, 2,2,2-trifluoroacetophenone (696 mg, 4.0 mmol, 1.0 equiv.) gave ***Z*-4** (107 mg, 11%) as an oil. ^1H NMR (600 MHz, $CDCl_3$) δ 7.44–7.38 (5H, m), 6.34 (1H, s), 4.31 (2H, q, J = 7.2 Hz), 1.35 (3H, t, J = 7.2 Hz); ^{13}C NMR (151 MHz, $CDCl_3$) δ 164.8, 138.0 (q, J = 31.8 Hz), 133.7, 129.7, 128.8, 127.9, 127.7 (q, J = 3.6 Hz), 122.4 (q, J = 275.7 Hz), 61.9, 14.1; ^{19}F NMR (564 MHz, $CDCl_3$) δ –60.10 (s). Data in accordance with the literature.[8]

Ethyl (*E*)-3-(Pyridin-3-yl)but-2-enoate (*E*-5)

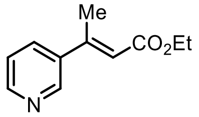

Following **GP1**, 3-acetylpyridine (484 mg, 4.0 mmol, 1.0 equiv.) gave ***E*-5** (530 mg, 69%) as an oil. ^{1}H NMR (600 MHz, $CDCl_3$) δ 8.71 (1H, s), 8.57 (1H, dd, *J* = 4.7, 1.7 Hz), 7.73 (1H, dd, *J* = 8.0, 2.2 Hz), 7.29 (1H, dd, *J* = 8.0, 4.8 Hz), 6.12 (1H, q, *J* = 1.4 Hz), 4.21 (2H, q, *J* = 7.2 Hz), 2.56 (3H, d, *J* = 1.3 Hz), 1.30 (3H, t, *J* = 7.1 Hz); ^{13}C NMR (151 MHz, $CDCl_3$) δ 166.4, 152.1, 150.1, 147.6, 137.8, 133.7, 123.3, 118.8, 60.2, 17.8, 14.4. Data in accordance with the literature.[8]

Ethyl (*E*)-3-(4-Methoxyphenyl)but-2-enoate (*E*-6)

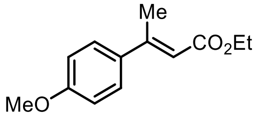

Following **GP1**, 4′-methoxyacetophenone (600 mg, 4.0 mmol, 1.0 equiv.) gave ***E*-6** (379 mg, 43%) as an oil. ^{1}H NMR (600 MHz, $CDCl_3$) δ 7.45 (2H, dd, *J* = 8.7, 1.6 Hz), 6.89 (2H, dd, *J* = 8.7, 1.6 Hz), 6.11 (1H, s), 4.21 (2H, q, *J* = 7.1 Hz), 3.82 (3H, s), 2.56 (3H, s), 1.31 (3H, t, *J* = 7.1 Hz); ^{13}C NMR (151 MHz, $CDCl_3$) δ 167.2, 160.6, 155.0, 134.5, 127.8, 115.5, 113.9, 59.8, 55.4, 17.8, 14.5. Data in accordance with the literature.[9]

Ethyl (*E*)-3-(3-Bromo-2-fluorophenyl)but-2-enoate (*E*-7)

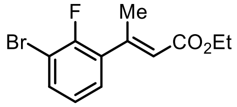

Following **GP1**, 1-(3-bromo-2-fluorophenyl)ethanone (868 mg, 4.0 mmol, 1.0 equiv.) gave ***E*-7** (301 mg, 26%) as an oil. R_f 0.60 [pentane:Et_2O (30:1)]; ^{1}H NMR (600 MHz, $CDCl_3$) δ 7.51 (1H, dd, *J* = 8.1, 6.3 Hz), 7.19 (1H, dd, *J* = 8.0, 6.4 Hz), 7.01 (1H, t, *J* = 7.8 Hz), 5.97 (1H, s), 4.22 (2H, q, *J* = 7.2 Hz), 2.50 (3H, s), 1.31 (3H, t, *J* = 7.1 Hz); ^{13}C NMR (151 MHz, $CDCl_3$) δ 166.2, 155.9 (d, *J* = 249.9 Hz), 151.0, 133.6, 132.7 (d, *J* = 14.7 Hz), 128.4 (d, *J* = 3.1 Hz), 125.2 (d, *J* = 4.3 Hz), 121.4 (d, *J* = 2.4 Hz), 110.1 (d, *J* = 21.9 Hz), 60.2, 19.5 (d, *J* = 3.6 Hz), 14.4; ^{19}F NMR (564 MHz, $CDCl_3$) δ –107.88 (t, *J* = 6.5 Hz); HRMS (ESI) found $[M+Na]^+$ 308.98910, $C_{12}H_{12}O_2BrFNa$ requires 308.98969.

Ethyl (*E*)-3-(4-Bromophenyl)pent-2-enoate (*E*-8)

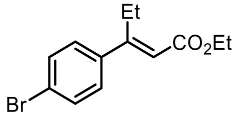

Following **GP1**, 4′-bromopropiophenone (852 mg, 4.0 mmol, 1.0 equiv.) gave ***E*-8** (512 mg, 45%) as an oil. ^{1}H NMR (600 MHz, $CDCl_3$) δ 7.49 (2H, dd, *J* = 8.4, 1.6 Hz), 7.35–7.25 (2H, dd, *J* = 8.4, 1.6 Hz), 5.98 (1H, s), 4.21 (2H, q, *J* = 7.1 Hz),

3.07 (2H, q, J = 7.6 Hz), 1.31 (3H, t, J = 7.1 Hz), 1.05 (3H, t, J = 7.5 Hz); ^{13}C NMR (151 MHz, $CDCl_3$) δ 166.3, 160.7, 140.1, 131.8, 128.5, 123.2, 117.4, 60.1, 24.3, 14.4, 13.6. Data in accordance with the literature.[10]

(*E*)-4,4,5,5-Tetramethyl-2-(2-phenylbut-1-en-1-yl)-1,3,2-dioxaborolane (*E*-9)

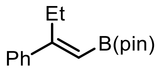

Following **GP2** but with iodoethane (1.25 g, 8.0 mmol, 2.0 equiv.) instead of iodomethane, phenylacetylene (408 mg, 4.0 mmol, 1.0 equiv.) gave ***E*-9** (77 mg, 8%) as an oil. ^{1}H NMR (600 MHz, $CDCl_3$) δ 7.46–7.43 (2H, m), 7.33–7.29 (2H, m), 7.29–7.26 (1H, m), 5.61 (1H, s), 2.90 (2H, q, J = 7.5 Hz), 1.31 (12H, s), 1.02 (3H, t, J = 7.5 Hz); ^{13}C NMR (151 MHz, $CDCl_3$) δ 165.1, 143.2, 128.3, 127.9, 126.5, 83.0, 26.8, 25.0, 14.9 (the boron-bearing carbon was not observed due to fast quadrupole relaxation); ^{11}B NMR (193 MHz, $CDCl_3$) δ 30.56. Data in accordance with the literature.[11]

(*E*)-4,4,5,5-Tetramethyl-2-(2-phenylprop-1-en-1-yl)-1,3,2-dioxaborolane (*E*-10)

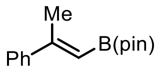

Following **GP2**, phenylacetylene (408 mg, 4.0 mmol, 1.0 equiv.) gave ***E*-10** (400 mg, 41%) as an oil. ^{1}H NMR (600 MHz, $CDCl_3$) δ 7.53–7.45 (2H, m), 7.35–7.31 (2H, m), 7.30–7.27 (1H, m), 5.76 (1H, q, J = 1.0 Hz), 2.41 (3H, d, J = 1.0 Hz), 1.32 (12H, s); ^{13}C NMR (151 MHz, $CDCl_3$) δ 157.9, 144.0, 128.3, 128.1, 126.0, 83.1, 25.0, 20.2 (the boron-bearing carbon was not observed due to fast quadrupole relaxation); ^{11}B NMR (193 MHz, $CDCl_3$) δ 30.21. Data in accordance with the literature.[11]

(*E*)-2-(2-(4-Bromophenyl)prop-1-en-1-yl)-4,4,5,5-tetramethyl-1,3,2-dioxaborolane (*E*-11)

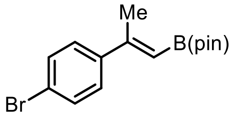

Following **GP2**, 1-bromo-4-ethynylbenzene (724 mg, 4.0 mmol, 1.0 equiv.) gave ***E*-11** (492 mg, 38%) as an oil. ^{1}H NMR (600 MHz, $CDCl_3$) δ 7.44 (2H, d, J = 8.5 Hz), 7.35 (2H, d, J = 8.5 Hz), 5.73 (1H, s), 2.37 (3H, s), 1.31 (12H, s); ^{13}C NMR (151 MHz, $CDCl_3$) δ 156.5, 142.8, 131.4, 127.6, 122.1, 83.2, 25.0, 20.1 (the boron-bearing carbon was not observed due to fast quadrupole relaxation); ^{11}B NMR (193 MHz, $CDCl_3$) δ 29.94. Data in accordance with the literature.[11]

(*E*)-4,4,5,5-Tetramethyl-2-(2-(4-(trifluoromethyl)phenyl)prop-1-en-1-yl)-1,3,2-dioxaborolane (*E*-12)

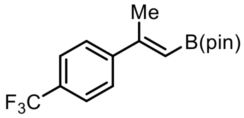

Following **GP2**, 1-ethynyl-4-(trifluoromethyl)benzene (680 mg, 4.0 mmol, 1.0 equiv.) gave ***E*-12** (412 mg, 33%) as an oil. ^{1}H NMR (600 MHz, $CDCl_3$) δ 7.60–7.54 (4H, m), 5.79 (1H, s), 2.41 (3H, s), 1.32 (12H, s); ^{13}C NMR (151 MHz, $CDCl_3$) δ

156.3, 147.5, 129.9 (q, J = 32.5 Hz), 126.3, 125.3 (q, J = 3.7 Hz), 124.8 (q, J = 271.93 Hz), 83.3, 25.0, 20.2 (the boron-bearing carbon was not observed due to fast quadrupole relaxation); ^{11}B NMR (193 MHz, $CDCl_3$) δ 29.50; ^{19}F NMR (564 MHz, $CDCl_3$) δ –62.49 (s). Data in accordance with the literature.[12]

(*E*)-4,4,5,5-Tetramethyl-2-(2-(m-tolyl)prop-1-en-1-yl)-1,3,2-dioxaborolane (*E*-13)

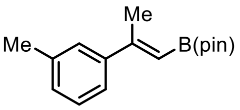

Following **GP2**, 1-ethynyl-3-methylbenzene (465 mg, 4.0 mmol, 1.0 equiv.) gave ***E*-13** (569 mg, 55%) as an oil. ^{1}H NMR (600 MHz, $CDCl_3$) δ 7.35–7.29 (2H, m), 7.22 (1H, t, J = 7.6 Hz), 7.11 (1H, d, J = 7.8 Hz), 5.76 (1H, s), 2.41 (3H, s), 2.36 (3H, s), 1.32 (12H, s); ^{13}C NMR (151 MHz, $CDCl_3$) δ 158.1, 144.0, 137.7, 128.8, 128.2, 126.8, 123.1, 83.0, 25.0, 21.6, 20.3 (the boron-bearing carbon was not observed due to fast quadrupole relaxation); ^{11}B NMR (193 MHz, $CDCl_3$) δ 30.37. Data in accordance with the literature.[11]

Ethyl (*E*)-3-(Pyridin-2-yl)acrylate (*E*-16)

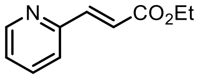

A mixture of triethyl phosphonoacetate (2.2 mL, 11.0 mmol, 1.1 equiv.), LiCl (466 mg, 11.0 mmol, 1.1 equiv.), and DIPEA (1.9 mL, 11.0 mmol, 1.1 equiv.) in dry CH_3CN (33 mL) under Ar at r.t. was stirred for 30 min. 2-pyridinecarboxaldehyde (0.95 mL, 10.0 mmol, 1.0 equiv.) was added slowly as a solution in dry CH_3CN (33 mL) and the reaction mixture stirred for 24 h at r.t. The mixture was diluted with H_2O (40 mL) and EtOAc (30 mL). The phases were separated, and the aqueous layer extracted with EtOAc (2 x 30 mL). The combined organic layers were washed with brine (40 mL), dried ($MgSO_4$), filtered and evaporated. The residue was purified via flash column chromatography to give ***E*-16** (1.54 g, 87%) as an oil. ^{1}H NMR (600 MHz, $CDCl_3$) δ 8.64 (1H, ddd, J = 4.8, 1.9, 0.9 Hz), 7.70 (1H, ddd, J = 7.8, 7.6, 1.9 Hz), 7.68 (1H, d, J = 15.8 Hz), 7.42 (1H, ddd, J = 7.8, 1.1, 0.9 Hz), 7.26 (1H, ddd, J = 7.6, 4.8, 1.1 Hz), 6.91 (1H, d, J = 15.7 Hz), 4.27 (2H, q, J = 7.1 Hz), 1.33 (3H, t, J = 7.1 Hz). Data in accordance with the literature.[13]

(*E*)-3-Phenylpent-2-enal (*E*-18)

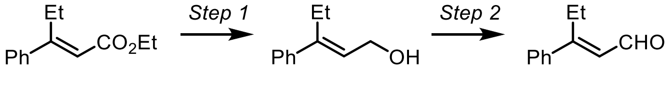

***E*-1** **S2** **17**

Step 1 – A stirred solution of ***E*-1** (204 mg, 1.0 mmol. 1.0 equiv.) in dry THF (3 ml) under Ar at –78 °C was treated dropwise with DIBAL-H (1.2 M in $PhCH_3$, 1.8 mL, 2.2 mmol, 2.2 equiv.) and the mixture stirred at –78 °C for 4 h then at r.t. for 3 h. The reaction mixture was quenched with sat. aq. NH_4Cl (10 mL), phases separated and the aqueous phase extracted with Et_2O (3 x 15 mL). Combined organics were dried over Na_2SO_4, filtered and evaporated to give crude product which was purified via flash column chromatography to give **S2** (146 mg, 90%) as an oil. ^{1}H NMR ($CDCl_3$, 400 MHz) δ 7.39–7.35

(2H, m), 7.32 (2H, t, J = 7.4 Hz), 7.29–7.23 (1H, m), 5.84 (1H, t, J = 6.8 Hz), 4.35 (2H, d, J = 6.0 Hz), 2.55 (2H, q, J = 7.5 Hz), 1.45 (1H, s), 0.99 (3H, t, J = 7.5 Hz); ^{13}C NMR ($CDCl_3$, 101 MHz) δ 145.1, 142.1, 128.4, 127.3, 126.5, 126.3, 59.8, 23.4, 14.1. Data in accordance with the literature.[14]

Step 2 – A solution of **S2** (146 mg, 0.9 mmol, 1 equiv.) in dry CH_2Cl_2 (10 mL) under Ar was treated with 1,1,1-Triacetoxy-1λ5,2-benziodoxol-3(1H)-on (DMP) (496 mg, 1.17 mmol, 1.3 equiv.) and the resulting mixture stirred at r.t. for 16 h. Volatiles were evaporated and the resulting residue treated with pentane (15 mL) while stirring until visible solids have formed. The solids were filtered off and the filtrated evaporated to give crude product which was purified via flash column chromatography to give ***E*-18** (100 mg, 69%) as an oil. ^{1}H NMR ($CDCl_3$, 400 MHz) δ 10.16 (1H, d, J = 8.0 Hz), 7.54–7.47 (2H, m), 7.45–7.39 (3H, m), 6.25 (1H, d, J = 8.0 Hz), 3.07 (2H, q, J = 7.6 Hz), 1.18 (3H, t, J = 7.6 Hz); ^{13}C NMR ($CDCl_3$, 101 MHz) δ 191.3, 165.4, 139.7, 130.1, 129.0, 126.8, 115.8, 31.9, 15.1. Data in accordance with the literature.[15]

(*E*)-3-Phenylbut-2-enenitrile (*E*-19)

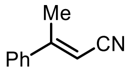

Following **GP1**, acetophenone (1.2 mL, 10 mmol, 1.0 equiv.), diethyl cyanomethylphosphonate (1.9 mL, 12 mmol, 1.2 equiv.) and NaH (60% in mineral oil, 480 mg, 12 mmol, 1.2 equiv.) gave ***E*-19** (1.14 g, 80%) as an oil. ^{1}H NMR ($CDCl_3$, 400 MHz) δ 7.53–7.36 (5H, m), 5.62 (1H, q, J = 1.0 Hz), 2.48 (3H, d, J = 1.1 Hz); ^{13}C NMR ($CDCl_3$, 101 MHz) δ 159.9, 138.4, 130.4, 129.0, 126.0, 117.8, 95.7, 20.3. Data in accordance with the literature.[16]

(*E*)-3-Phenylpent-2-enoic Acid (*E*-20)

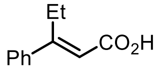

Following **GP3**, but using 1.25 M aq. KOH (2 mL), ***E*-1** (102 mg, 0.5 mmol, 1 equiv.) gave ***E*-20** (56 mg, 64%) as a white solid. ^{1}H NMR (MeOD-d_4, 400 MHz) δ 7.49–7.41 (2H, m), 7.41–7.34 (3H, m), 5.98 (1H, s), 3.10 (2H, q, J = 7.4 Hz), 1.03 (3H, t, J = 6.8 Hz); ^{13}C NMR (MeOD-d_4, 101 MHz) δ 170.1, 163.2, 142.5, 130.0, 129.7, 127.8, 118.1, 25.0, 13.9. Data in accordance with the literature.[8]

(*E*)-3-(4-Bromophenyl)pent-2-enoic Acid (*E*-22)

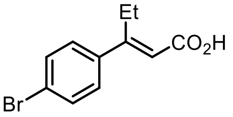

Following **GP3**, ***E*-7** (283 mg, 1 mmol, 1 equiv.) gave ***E*-22** (220 mg, 86%) as a white solid. ^{1}H NMR (MeOD-d_4, 400 MHz) δ 7.57 (2H, d, J = 8.5 Hz), 7.41 (2H, d, J = 8.5 Hz), 6.03 (1H, s), 3.11 (2H, q, J = 7.5 Hz), 1.05 (3H, t, J = 7.5 Hz); ^{13}C NMR (MeOD-d_4, 101 MHz) δ 169.4, 161.9, 141.4, 132.8, 129.7, 124.0, 118.4, 24.8, 13.8. HRMS (APCI) Found $[M + H]^+$ 255.0011, $C_8H_9O_4NNa$ requires 255.0021.

(*E*)-3-(3-Bromo-2-fluorophenyl)but-2-enoic Acid (*E*-23)

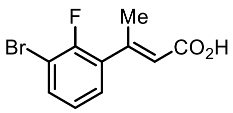

Following **GP3**, ***E*-6** (287 mg, 1 mmol, 1 equiv.) gave ***E*-23** (197 mg, 76%) as a white solid. ^{1}H NMR (MeOD-*d*$_4$, 600 MHz) δ 7.59 (1H, ddd, *J* = 8.0, 6.6, 1.5 Hz), 7.30 (2H, ddd, *J* = 8.2, 6.7, 1.6 Hz), 7.11 (2H, dt, *J* = 7.9, 0.9 Hz), 5.94 (1H, s), 2.45 (4H, t, *J* = 1.4 Hz); ^{13}C NMR (MeOD-*d*$_4$, 151 MHz) δ 169.2, 156.9 (d, *J* = 248.2 Hz), 151.9, 134.8, 133.9 (d, *J* = 15.0 Hz), 129.8 (d, *J* = 2.7 Hz), 126.7 (d, *J* = 4.4 Hz), 122.7, 110.6 (d, *J* = 21.5 Hz), 19.6 (d, *J* = 3.5 Hz); ^{19}F NMR (MeOD-*d*$_4$, 564 MHz) δ -110.7 (t, *J* = 6.8 Hz). HRMS (APCI) Found [M + H]$^+$ 258.9761, $C_8H_9O_4NNa$ requires 258.9770.

Ethyl (*E*)-2-Methyl-3-phenylacrylate (*E*-24)

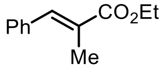

A solution of benzaldehyde (1.0 mL, 10 mmol, 1.0 equiv.) and Carbethoxyethylidene)triphenylphosphorane (4.3 g, 12 mmol, 1.2 equiv.) in CH_2Cl_2 (20 mL) under argon was stirred for 16 h at r.t.. The resulting mixture was concentrated then Et_2O (50 mL) was added and the mixture stirred for 10 minutes at r.t. to produce a white precipitate. Solids were filtered and the filtrate evaporated to give crude product which was purified via flash column chromatography to give ***E*-24** (1.8 g, 96%) as an oil. ^{1}H NMR ($CDCl_3$, 600 MHz) δ 7.69 (1H, q, *J* = 1.8 Hz), 7.42–7.37 (4H, m), 7.34–7.30 (1H, m), 4.28 (2H, q, *J* = 7.1 Hz), 2.12 (3H, d, *J* = 1.4 Hz), 1.36 (3H, t, *J* = 7.1 Hz); ^{13}C NMR ($CDCl_3$, 151 MHz) δ 168.8, 138.8, 136.1, 129.8, 128.8, 128.5, 128.4, 61.0, 14.5, 14.2. Data in accordance with the literature.[17]

(*E*)-(3-(Allyloxy)prop-1-en-1-yl)benzene (27)

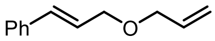

Following **GP4**, cinnamyl alcohol (1.34 g, 10.0 mmol, 1.0 equiv.), allyl bromide (0.95 mL, 11.0 mmol, 1.1 equiv.) gave **27** (1.38 g, 79%) as an oil. ^{1}H NMR (400 MHz, $CDCl_3$) δ 7.42 (2H, d, *J* = 7.6 Hz), 7.34 (2H, t, *J* = 7.5 Hz), 7.27 (1H, d, *J* = 7.4 Hz), 6.64 (1H, d, *J* = 15.9 Hz), 6.33 (1H, dt, *J* = 16.0, 6.0 Hz), 5.98 (1H, ddt, *J* = 16.4, 10.9, 5.8 Hz), 5.34 (1H, dt, *J* = 17.1, 1.7 Hz), 5.24 (1H, dt, *J* = 10.4, 1.5 Hz), 4.19 (2H, d, *J* = 5.8 Hz), 4.07 (2H, d, *J* = 5.6 Hz); ^{13}C NMR (101 MHz, $CDCl_3$) δ 136.9, 134.9, 132.6, 128.7, 127.8, 126.7, 126.2, 117.3, 71.3, 70.9. Data in accordance with the literature.[18]

(*E*)-(3-((3-Methylbut-2-en-1-yl)oxy)prop-1-en-1-yl)benzene (28)

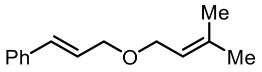

Following **GP4**, cinnamyl alcohol (1.34 g, 10.0 mmol, 1.0 equiv.) and 3,3-dimethylallylbromide (90%, 1.4 mL, 11.0 mmol, 1.1 equiv.) gave **28** (1.49 g, 74%) as an oil. ^{1}H NMR (600 MHz, $CDCl_3$) δ 7.41–7.37 (2H, d, *J* = 8.3 Hz), 7.32 (2H, dd, *J* = 8.3, 7.0 Hz), 7.24 (1H, t, *J* = 7.2 Hz), 6.62 (1H, dt, *J* = 16.0, 1.5 Hz), 6.32 (1H, dt, *J* = 15.9, 6.1 Hz), 5.40 (1H, tq, *J* = 6.9, 1.6 Hz), 4.14 (2H, dt, *J* = 6.1, 1.3 Hz), 4.03 (2H, d, *J* = 6.9 Hz), 1.77 (3H, s), 1.70 (3H, d, *J* = 1.6 Hz); ^{13}C NMR (151

MHz, $CDCl_3$) δ 137.4, 137.0, 132.5, 128.7, 127.8, 126.6, 126.6, 121.2, 70.8, 66.8, 26.0, 18.3. Data in accordance with the literature.[19]

(*E*)-1-Methoxy-4-(3-((3-methylbut-2-en-1-yl)oxy)prop-1-en-1-yl)benzene (29)

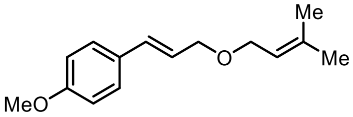

Following **GP5**, using 4-methoxycinnamaldehyde (487 mg, 3.0 mmol, 1.0 equiv.) in S*tep 1* and 3,3-dimethylallylbromide (90%, 430 µL, 3.3 mmol, 1.1 equiv.) in *Step 2* gave **29** (515 mg, 74%) as an oil. ^{1}H NMR (600 MHz, $CDCl_3$) δ 7.34–7.30 (2H, m), 6.87–6.81 (2H, m), 6.55 (1H, dt, *J* = 15.9, 1.4 Hz), 6.18 (1H, dt, *J* = 15.9, 6.3 Hz), 5.43–5.36 (1H, m), 4.11 (2H, dd, *J* = 6.2, 1.4 Hz), 4.01 (2H, d, *J* = 7.0 Hz), 3.81 (3H, s), 1.76 (3H, d, *J* = 1.5 Hz), 1.69 (3H, d, *J* = 1.4 Hz); ^{13}C NMR (151 MHz, $CDCl_3$) δ 159.4, 137.3, 132.2, 129.8, 127.8, 124.3, 121.3, 114.1, 71.0, 66.7, 55.5, 26.0, 18.3. Data in accordance with the literature.[19]

(*E*)-1-(3-((3-Methylbut-2-en-1-yl)oxy)prop-1-en-1-yl)-4-(trifluoromethyl)benzene (30)

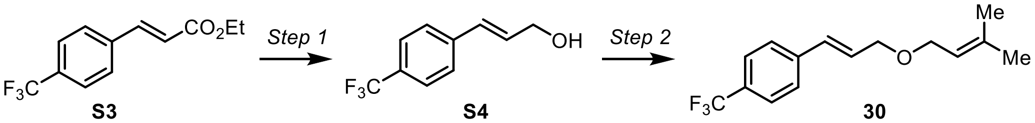

Step 1 – A solution of ethyl (*E*)-3-(4-(trifluoromethyl)phenyl)acrylate (**S3**, 691 mg, 3.0 mmol, 1.0 equiv.) in dry CH_2Cl_2 (4.5 mL) was cooled to –78 °C, treated with DIBAL-H (4.4 mL, 6.6 mmol, 2.2 equiv., 25 wt% in toluene) and stirred for 30 min. The mixture was warmed to r.t. and stirred for 2 h. The mixture was cooled to 0 °C, and diluted with 10 % aq. HCl (20 mL), H_2O (10 mL), and CH_2Cl_2 (20 mL). The phases were separated, and the aqueous layer extracted with CH_2Cl_2 (2 x 20 mL). The combined organic layers were washed with brine (40 mL), dried ($MgSO_4$), filtered and evaporated to give **S4** as a crude mixture which was used directly in the next step without further purification.

Step 2 – Following **GP4**, using **S4** (607 mg, 3.0 mmol, 1.0 equiv.) and 3,3-dimethylallylbromide (90%, 430 µL, 3.3 mmol, 1.1 equiv.) gave **30** (319 mg, 39%) as an oil. ^{1}H NMR (600 MHz, $CDCl_3$) δ 7.56 (2H, d, *J* = 8.0 Hz), 7.47 (2H, d, *J* = 8.0 Hz), 6.65 (1H, d, *J* = 16.0 Hz), 6.40 (1H, dt, *J* = 16.0, 5.8 Hz), 5.40 (1H, t, *J* = 7.0 Hz), 4.16 (2H, d, *J* = 5.7 Hz), 4.04 (2H, d, *J* = 6.9 Hz), 1.77 (3H, s), 1.70 (3H, s); ^{13}C NMR (151 MHz, $CDCl_3$) δ 140.5, 137.6, 130.6, 129.7, 129.4 (q, *J* = 32.2 Hz), 126.8, 125.7 (q, *J* = 3.9 Hz), 124.4 (d, *J* = 272.1 Hz), 121.1, 70.4, 67.1, 26.0, 18.3; ^{19}F NMR (565 MHz, $CDCl_3$) δ –62.50. Data in accordance with the literature.[20]

(*E*)-2-(3-((3-Methylbut-2-en-1-yl)oxy)prop-1-en-1-yl)furan (31)

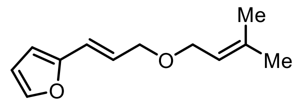

Following **GP5**, using (*E*)-3-(furan-2-yl)acrylaldehyde (366 mg, 3.0 mmol, 1.0 equiv.) in *Step 1* and 3,3-dimethylallylbromide (90%, 430 µL, 3.3 mmol, 1.1 equiv.) in *Step 2* gave **31** (319 mg, 39%) as an oil. ^{1}H NMR (600 MHz, $CDCl_3$) δ 7.34 (1H, d, *J* = 1.8 Hz), 6.43 (1H, d, *J* = 15.9), 6.38–6.34 (1H, m), 6.29–6.19 (2H, m), 5.38 (1H, tqq, *J* = 7.0, 1.4, 1.3 Hz), 4.10 (2H, dd, *J* = 5.9, 1.5 Hz), 4.00 (2H, d, *J* = 6.9 Hz), 1.76 (3H, d, *J* = 1.4 Hz), 1.68 (3H, d, *J* = 1.3 Hz); ^{13}C NMR (151 MHz, $CDCl_3$) δ 152.7, 142.1, 137.4, 125.3, 121.2, 120.5, 111.4, 108.0, 70.2, 66.8, 26.0, 18.2. Data in accordance with the literature.[19]

(*E*)-2-(3-((3-Methylbut-2-en-1-yl)oxy)prop-1-en-1-yl)pyridine (32)

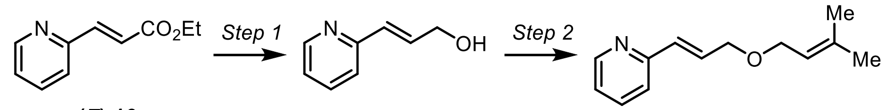

(*E*)-16 S5 32

Step 1 – A solution of ***E*-16** (1.24 g, 7.0 mmol, 1.0 equiv.) in dry CH_2Cl_2 (14 mL) wad cooled to –78 °C, treated with DIBAL-H (25 wt% in toluene, 9.9 mL, 2.1 equiv.) and stirred for 15 min. The mixture was warmed to r.t. and stirred for 5 h. The mixture was diluted with saturated aqueous solution of potassium sodium tartrate (30 mL) and H_2O (15 mL). The phases were separated, and the aqueous layer extracted with CH_2Cl_2 (3 x 30 mL). The combined organic layers were washed with brine (30 mL), dried ($MgSO_4$), filtered and evaporated to give **S5** as a crude mixture which was used directly in the next step without further purification.

Step 2 – Following **GP4**, using **S5** (611 mg, 4.5 mmol, 1.0 equiv) and 3,3-dimethylallylbromide (90%, 640 µL, 5.0 mmol, 1.1 equiv.) gave **32** (432 mg, 56%) as an oil. ^{1}H NMR (600 MHz, $CDCl_3$) δ 8.55 (1H, ddd, *J* = 4.9, 1.9, 0.9 Hz), 7.62 (1H, ddd, *J* = 7.8, 7.5, 1.8 Hz), 7.30 (1H, ddd, *J* = 7.8, 1.1 Hz, 0.9 Hz), 7.12 (1H, ddd, *J* = 7.5, 4.8, 1.1 Hz), 6.78 (1H, dt, *J* = 15.8, 5.3 Hz), 6.71 (1H, dt, *J* = 15.8, 1.4 Hz), 5.39 (1H, tdq, J = 6.9, 2.8, 1.4 Hz), 4.19 (2H, dd, *J* = 5.3, 1.4 Hz), 4.04 (2H, d, *J* = 6.9 Hz), 1.76 (3H, d, *J* = 1.3 Hz), 1.69 (3H, d, *J* = 1.3 Hz); ^{13}C NMR (151 MHz, $CDCl_3$) δ 155.5, 149.7, 137.4, 136.6, 131.5, 131.4, 122.3, 121.7, 121.2, 70.1, 67.0, 26.0, 18.3. Data in accordance with the literature.[19]

***N*-Cinnamyl-4-methyl-*N*-(3-methylbut-2-en-1-yl)benzenesulfonamide (33)**

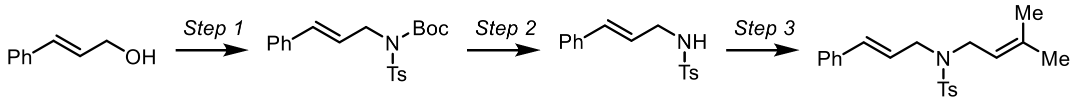

S6 S7 33

Step 1 – A solution of cinnamyl alcohol (1.34 g, 10.0 mmol, 1.0 equiv.), TsNHBoc (2.98 g, 11.0 mmol, 1.1 equiv.), and Ph_3P (5.77 g, 22.0 mmol, 2.2 equiv.) in dry THF (30 mL, 0.33 M) was treated with DIAD (4.3 mL, 22.0 mmol, 2.2 equiv.) and the mixture was stirred for 16 h. The mixture was diluted with H_2O (30 mL) and EtOAc (30 mL). The phases were separated,

and the aqueous layer extracted with EtOAc (2 x 30 mL). The combined organic layers were washed with brine (50 mL), dried ($MgSO_4$), filtered and evaporated to give, after purification using flash chromatography, **S6** (2.91 g, 75%) as a solid. ^{1}H NMR (400 MHz, $CDCl_3$) δ 7.83–7.75 (2H, m), 7.42–7.36 (2H, m), 7.36–7.30 (2H, m), 7.29–7.23 (3H, m), 6.67 (1H, d, *J* = 15.9), 6.28 (1H, dt, *J* = 15.8, 6.5 Hz), 4.60 (2H, dd, *J* = 6.5, 1.4 Hz), 2.42 (3H, s), 1.36 (9H, s); ^{13}C NMR ($CDCl_3$, 101 MHz) δ 150.9, 144.3, 137.4, 136.6, 134.0, 129.4, 128.7, 128.2, 128.0, 126.7, 124.4, 84.5, 48.7, 28.1, 21.7. Data in accordance with the literature.[21]

Step 2 – A solution of **S6** (387 mg, 1 mmol, 1.0 equiv.) in CH_2Cl_2 (3 mL) was cooled in an ice bath, treated with TFA (0.5 mL, 7 mmol, 7 equiv.) and stirred for 90 min. The mixture was partitioned between CH_2Cl_2 (10 mL) and 1 M aq. NaOH (20 mL). The phases were separated and the aqueous layer extracted with CH_2Cl_2 (2 x 20 mL). Combined organics were washed with brine (20 mL), dried (Na_2SO_4), filtered and evaporated to give **S7** (255 mg, 89%) as a solid without further purification. ^{1}H NMR ($CDCl_3$, 400 MHz) δ 7.78 (2H, d, *J* = 8.3 Hz), 7.34–7.18 (7H, m), 6.44 (1H, d, *J* = 15.8 Hz), 6.01 (1H, dt, *J* = 15.8, 6.4 Hz), 4.70–4.53 (1H, m), 3.75 (2H, td, *J* = 6.5, 1.3 Hz), 2.41 (3H, s); ^{13}C NMR ($CDCl_3$, 101 MHz) δ 143.7, 137.2, 136.2, 133.2, 129.9, 128.7, 128.1, 127.3, 126.5, 124.2, 45.6, 21.6. Data in accordance with the literature.[21]

Step 3 – Following **GP4**, **S7** (164 mg, 0.57 mmol, 1.0 equiv.) and 3,3-dimethylallylbromide (90%, 81 µL, 0.63 mmol, 1.1 equiv.) gave **33** (147 mg, 73%) as a solid. ^{1}H NMR (600 MHz, $CDCl_3$) δ 7.72 (2H, d, *J* = 8.3 Hz), 7.32–7.28 (4H, m), 7.27–7.21 (3H, m), 6.41 (1H, dt, *J* = 15.9, 1.5 Hz), 5.97 (1H, dt, *J* = 15.8, 6.6 Hz), 5.03 (1H, tqq, *J* = 7.1, 1.5, 1.4 Hz), 3.93 (2H, dd, *J* = 6.8, 1.4 Hz), 3.83 (2H, d, *J* = 7.1 Hz), 2.43 (3H, s), 1.66 (3H, d, *J* = 1.5 Hz), 1.57 (3H, d, *J* = 1.4 Hz); ^{13}C NMR (151 MHz, $CDCl_3$) δ 143.2, 137.8, 137.1, 136.6, 133.7, 129.8, 128.7, 128.0, 127.4, 126.6, 124.6, 119.1, 49.0, 44.8, 26.0, 21.7, 18.1. Data in accordance with the literature.[19]

(±)-Ethyl 7-Hydroxy-2,5-dimethyl-2-(4-methylpent-3-en-1-yl)-2H-chromene-6-carboxylate (34)

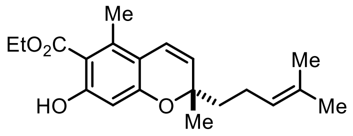

A solution of ethyl 2,4-dihydroxy-6-methylbenzoate (196 mg, 1 mmol, 1 equiv.) and citral (0.19 mL, 1.1 mmol, 1.1 equiv.) in xylene (10 mL) was treated with ethylenediamine diacetate (18 mg, 0.1 mmol, 10 mol%). The mixture was heated under reflux for 16 h then cooled to r.t. Solvent was evaporated and the residue purified using flash column chromatography to give **34** (83 mg, 23%) as a solid. ^{1}H NMR ($CDCl_3$, 600 MHz) δ 12.05 (1H, s), 6.73 (1H, d, *J* = 10.1 Hz), 6.18 (1H, s), 5.46 (1H, d, *J* = 10.1 Hz), 5.08 (1H, t, *J* = 7.1 Hz), 4.39 (2H, q, *J* = 7.1 Hz), 2.47 (3H, s), 2.08 (2H, q, *J* = 8.1 Hz), 1.74 (1H, ddd, *J* = 14.1, 9.6, 7.0 Hz), 1.65 (3H, s), 1.57 (3H, s), 1.40 (3H, t, *J* = 7.2 Hz), 1.39 (3H, s). ^{13}C NMR ($CDCl_3$, 151 MHz) δ 172.0, 159.8, 157.7, 142.8, 131.8, 126.2, 123.9, 116.8, 111.6, 107.1, 105.0, 79.6, 61.2, 41.6, 27.0, 25.6, 24.5, 22.7, 17.6, 14.2. Data in accordance with the literature.[22]

(*E*)-3-(Cinnamyloxy)propanal *O*-Benzyl Oxime (41)

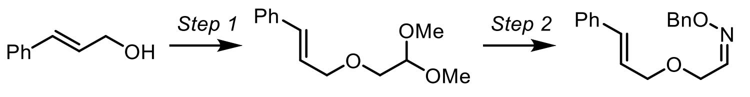

Step 1 – A stirred suspension of NaH (60% in mineral oil, 1.2 g, 30 mmol, 1.5 equiv.) in dry DMF (60 mL) under Ar at 0 °C was treated dropwise with (*E*)-cinnamyl alcohol (2.6 mL, 20 mmol, 1.0 equiv.) and the mixture stirred at 0 °C for 1 h. Then, 2-bromo-1,1-dimethoxyethane (4.0 mL, 33.8 mmol, 1.7 equiv.) was added and the reaction stirred at 110 °C for 24 h. The reaction was quenched with sat. aq. NH_4Cl (50 mL) and H_2O (20 mL). Layers were separated and the aqueous layer extracted with Et_2O (3 x 50 mL). Combined organics were washed with H_2O (4 x 50 mL), brine (2 x 50 mL), dried over Na_2SO_4 then evaporated to give crude product which was purified by flash chromatography to give **S8** (2.1 g, 48%) as a yellow oil. ^{1}H NMR ($CDCl_3$, 400 MHz) δ 7.42–7.35 (2H, m), 7.32 (2H, tt, *J* = 8.5, 1.4 Hz), 7.24 (1H, tt, *J* = 7.1, 1.3 Hz), 6.61 (1H, d, *J* = 15.9 Hz), 6.29 (1H, dt, *J* = 15.9, 6.2 Hz), 4.56 (1H, t, *J* = 5.2 Hz), 4.21 (2H, dd, *J* = 6.2, 1.4 Hz), 3.55 (2H, d, *J* = 5.2 Hz), 3.41 (6H, s); ^{13}C NMR ($CDCl_3$, 101 MHz) δ 136.7, 133.0, 128.7, 127.9, 126.7, 125.9, 102.9, 72.3, 69.8, 54.1. Data in accordance with the literature.[23]

Step 2 – A stirred solution of **S8** (445 mg, 2.0 mmol, 1.0 equiv.) in H_2O/acetone (1:1, 20 mL) under air was treated with *p*-toluenesulphonic acid monohydrate (76 mg, 20 mol%) and the resulting mixture stirred at 85 °C for 5 h. The reaction was allowed to cool to r.t. then treated with sat. aq. $NaHCO_3$ (20 mL) and extracted with Et_2O (3 x 40 mL). Combined organics were dried over Na_2SO_4 and evaporated to give crude mixture of aldehyde intermediate. The crude mixture was dissolved in CH_2Cl_2 (20 mL) and treated with NaOAc (656 mg, 8.0 mmol, 4.0 equiv.) and *O*-benzylhydroxylamine hydrochloride (638 mg, 4.0 mmol, 2 equiv.) and the mixture stirred at r.t. for 16 h. The reaction was treated with sat. aq. $NaHCO_3$ (20 mL), layers separated and the aqueous layer extracted with Et_2O (3 x 20 mL). Combined organics were dried over Na_2SO_4 and evaporated to give crude product which was purified via flash column chromatography to give **41** (480 mg, 85%) as an oil as a mixture of isomers E/Z (oxime) = 1:1.3). ^{1}H NMR ($CDCl_3$, 400 MHz) δ 7.46 (1H, t, *J* = 5.7 Hz, minor), 7.33–7.12 (23H, m, major+minor), 6.84 (1.3H, t, *J* = 3.6 Hz, major), 6.53 (1.3H, d, *J* = 15.9 Hz, major), 6.50 (1H, d, *J* = 16.0 Hz, minor), 6.17 (2.3H, dtd, *J* = 15.7, 6.1, 3.4 Hz, major+minor), 5.03 (2H, s, minor), 5.02 (2.6H, s, major), 4.28 (2.6H, d, *J* = 3.6 Hz, major), 4.07 (4.6H, td, *J* = 6.8, 1.4 Hz, major+minor), 4.04 (2H, d, J = 5.8 Hz, minor); ^{13}C NMR ($CDCl_3$, 101 MHz) δ 150.8, 147.7, 137.7, 137.5, 136.64, 136.58, 133.34, 133.30, 128.70, 128.69, 128.6, 128.5, 128.4, 128.2, 128.08, 128.06, 128.0, 127.9, 126.67, 126.66, 125.3, 125.2, 76.4, 76.2, 71.9, 71.2, 66.8, 64.6. Data in accordance with the literature.[23]

(*E*)-1-(3-((3-Methylbut-2-en-1-yl)oxy)prop-1-en-1-yl)-4-(trifluoromethyl)benzene (43)

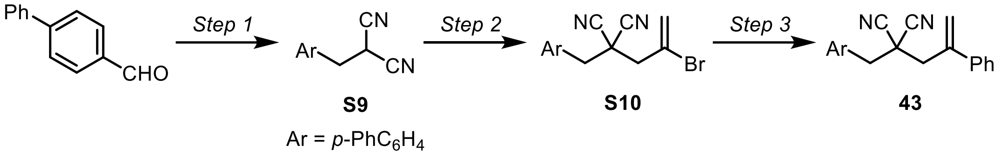

Step 1 – A stirred solution of malononitrile (661 mg, 10 mmol, 1.0 equiv.) in 95% EtOH (10 mL) was treated with *p*-phenyl benzaldehyde (1.82 g, 10 mmol, 1.0 equiv.) was added at r.t. and the mixture stirred for 16 h at r.t.. EtOH (20 mL) was added and the mixture cooled to 0 °C in an ice bath. $NaBH_4$ (169 mg, 5 mmol, 0.5 equiv.) was added and the mixture stirred for 1 h at 0 °C. To the reaction mixture was added H_2O (50 mL) and CH_2Cl_2 (25 mL). The phases were separated, and the aqueous layer extracted with CH_2Cl_2 (2 x 25 mL). The combined organic layers were dried over $MgSO_4$, filtered and evaporated to give **S9** (2.2 g, 95%) as a crude mixture which was used directly in the next step without further purification.

Step 2 – A solution of **S9** (2.2 g, 9.5 mmol, 1.0 equiv.) in dry THF (38 mL) was cooled to 0 °C and NaH (60% in mineral oil, 455 mg, 11.4 mmol, 1.2 equiv.) was added. The mixture was stirred at 0 °C for 1 h then 2,3-dibromoprop-1-ene (1.6 mL, 14.2 mmol, 1.5 eq.). was added dropwise and the mixture stirred for 18 h at r.t.. The mixture was diluted with sat. aq. NH_4Cl (50 mL), H_2O (50 mL), and EtOAc (50 mL). The phases were separated, and the aqueous layer extracted with EtOAc (2 x 50 mL). The combined organic layers were dried over $MgSO_4$, filtered and evaporated to give **S10** (3.3 g, quant.) as a crude mixture which was used directly in the next step without further purification.

Step 3 – To **S10** (3.3 g, 9.5 mmol, 1.0 equiv.), phenylboronic acid (1.74 g, 14.2 mmol, 1.5 equiv.), K_2CO_3 (3.94 g, 28.5 mmol, 3 equiv.), and $PdCl_2(PPh_3)_2$ (667 mg, 0.95 mmol, 10 mol%) were added THF (30 mL) and H_2O (10 mL). The mixture was stirred for 48 h at 50 °C. The mixture was diluted with sat. aq. NH_4Cl (50 mL) and EtOAc (50 mL). The phases were separated, and the aqueous layer extracted with EtOAc (2 x 50 mL). The combined organic layers were dried over $MgSO_4$, filtered and evaporated. The residue was purified by flash column chromatography to give **43** (2.0 g, 61%) as a solid. ^1H NMR ($CDCl_3$, 600 MHz) δ 7.62–7.57 (4H, m), 7.47–7.34 (10H, m), 5.63 (1H, s), 5.52 (1H, s), 3.21 (2H, s), 3.18 (2H, s); ^{13}C NMR ($CDCl_3$, 151 MHz) δ 141.9, 141.2, 140.4, 139.9, 131.1, 130.9, 129.0, 129.0, 128.8, 127.82, 127.80, 127.3, 126.8, 120.7, 115.0, 43.5, 42.7, 39.5. Data in accordance with the literature.[24]

Supplementary Note 4: Emission Spectra of Kessil LED lamps

The emission spectra of the Kessil LED lamps used in this study were taken from the Kessil website https://www.kessil.com/products/science_PR160L.php (Supplementary Figure 1).

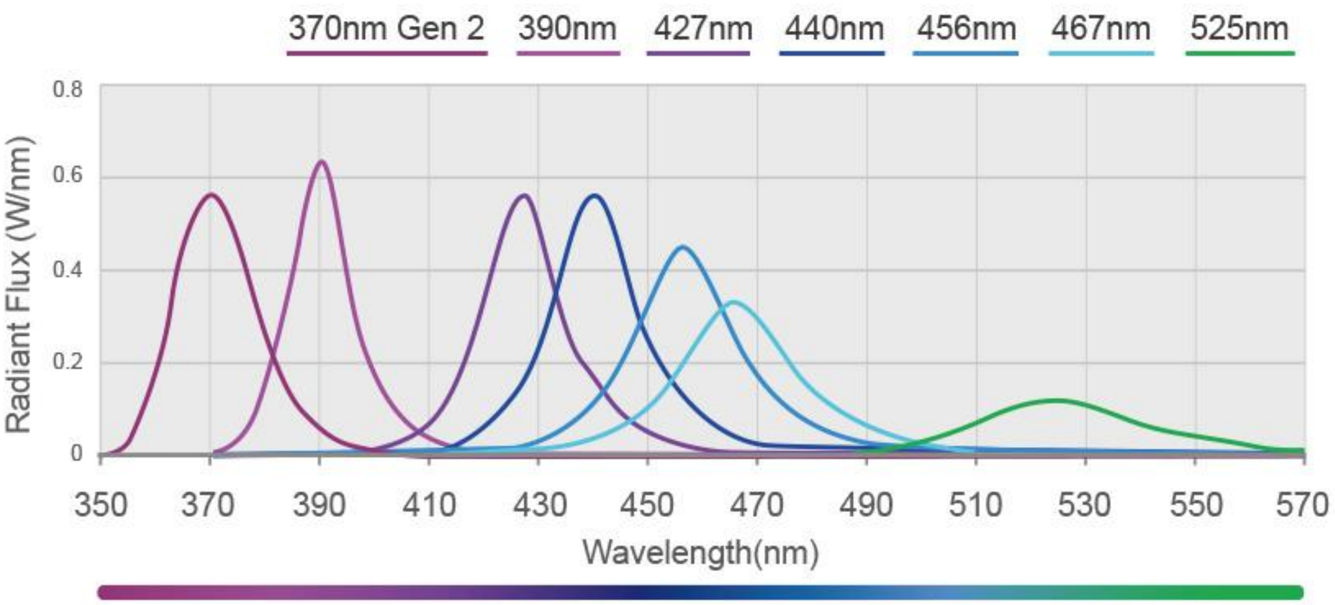

Supplementary Figure 1. Emission spectra of all LED lamp products by Kessil.

Supplementary Note 5: Pictures of Reaction Set-up

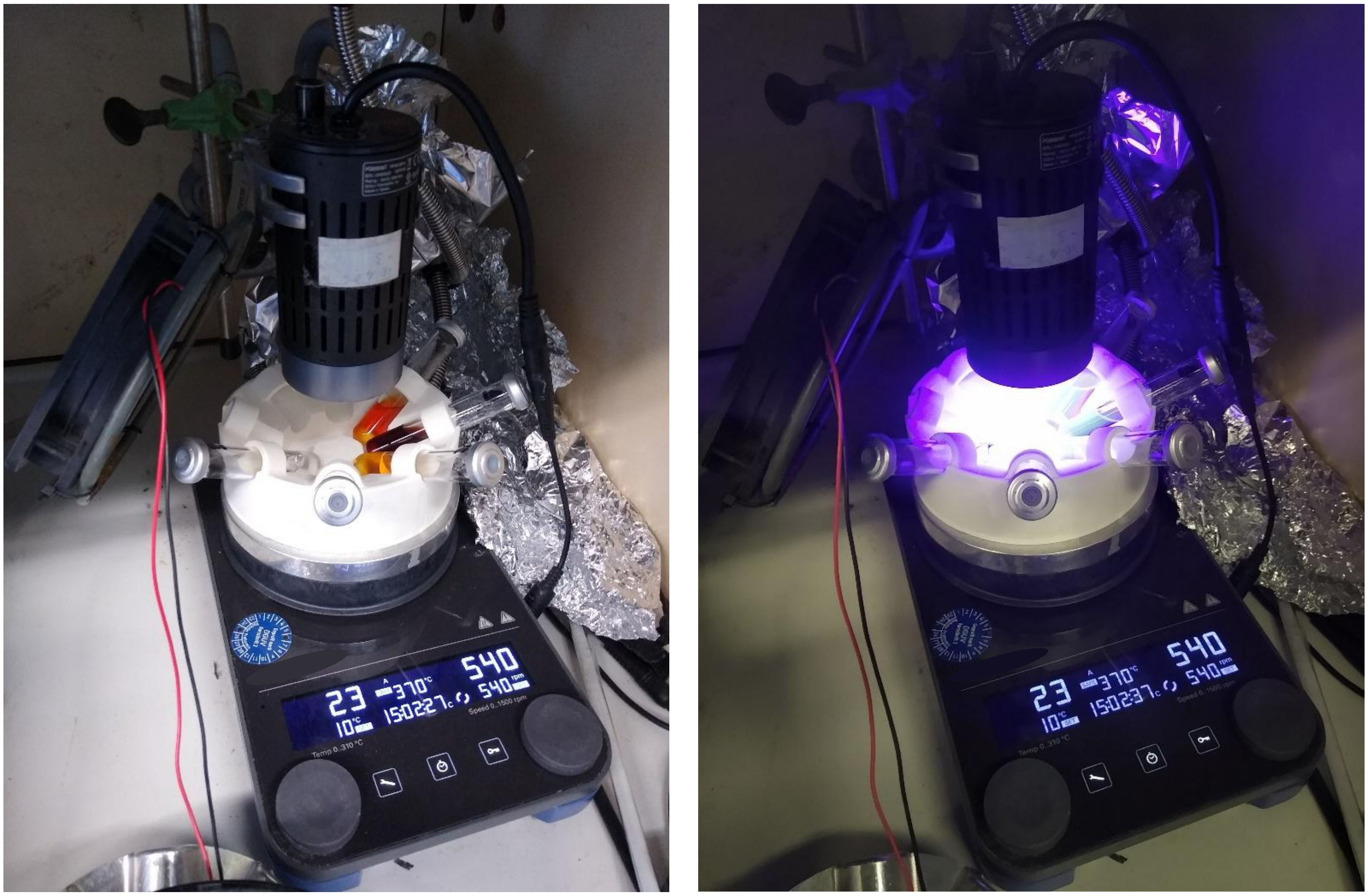

Supplementary Figure 2. Pictures of the reaction set-up used for photochemical reactions.

Supplementary Note 6: List of Nitroarenes Used in the Study

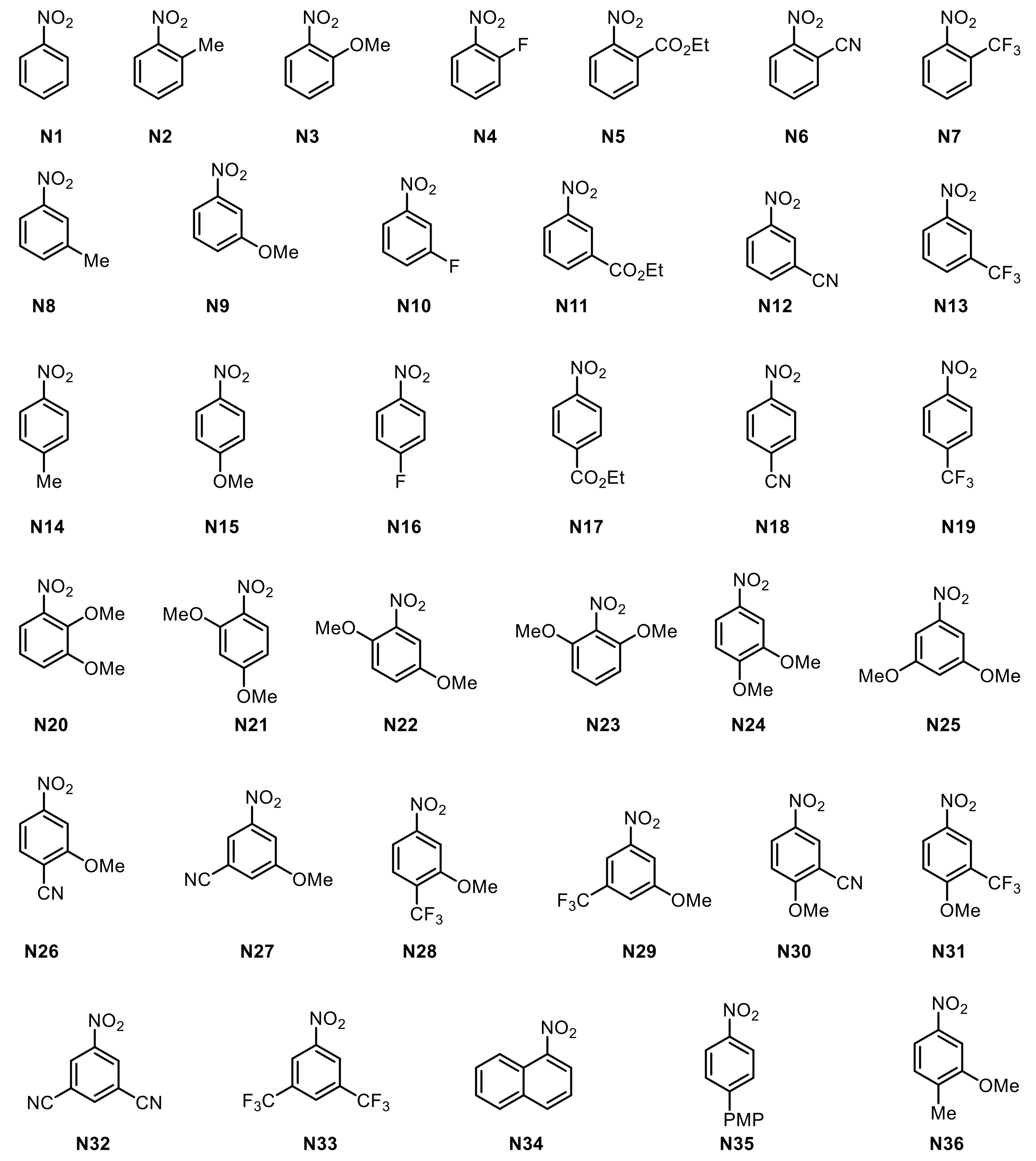

Supplementary Figure 3. List of all nitroarenes used in the present study.

Supplementary Note 7: Characterisation of Nitroarene Triplet Energies & NICS Values

Supplementary Table 1. Adiabatic triplet energies (E_T, in kcal mol^{-1}) for the $^3\pi\pi^*$ and $^3n\pi^*$ states of all nitroarenes, the energy difference between them (ΔE_{TT}, in kcal mol^{-1}), and the NICS$_{zz}$(1) values (in ppm) for each electronic state. GS: Ground State.

**Nitroarene**	**E_T**		**ΔE_{TT}**	**NICS$_{zz}$(1)**		
	**$^3\pi\pi^*$**	**$^3n\pi^*$**		**GS**	**$^3\pi\pi^*$**	**$^3n\pi^*$**
**N1**	77.5	64.8	12.7	-29.15	32.64	-27.18
**N2**	77.7	63.0	14.7	-28.07	23.91	-27.58
**N3**	73.3	62.2	11.1	-26.52	17.81	-26.64
**N4**	80.2	63.0	17.2	-27.73	20.01	-27.39
**N5**	75.5	62.4	13.1	-27.96	19.48	-27.23
**N6**	76.1	62.4	13.7	-28.36	31.35	-26.53
**N7**	77.2	62.3	14.9	-28.98	21.67	-27.77
**N8**	74.1	64.8	9.3	-27.90	31.41	-25.94
**N9**	63.6	64.6	–1.0	-27.07	19.94	-24.55
**N10**	75.6	64.3	11.3	-28.31	34.27	-25.76
**N11**	79.0	64.5	14.5	-27.67	34.24	-25.85
**N12**	80.3	64.1	16.2	-28.10	37.61	-25.97
**N13**	78.9	64.3	14.6	-28.41	32.79	-26.58
**N14**	74.1	65.3	8.8	-27.62	25.14	-25.90
**N15**	69.7	66.3	3.5	-25.67	15.00	-25.07
**N16**	76.8	65.2	11.6	-27.36	26.46	-27.16
**N17**	75.4	63.6	11.7	-27.88	36.34	-25.29
**N18**	74.9	63.2	11.7	-28.33	33.85	-25.43
**N19**	79.1	63.7	15.4	-28.84	37.13	-26.35
**N20**	67.2	61.1	6.1	-25.66	8.07	-25.73
**N21**	74.7	63.2	11.5	-22.64	14.43	-23.51
**N22**	57.0	62.0	-5.0	-25.36	12.28	-25.11
**N23**	76.8	62.8	14.0	-24.58	8.16	-24.39
**N24**	59.6	66.1	-6.5	-24.65	14.40	-23.20
**N25**	63.3	64.3	-1.0	-24.81	28.75	-21.74

**N26**	63.6	63.0	0.6	-25.46	24.94	-22.08
**N27**	65.9	63.9	2.0	-26.10	24.84	-23.46
**N28**	64.8	63.5	1.3	-26.16	23.22	-23.14
**N29**	65.3	64.0	1.3	-26.20	22.64	-23.56
**N30**	74.5	65.7	8.8	-23.88	23.79	-23.21
**N31**	72.7	65.9	6.8	-24.65	18.52	-23.99
**N32**	81.5	63.5	18.0	-26.98	42.10	-24.62
**N33**	79.8	63.7	16.1	-27.78	33.72	-25.63
**N34**	60.2	62.6	-2.4	-27.74	25.05	-27.10
**N35**	62.1	65.1	-3.0	-26.04	2.29	-13.56
**N36**	62.8	65.1	-2.3	-26.21	20.31	-23.97

Supplementary Note 8: Isomerization of α,β-Unsaturated Esters

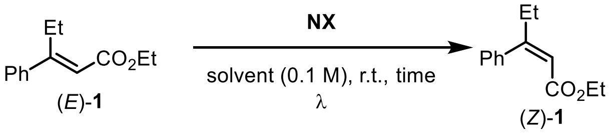

An oven-dried microwave tube containing a stir-bar was charged with ***E*-1** (20 mg, 0.1 mmol, 1.0 equiv.) and the nitroarene (10 mol%). The vial was capped with a Supelco aluminium crimp seal with septum (PTFE/butyl) and was evacuated and refilled with Ar (x 3). Dry and degassed solvent (1 mL, 0.1 M) was added, and the reaction mixture was stirred (>500 rpm) under irradiation for the specified time. A solution of 1,3-dinitrobenzene (0.5 mL of 0.2 M $CDCl_3$ solution) was added to the reaction mixture. An aliquot (100 µL) of the mixture was transferred to a NMR tube, diluted with 600 µL of $CDCl_3$ and analyzed using quantitative ^{1}H NMR spectroscopy to obtain ^{1}H NMR yield and the *E*/*Z* ratio.

Supplementary Table 2. Optimization table for the *E* to *Z* isomerization of ***E*-1**

**Entry**	**Nitroarene**	**Nitroarene loading (mol%)**	**Wavelength (nm)**	**Solvent**	**Time**	***E*-1 (%)**	**Z:E**
**1**	**N1**	10	390	CH_3CN	5 min	100	1:99
**2**	**N2**	10	390	CH_3CN	5 min	100	1:99
**3**	**N3**	10	390	CH_3CN	5 min	100	1:99
**4**	**N4**	10	390	CH_3CN	5 min	100	1:99
**5**	**N5**	10	390	CH_3CN	5 min	100	1:99
**6**	**N6**	10	390	CH_3CN	5 min	100	1:99
**7**	**N7**	10	390	CH_3CN	5 min	100	1:99
**8**	**N8**	10	390	CH_3CN	5 min	100	3:97
**9**	**N9**	10	390	CH_3CN	5 min	100	73:27
**10**	**N10**	10	390	CH_3CN	5 min	100	1:99
**11**	**N11**	10	390	CH_3CN	5 min	100	3:97
**12**	**N12**	10	390	CH_3CN	5 min	100	1:99
**13**	**N13**	10	390	CH_3CN	5 min	100	1:99
**14**	**N14**	10	390	CH_3CN	5 min	100	1:99
**15**	**N15**	10	390	CH_3CN	5 min	100	12:88
**16**	**N16**	10	390	CH_3CN	5 min	100	1:99
**17**	**N17**	10	390	CH_3CN	5 min	100	3:97
**18**	**N18**	10	390	CH_3CN	5 min	100	3:97
**19**	**N19**	10	390	CH_3CN	5 min	100	1:99
**20**	**N20**	10	390	CH_3CN	5 min	100	1:99

**21**	**N21**	10	390	CH_3CN	5 min	100	1:99
**22**	**N22**	10	390	CH_3CN	5 min	100	8:92
**23**	**N23**	10	390	CH_3CN	5 min	100	1:99
**24**	**N24**	10	390	CH_3CN	5 min	100	99:1
**25**	**N25**	10	390	CH_3CN	5 min	100	97:3
**26**	**N26**	10	390	CH_3CN	5 min	100	68:32
**27**	**N27**	10	390	CH_3CN	5 min	100	15:85
**28**	**N28**	10	390	CH_3CN	5 min	100	15:85
**29**	**N29**	10	390	CH_3CN	5 min	100	14:86
**30**	**N30**	10	390	CH_3CN	5 min	100	8:92
**31**	**N31**	10	390	CH_3CN	5 min	100	3:97
**32**	**N32**	10	390	CH_3CN	5 min	100	1:99
**33**	**N33**	10	390	CH_3CN	5 min	100	1:99
**34**	**N34**	10	390	CH_3CN	5 min	100	93:7
**35**	**N35**	10	390	CH_3CN	5 min	100	99:1
**36**	**N36**	10	390	CH_3CN	5 min	100	82:18
**37**	**N9**	10	390	CH_3CN	6 h	100	99:1
**38**	**N23**	10	390	CH_3CN	6 h	100	31:69
**39**	**N24**	10	390	CH_3CN	6 h	100	99:1
**40**	**N25**	10	390	CH_3CN	6 h	100	99:1
**41**	**N33**	10	390	CH_3CN	6 h	83	37:63
**42**	**N24**	10	390	CH_2Cl_2	16 h	100	99:1
**43**	**N24**	10	390	THF	16 h	95	96:4
**44**	**N24**	10	390	Et_2O	16 h	95	95:5
**45**	**N24**	10	390	EtOH	16 h	99	98:2
**46**	**N24**	10	390	EtOAc	16 h	100	98:2
**47**	**N24**	10	390	acetone	16 h	100	99:1
**48**	**N24**	10	390	$PhCF_3$	16 h	94	99:1
**49**	**N24**	10	390	DMSO	16 h	94	99:1
**50**	**N24**	10	390	DMF	16 h	85	98:2
**51**	**N24**	10	390	DMA	16 h	87	95:5
**52**	**N24**	10	390	CH_3CN	1 h	100	99:1
**53**	**N24**	5	390	CH_3CN	1 h	100	99:1
**54**	**N24**	2	390	CH_3CN	1 h	100	97:3

**55**	**N24**	5	390	CH_3CN	1 min	100	22:78
**56**	**N24**	5	390	CH_3CN	2 min	100	50:50
**57**	**N24**	5	390	CH_3CN	3 min	100	63:37
**58**	**N24**	5	390	CH_3CN	5 min	100	99:1
**59**	**N24**	5	390	CH_3CN	15 min	100	99:1
**60**	**N24**	5	390	CH_3CN	30 min	100	99:1
**61**	**N24**	5	390	CH_3CN	45 min	100	99:1
**62**	**N24**	5	427	CH_3CN	1 h	100	99:1
**63**	**N24**	5	440	CH_3CN	1 h	100	21:79
**64**	**-**	-	390	CH_3CN	16 h	100	15:85
**65**	**N24**	5	-	CH_3CN	16 h	100	1:99
**66**	**N24**	5	390	CH_3CN (air)	16 h	96	99:1

Supplementary Note 9: Mechanistic Investigations

Absorption Profiles of N1-36

To test whether the nitroarenes evaluated in the screening were absorbing light from a Kessil PR 160 390 nm LED, the absorption profile of **N1-36** was taken. Samples used for UV-Vis measurements were prepared by weighing the nitroarene (0.0625 mmol) and adding to a volumetric flask (25 mL) which was made to volume using HPLC grade CH_3CN. A 0.5 mL aliquot was taken and transferred to a second volumetric flask (10 mL) and made to volume using HPLC grade CH_3CN. This solution (0.125 mM) was used to measure absorption profiles of nitroarenes **N1-36** (Supplementary Figure 4).

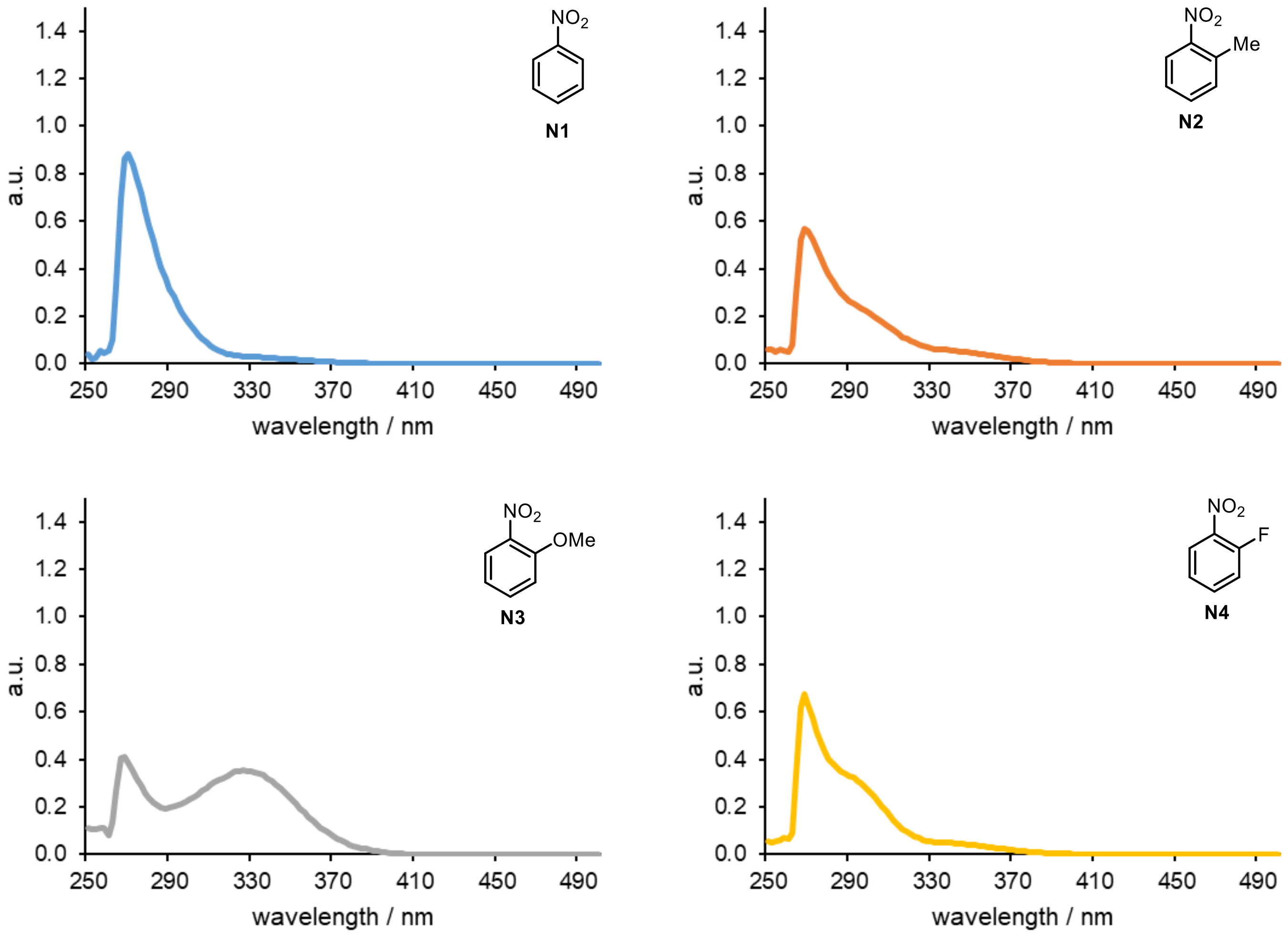

Supplementary Figure 4. Steady-state absorption profiles of nitroarenes **N1–36**.

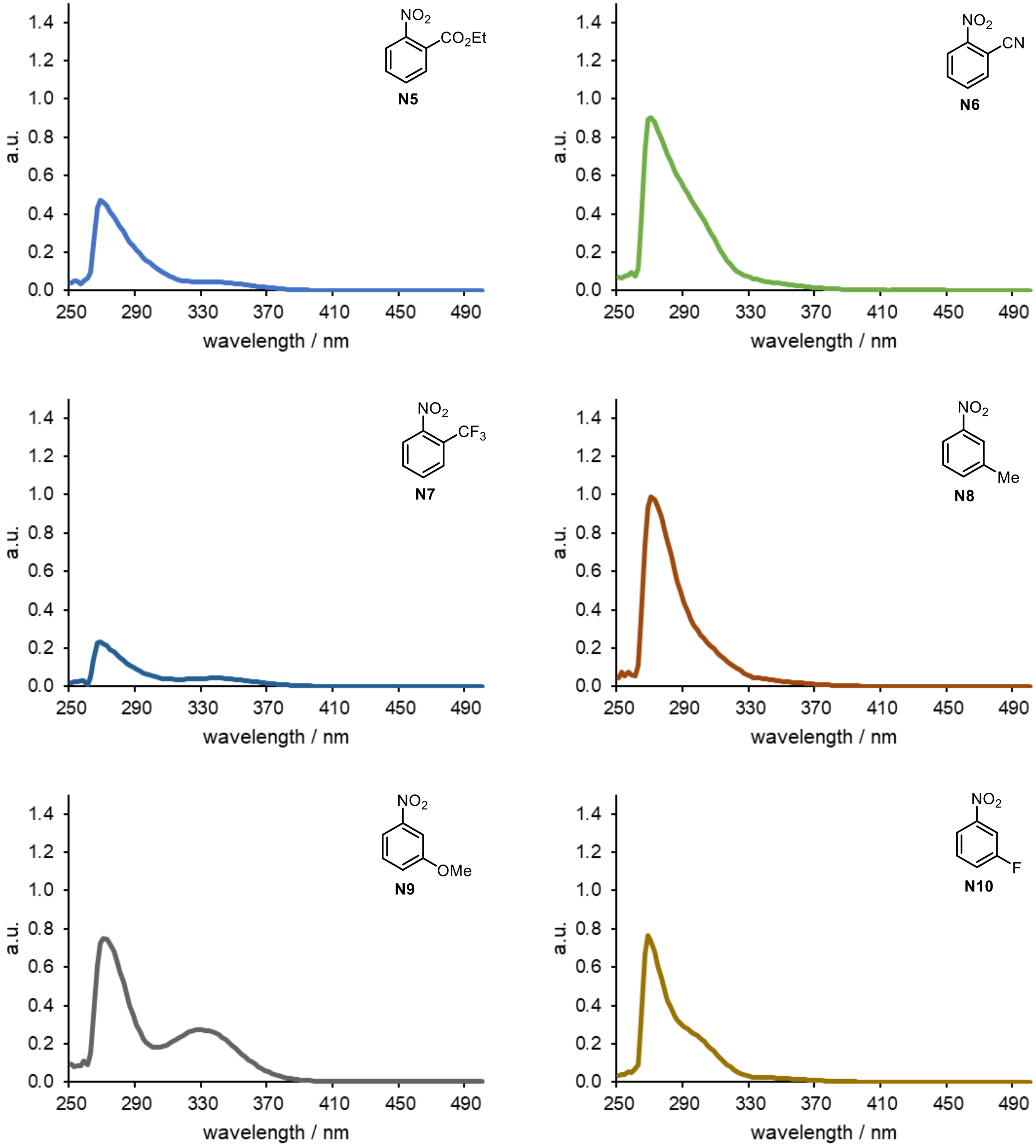

Supplementary Figure 4. continued.

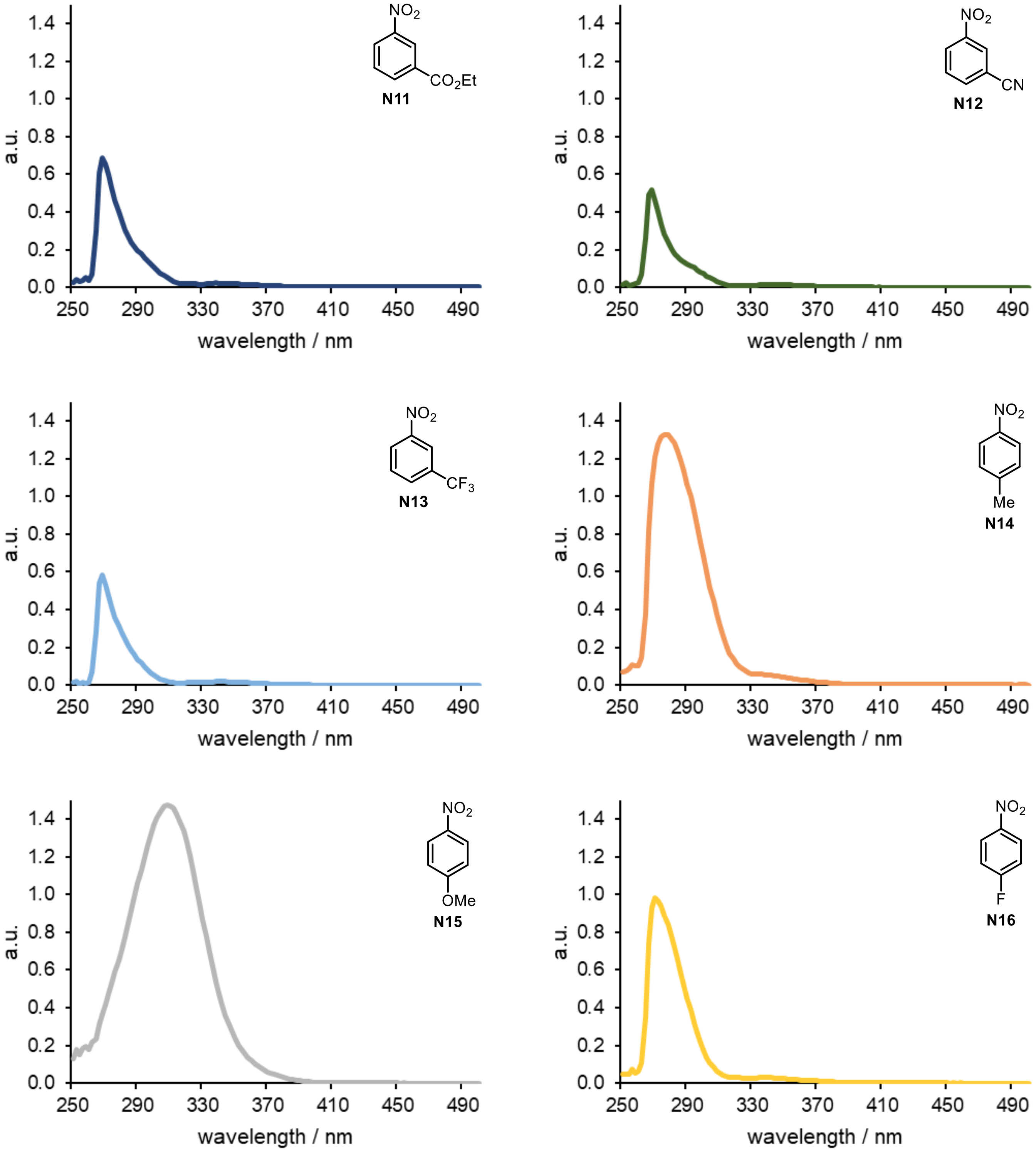

Supplementary Figure 4. continued.

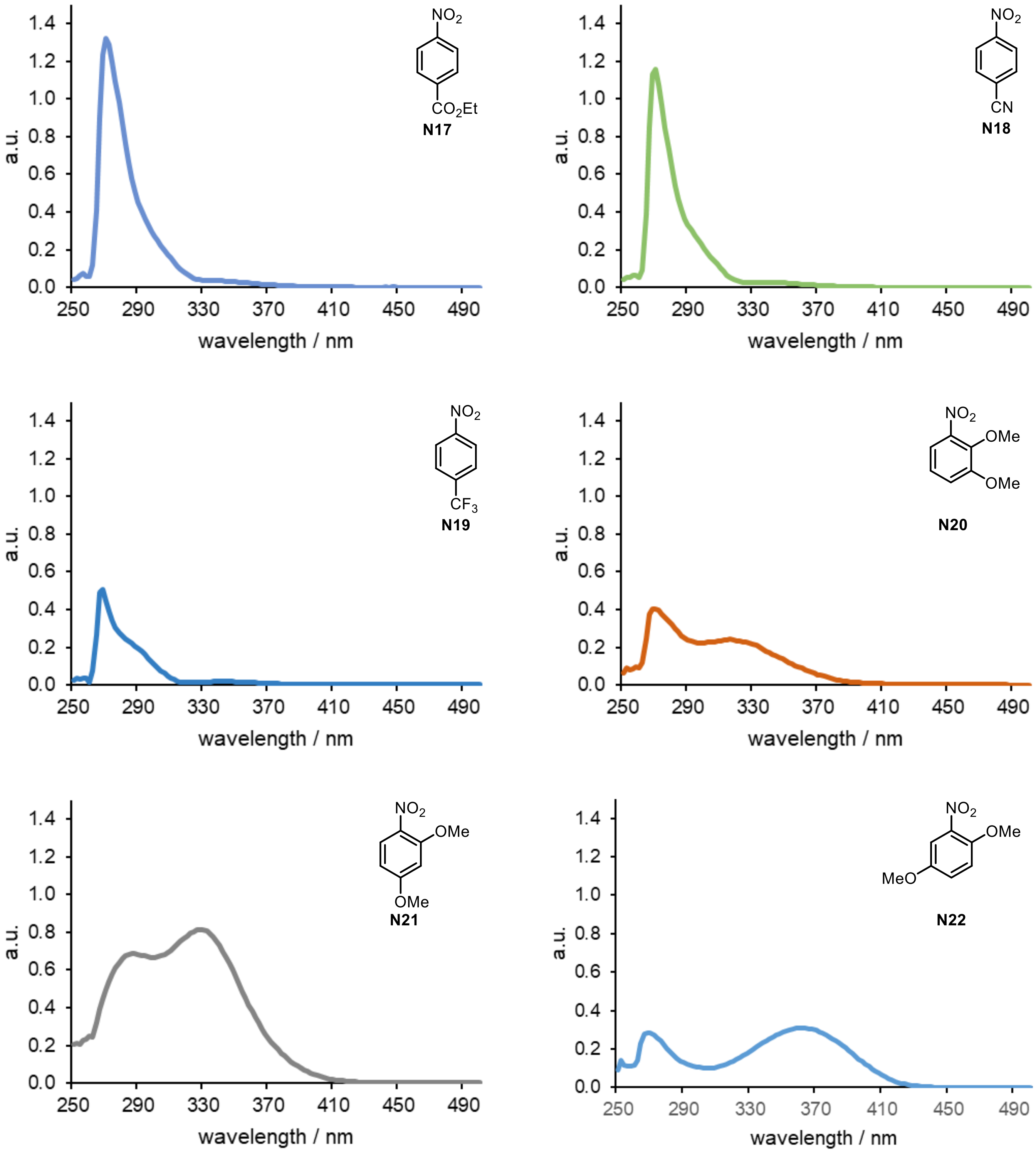

Supplementary Figure 4. continued.

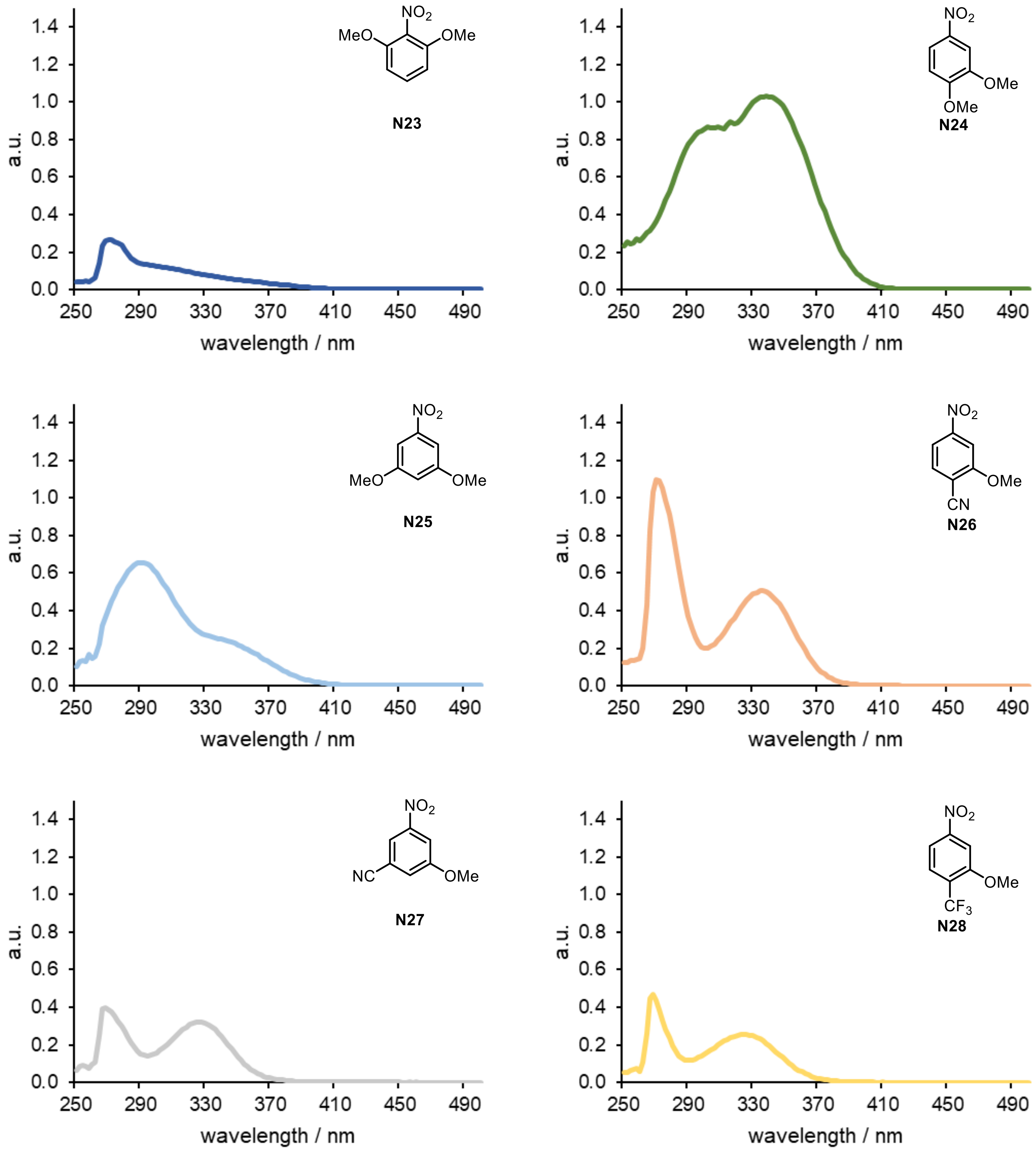

Supplementary Figure 4. continued.

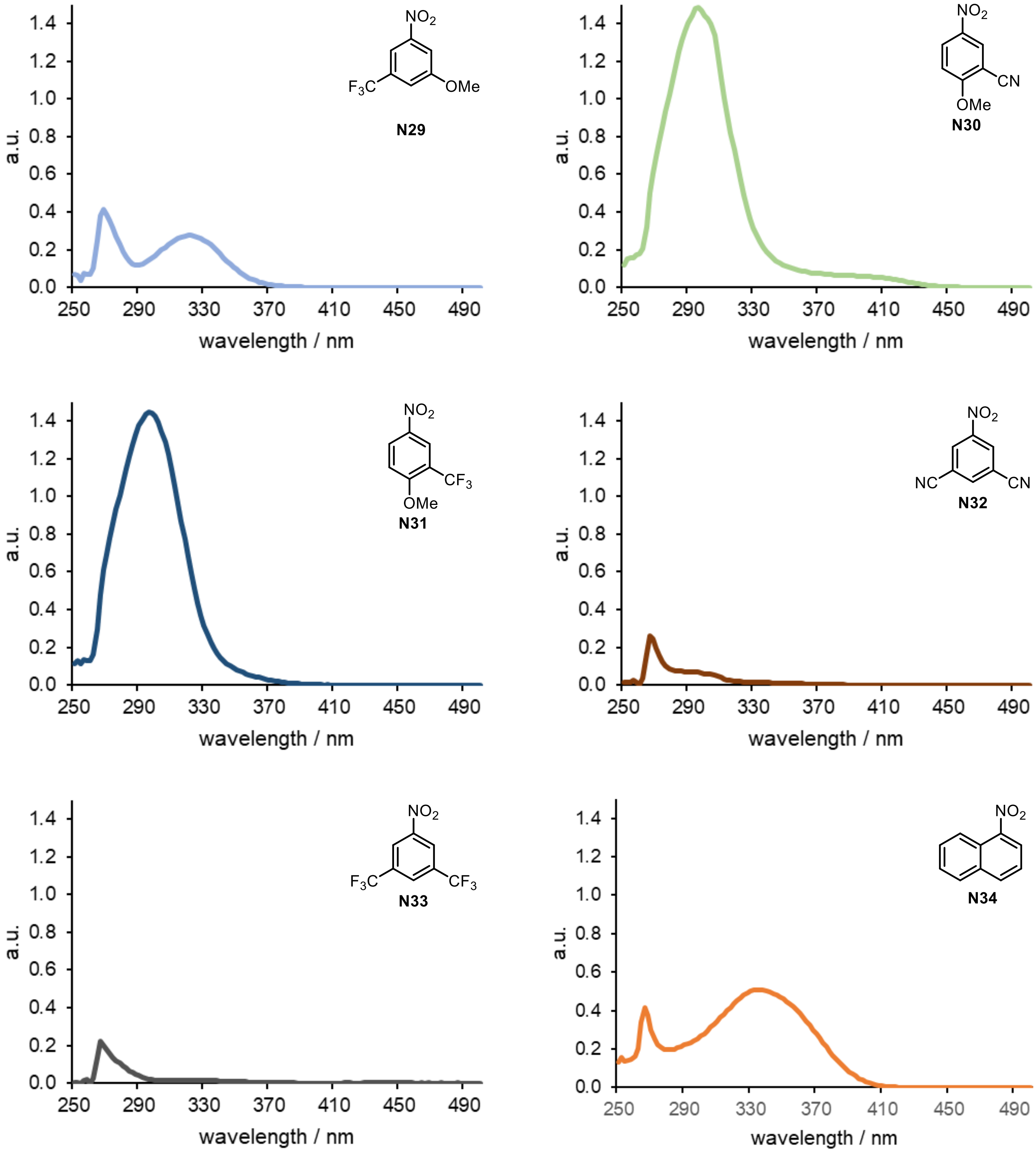

Supplementary Figure 4. continued.

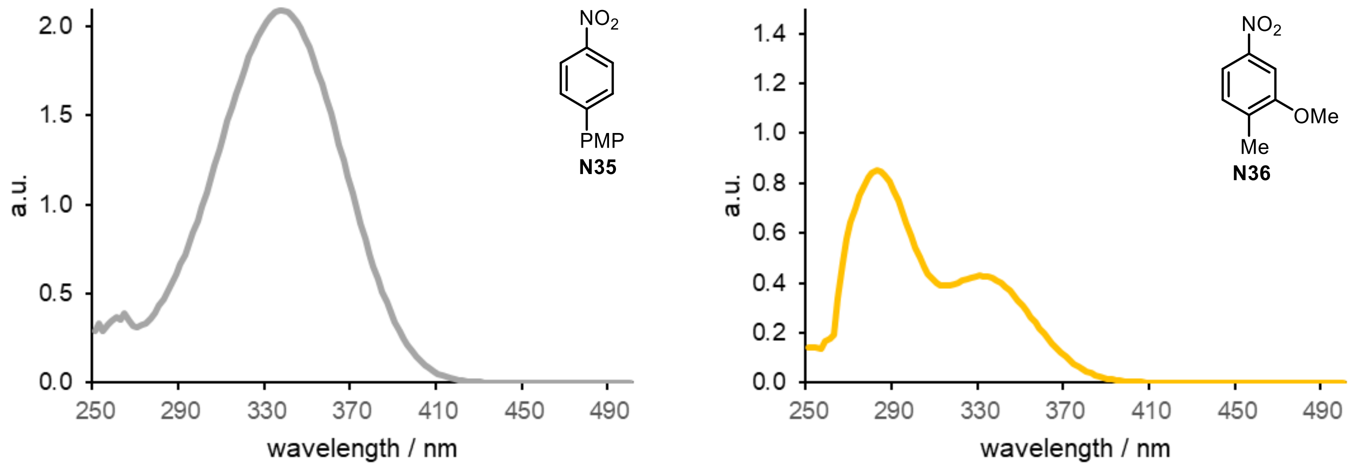

Supplementary Figure 4. continued.

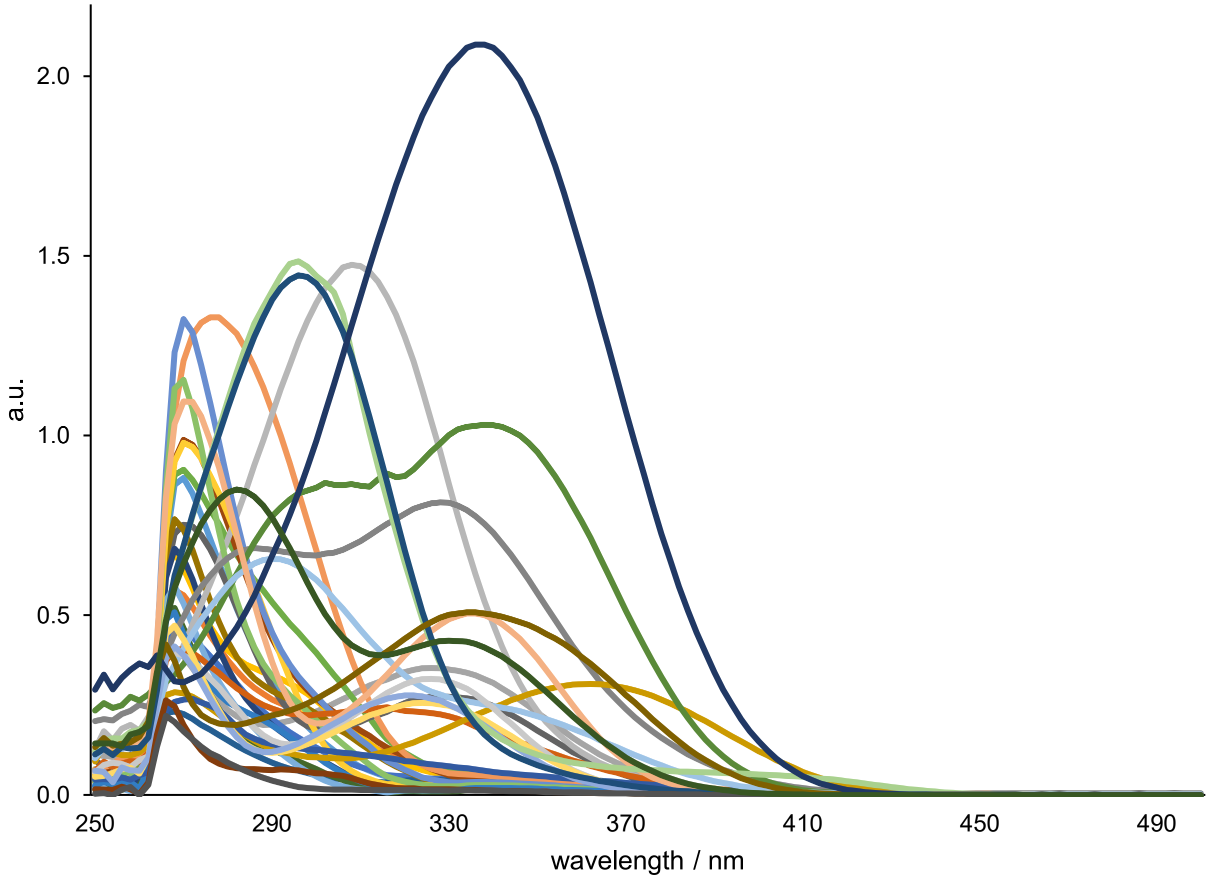

Supplementary Figure 5. Absorbance spectra in CH_3CN of nitroarenes **N1-N36** overlapped.

Detection of Ground State EDA Complexes

To rule out the formation of a ground state electron donor-acceptor (EDA) complex,[25] mixtures of the optimal nitroarene and substrate were analyzed by UV-Vis absorption spectroscopy to determine if any change in the visible absorption profile occurs. From the data collected, it is not evident that an EDA complex is formed under reaction conditions.

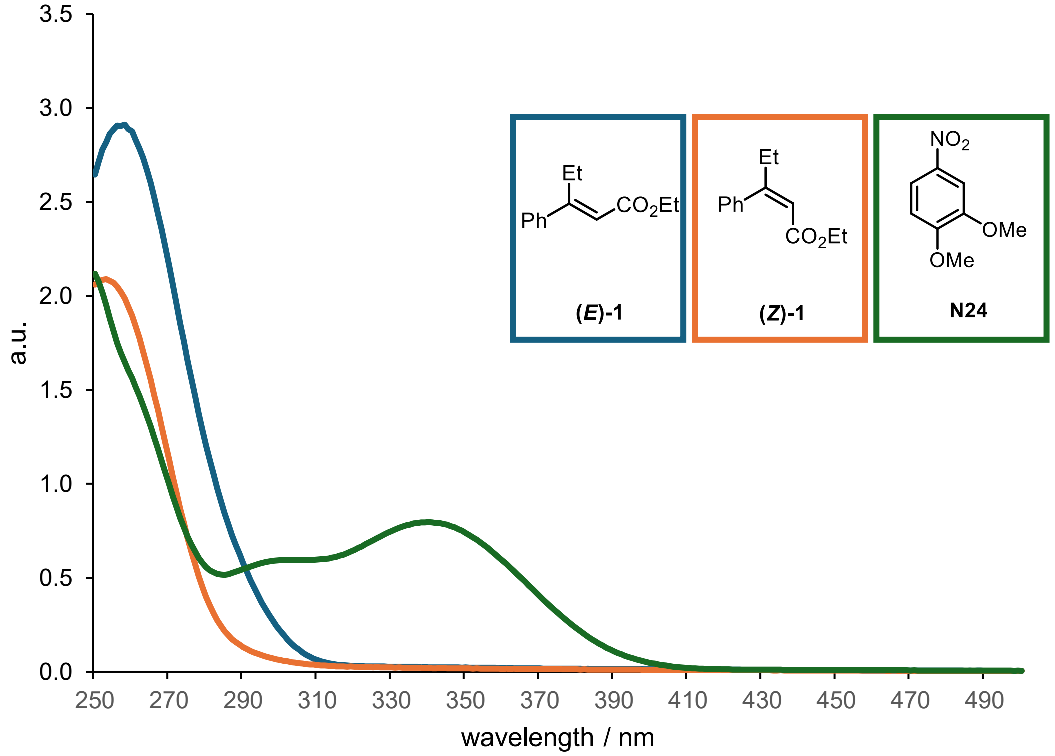

Supplementary Figure 6. Absorption spectrum of ***E*-1**, **Z-1**, and **N24** in CH_3CN (0.1 mM).

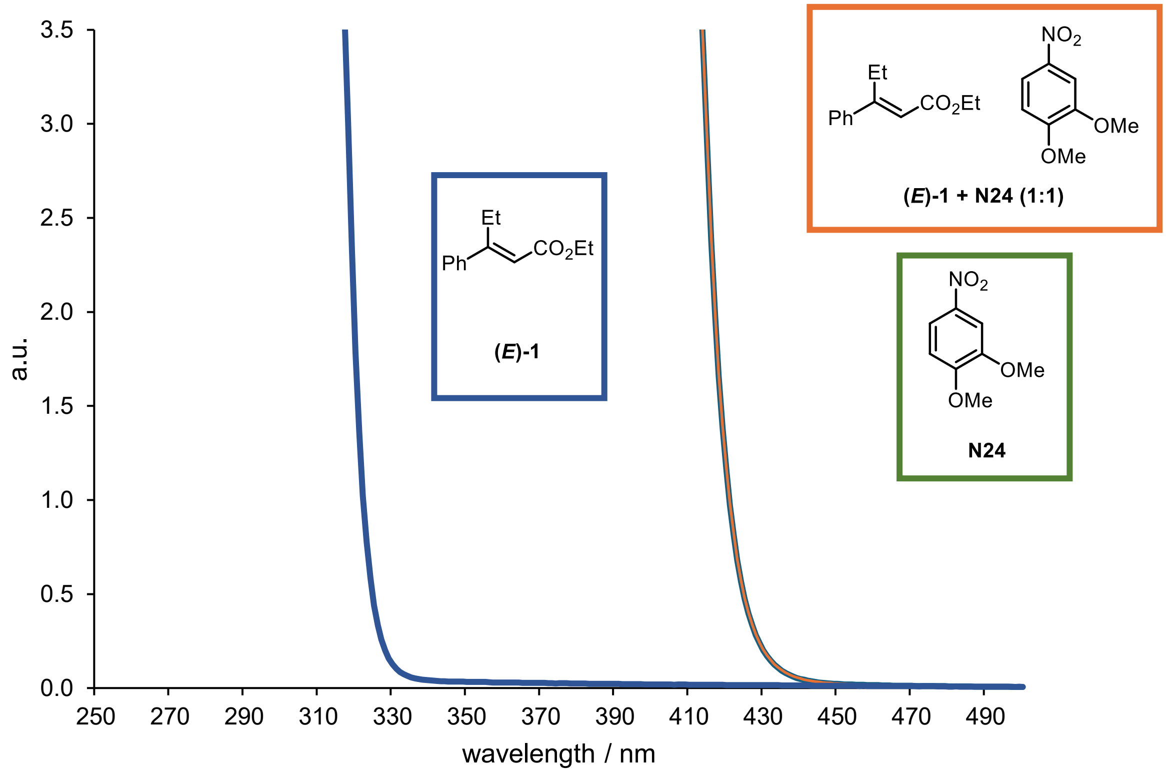

Supplementary Figure 7. Absorption spectrum of ***E*-1**, **N24** and 1:1 mixture in CH_3CN (0.1 M, reaction concentration).

Calculation of the Activation Barriers for Energy Transfer

Due to the difficulties in optimizing the transition state for EnT reactions, we adopted the procedure described recently by Solé-Daura and Maseras.[26] This procedure translates Marcus theory for SET into EnT, see Supplementary Figure 8 below. Therein, the blue curve represents the potential energy surface of the reagents **R**, i.e. the photoexcited nitroarene and the substrate. The green curve, in contrast, represents the potential energy surface of the products **P**, i.e. the ground-state nitroarene and the substrate in its triplet state.

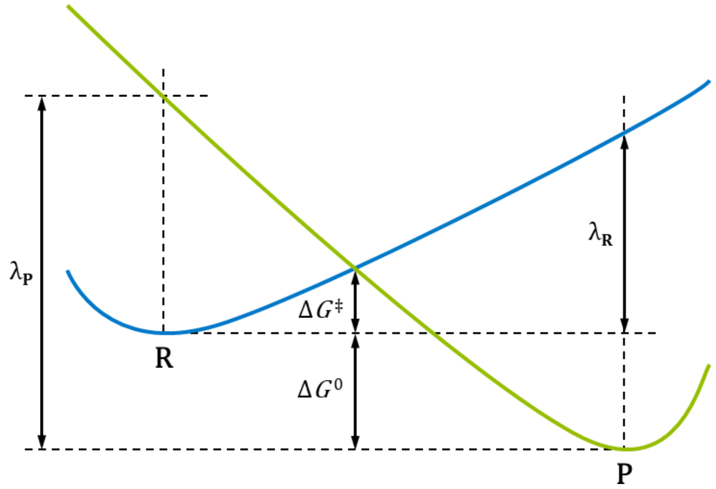

Supplementary Figure 8. Representation of the Marcus theory for energy transfer as proposed by the Maseras group.

In this framework, the activation energy $\Delta G^{\ddagger}$ can be expressed in terms of the reaction energy ΔG^0, the reorganization energy of the reagents $\lambda_{\mathbf{R}}$, and the reorganization energy of the products $\lambda_{\mathbf{P}}$ (1):[27]

$$\Delta G^{\ddagger} = \lambda_{\mathbf{R}} \left(\frac{-\lambda_{\mathbf{P}} + \sqrt{\lambda_{\mathbf{R}}\lambda_{\mathbf{P}} + (\lambda_{\mathbf{R}} - \lambda_{\mathbf{P}})\Delta G^0}}{\lambda_{\mathbf{R}} - \lambda_{\mathbf{P}}} \right)^2 \quad (1)$$

The reorganization energies can be readily calculated by taking single-point energy calculations of a given spin over the optimized geometry of the opposite spin. Moreover, this model assumes that the reorganization energies are additive, i.e. the total reorganization energy (of the reagents or products) is the sum of all the individual ones. Supplementary Table 3 contains the Gibbs free energies and reorganization energies necessary to replicate the activation energies shown in Figure 2c.

Supplementary Table 3. Gibbs free energies and reorganization energies (in kcal mol^{-1}) for the individual transformations during energy transfer (top half) and all the energy transfer reactions studied in Figure 2c (bottom half). The ethyl groups in **1** were replaced for methyl for simplicity. GS: Ground state.

**Transformations**	**ΔG^0**	**λ_R**	**λ_P**	**ΔG‡**
E-**2** → ^{3}**2**	48.4	51.6	28.6	---
Z-**2** → ^{3}**2**	46.8	49.6	46.4	---
**N24** (^{3}π,π*) → **N24** (GS)	-56.3	13.4	11.0	---
**N23** (^{3}n,π*) → **N23** (GS)	-58.5	49.3	39.0	---
**N33** (^{3}n,π*) → **N33** (GS)	-59.4	52.2	28.4	---
***E*-2** + **N24** (^{3}π,π*) → ^{3}**2** + **N24** (GS)	-7.9	65.0	39.5	8.3
***Z*-2** + **N24** (^{3}π,π*) → ^{3}**2** + **N24** (GS)	-9.5	62.9	57.4	10.5
***E*-2** + **N23** (^{3}n,π*) → ^{3}**2** + **N23** (GS)	-10.1	100.9	67.6	15.2
***Z*-2** + **N23** (^{3}n,π*) → ^{3}**2** + **N23**(GS)	-11.7	98.9	85.4	17.3
***E*-2** + **N33** (^{3}n,π*) → ^{3}**2** + **N33** (GS)	-11.0	103.9	56.9	12.9
***Z*-2** + **N33** (^{3}n,π*) → ^{3}**2** + **N33** (GS)	-12.6	101.8	74.8	15.4

The differences in the activation energies for the *E*-to-*Z* photoisomerization using different nitroarenes can be understood in terms of λ_R and λ_P. These reorganizations energies refer to the differences in geometry differences and solvent interactions between the initial and final states. For typical ^{3}π,π* nitroarenes, such as **N24**, there are negligible variations in the geometry due to the planarity of this triplet state, which are then translated to low values of λ_R and λ_P and thus to a lower activation barrier. In contrast, typical ^{3}n,π* nitroarenes like **N33** involve the pyramidalization of the nitro group in the triplet state. This crucial change in the geometry raises both λ_R and λ_P and leads to larger barriers.

Laser Flash Photolysis and Stern-Volmer experiments

Excited State Lifetimes

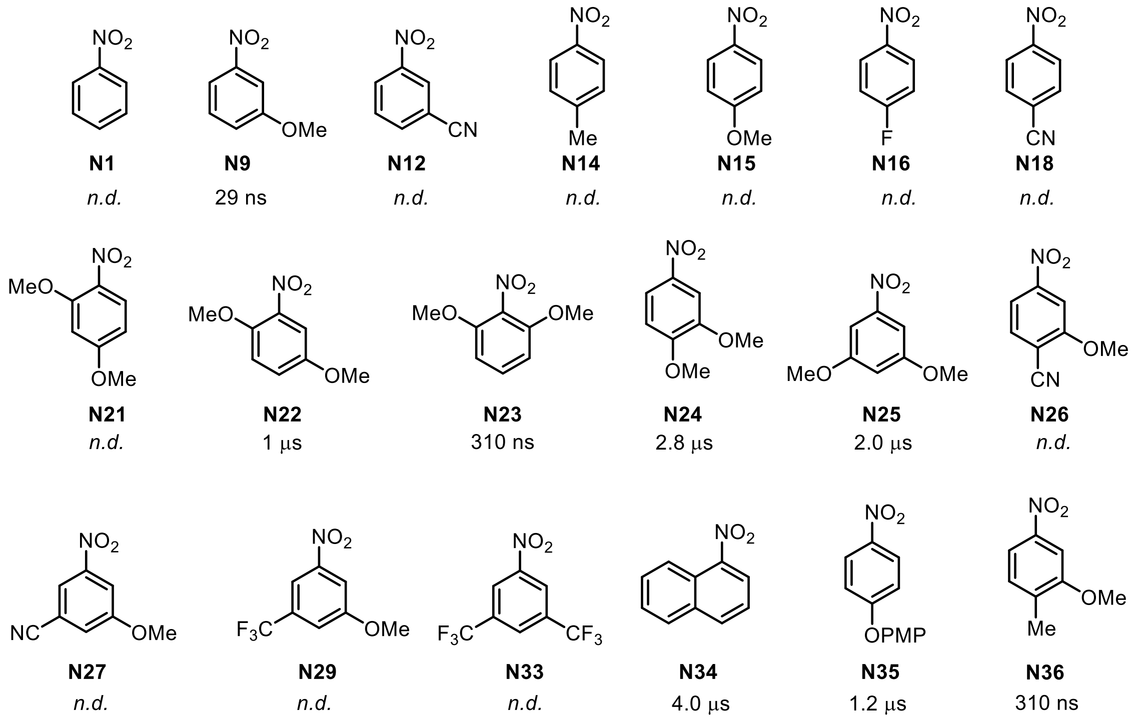

Supplementary Figure 9. Excited state lifetimes were determined by fitting an exponential decay function to the kinetic trace at the corresponding maximum TA peak (see below in section *Transient Absorption Spectra, Decay Traces, and Stern−Volmer Experiments*). n.d. = not detected.

To evaluate potential factors that govern the efficiency of the EnT process, excited state lifetimes were measured with TA spectroscopy. No transient absorption was observed for most nitroarenes with high values of ΔE_T (see Supplementary Note 7) due to short lifetimes of the corresponding $^3n\pi^*$ states. Nitroarenes with lower values of ΔE_T have longer lifetimes, enabling their use in EnT catalysis.

Triplet-triplet Energy Transfer Constants and Stern−Volmer Experiments

The triplet-triplet energy transfer constants, K_q, were determined from Stern−Volmer experiments.[13] The lifetime τ of triplet-excited 3,4-dimethoxynitrobenzene was measured with varying concentrations c_q of the quencher and fitted according to the modified Stern−Volmer equation (2):

$$\frac{\tau_0}{\tau} = K_q \cdot \tau_0 \cdot c_q + 1 \tag{2}$$

Typical unquenched triplet lifetimes of nitroarenes are on the order of 0.1–5.0 μs under our conditions, which have been optimized for obtaining high signal-to-noise ratios and highly sensitive TA measurements. Despite the slight self-quenching effects shortening the natural lifetime that are commonly observed for nitroarenes under these conditions, they do not affect the determinations of the quenching rate constants. This is because we used the apparent τ_0 value corresponding to the conditions on the day of the actual Stern−Volmer analysis. The (quenched) lifetimes under the conditions used for key experiments are too short for any self-quenching side effects.

Transient Absorption Spectra, Decay Traces, and Stern−Volmer Experiments

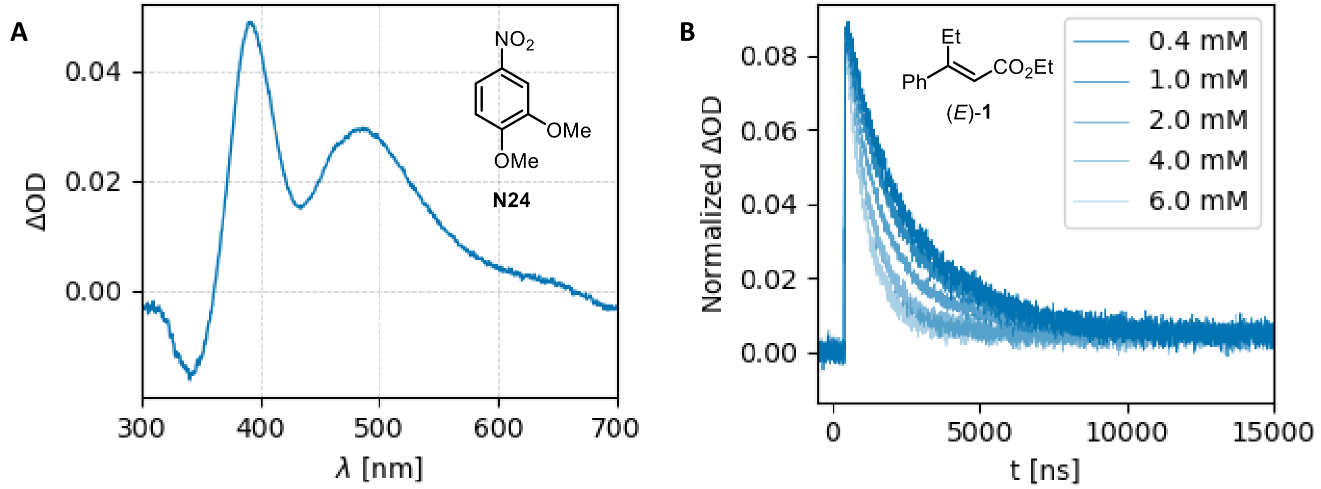

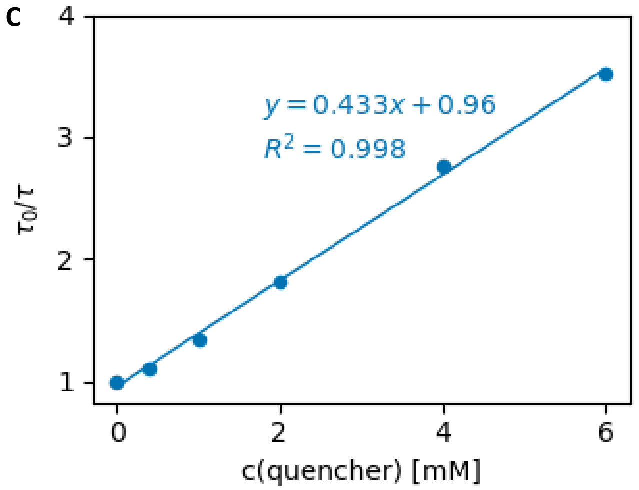

D

Q [mM]	τ [ns]	τ_0/τ
0.0	2590	1.000
0.4	2348	1.103
1.0	1925	1.346
2.0	1429	1.813
4.0	940	2.757
6.0	735	3.524
K_q	$1.7\ 10^8$ M^{-1} s^{-1}	

Supplementary Figure 10. **A**. Transient absorption spectrum of **N24** in deaerated CH_2Cl_2 (λ_{exc} = 355 nm). **B**. Normalized transient decay traces for **N24** in deaerated CH_2Cl_2 (λ_{exc} = 355 nm, 20 µM) monitored at 390 nm upon addition of increasing amounts of ***E*-1**. In all cases, the decays were fitted to a monoexponential function. **C**. Corresponding Stern−Volmer plot. **D**. Apparent lifetimes τ at increasing concentrations of quencher and calculated K_q.

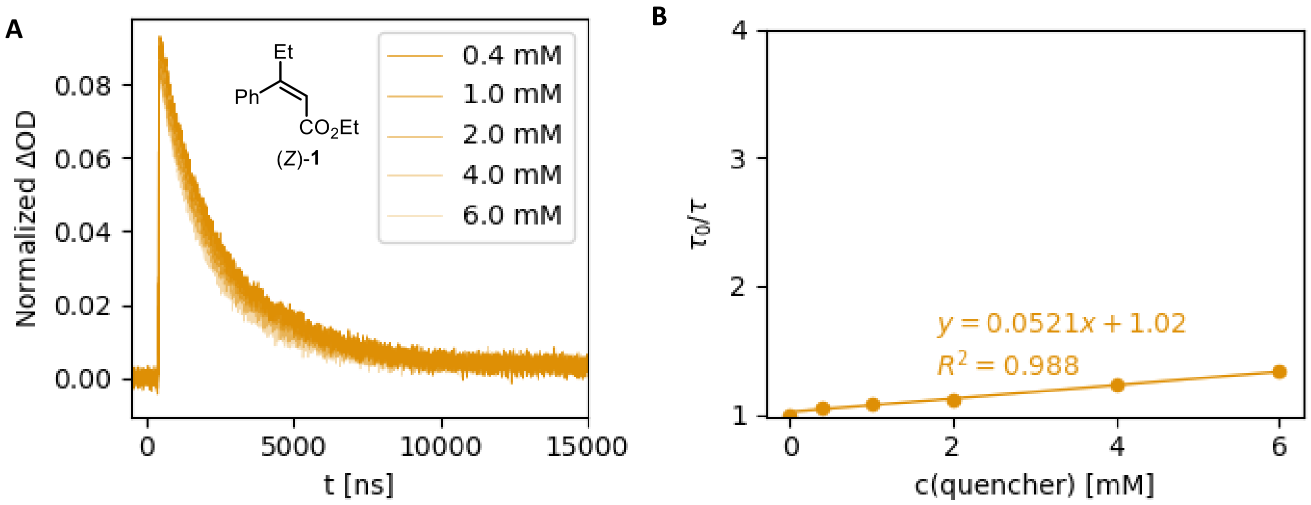

C

Q [mM]	τ [ns]	τ_0/τ
0.0	2354	1.000
0.4	2228	1.057
1.0	2165	1.087
2.0	2107	1.117
4.0	1916	1.229
6.0	1767	1.333
K_q	$0.22\ 10^8\ M^{-1}\ s^{-1}$	

Supplementary Figure 11. **A**. Normalized transient decay traces for **N24** in deaerated CH_2Cl_2 (λ_{exc} = 355 nm, 20 µM) monitored at 390 nm upon addition of increasing amounts of ***Z*-1**. In all cases, the decays were fitted to a monoexponential function. **B.** Corresponding Stern−Volmer plot. **C.** Apparent lifetimes τ at increasing concentrations of quencher and calculated K_q.

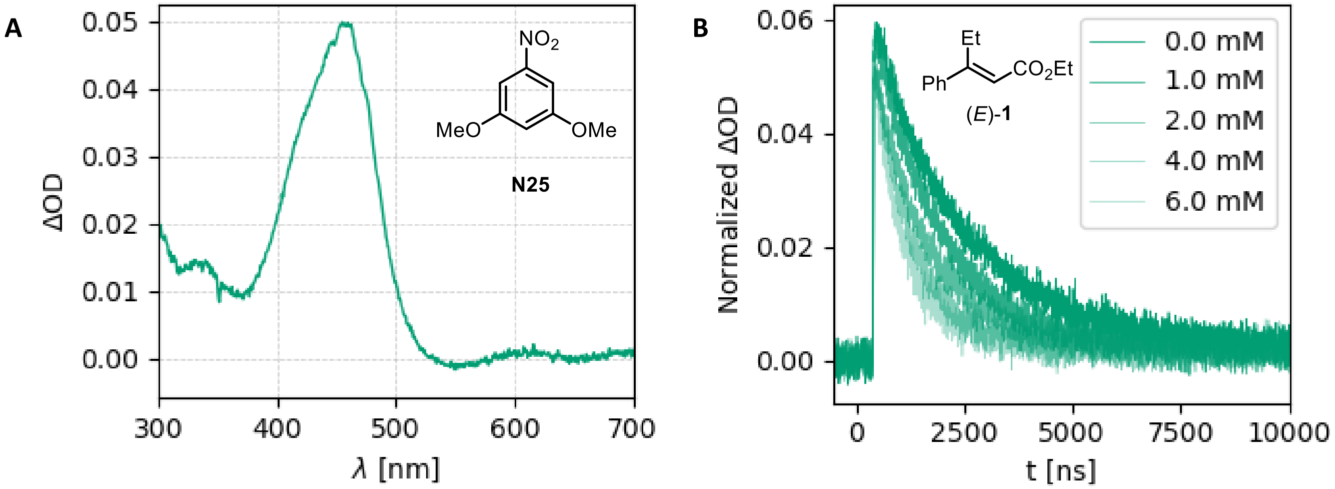

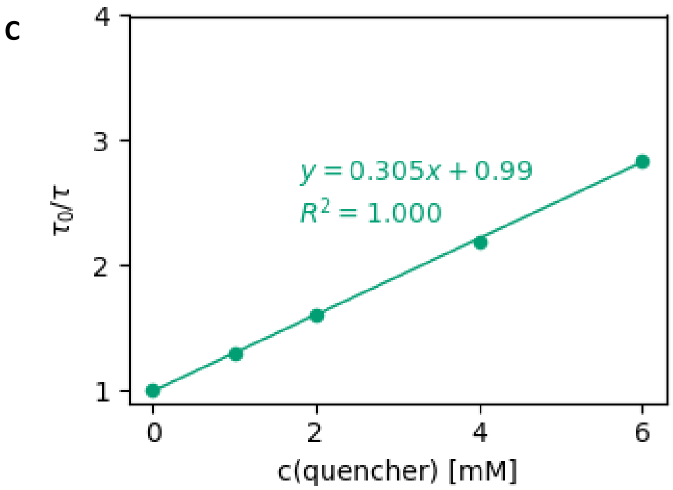

D

Q [mM]	τ [ns]	τ_0/τ
0.0	309	1.000
1.0	257	1.202
2.0	241	1.282
4.0	193	1.601
6.0	161	1.919
K_q	4.9 10^8 M^{-1} s^{-1}	

Supplementary Figure 12. **A**. Transient absorption spectrum of **N25** in deaerated CH_2Cl_2 (λ_{exc} = 355 nm). **B**. Normalized transient decay traces for **N25** in deaerated CH_2Cl_2 (λ_{exc} = 355 nm, 20 µM) monitored at 456 nm upon addition of increasing amounts of ***E*-1**. In all cases, the decays were fitted to a monoexponential function. **C**. Corresponding Stern−Volmer plot. **D**. Apparent lifetimes τ at increasing concentrations of quencher and calculated K_q.

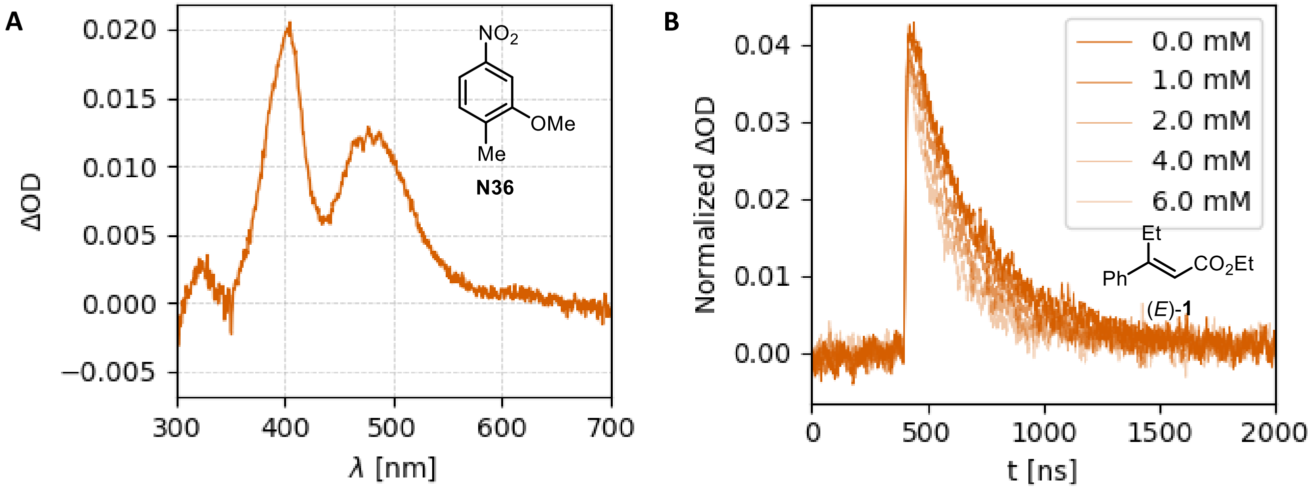

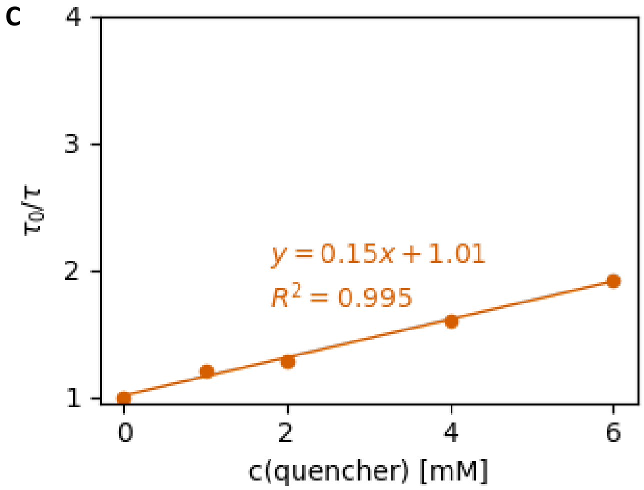

D

Q [mM]	τ [ns]	τ_0/τ
0.0	2024	1.000
1.0	1559	1.298
2.0	1255	1.613
4.0	924	2.190
6.0	712	2.843
K_q	$1.5\ 10^8\ M^{-1}\ s^{-1}$	

Supplementary Figure 13. **A**. Transient absorption spectrum of **N36** in deaerated CH_2Cl_2 (λ_{exc} = 355 nm). **B**. Normalized transient decay traces for **N36** in deaerated CH_2Cl_2 (λ_{exc} = 355 nm, 20 µM) monitored at 477 nm upon addition of increasing amounts of ***E*-1**. In all cases, the decays were fitted to a monoexponential function. **C**. Corresponding Stern−Volmer plot. **D**. Apparent lifetimes τ at increasing concentrations of quencher and calculated K_q.

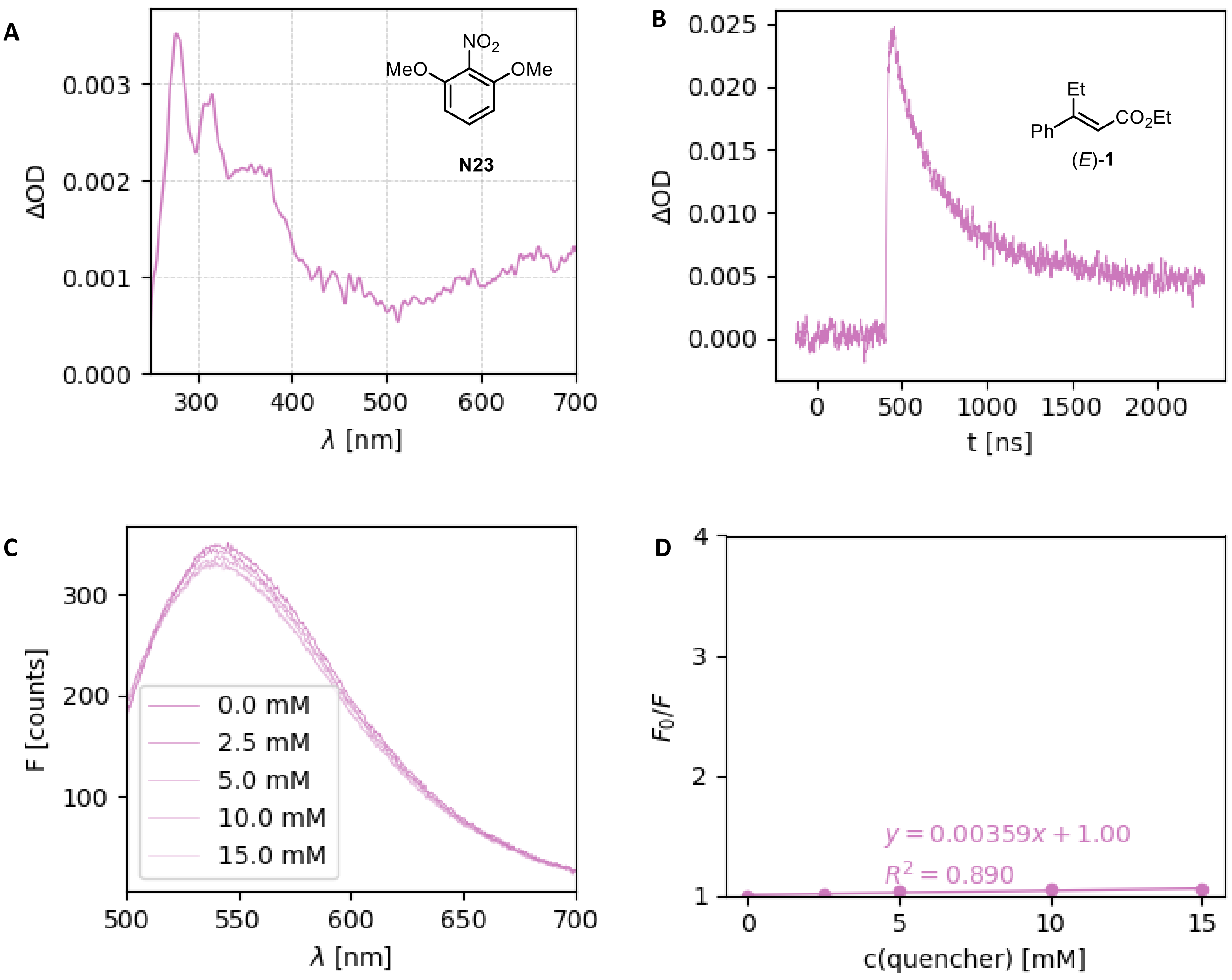

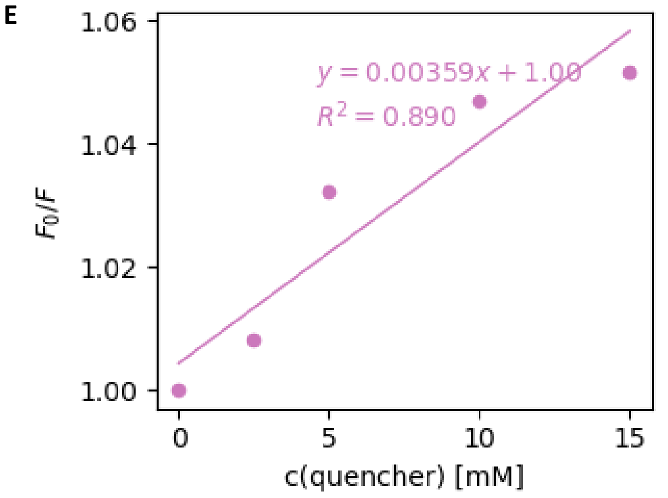

F

Q [mM]	F	F_0/F
0.0	347	1.000
2.5	344	1.008
5.0	336	1.032
10.0	331	1.047
15.0	330	1.052
K_q	$0.12\ 10^8\ M^{-1}\ s^{-1}$	

Supplementary Figure 14. **A**. Transient absorption spectrum of **N23** in deaerated CH_2Cl_2 (λ_{exc} = 266 nm). **B.** Transient decay trace for **N23** in deaerated CH_2Cl_2 (λ_{exc} = 266 nm) monitored at 350 nm. **C.** Emission spectra for **N23** in deaerated

CH_2Cl_2 (λ_{exc} = 400 nm, 50 µM) at increasing amounts of ***E*-1**. **D**. Corresponding Stern−Volmer plot. **E.** Stern-Volmer plot with rescaled y-axis. **F**. Emission intensity *F* at increasing concentrations of quencher and calculated K_q.

Due to inefficient energy transfer between **N23** and ***E*-1**, only weak emission quenching was observed (Supplementary Figure 14, panel **C**). Therefore, noise accounts for a higher proportion of variance in measured emission intensity, resulting in $R^2 = 0.890$. Slope determination is nonetheless statistically significant, with a p-value of 0.016 and an associated standard error of 20%, yielding $K_q = (1.2 \pm 0.2)\ 10^7\ M^{-1}\ s^{-1}$.

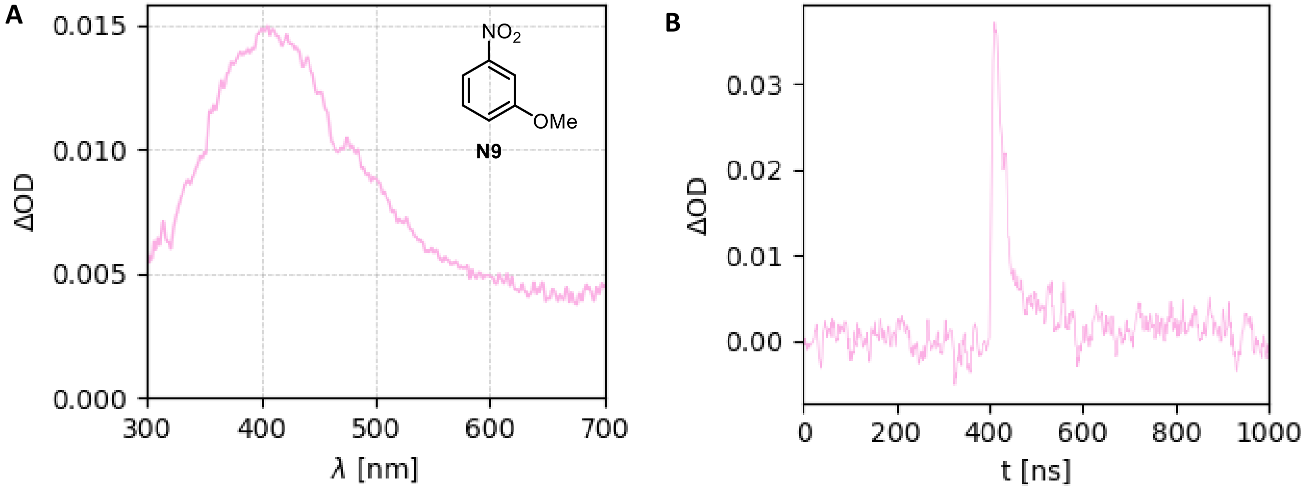

Supplementary Figure 15. **A**. Transient absorption spectrum of **N9** in deaerated CH_2Cl_2 (λ_{exc} = 355 nm). **B**. Transient decay trace for **N9** in deaerated CH_2Cl_2 (λ_{exc} = 355 nm) monitored at 450 nm. The decay was fitted to a monoexponential function, yielding

τ = 29 ns.

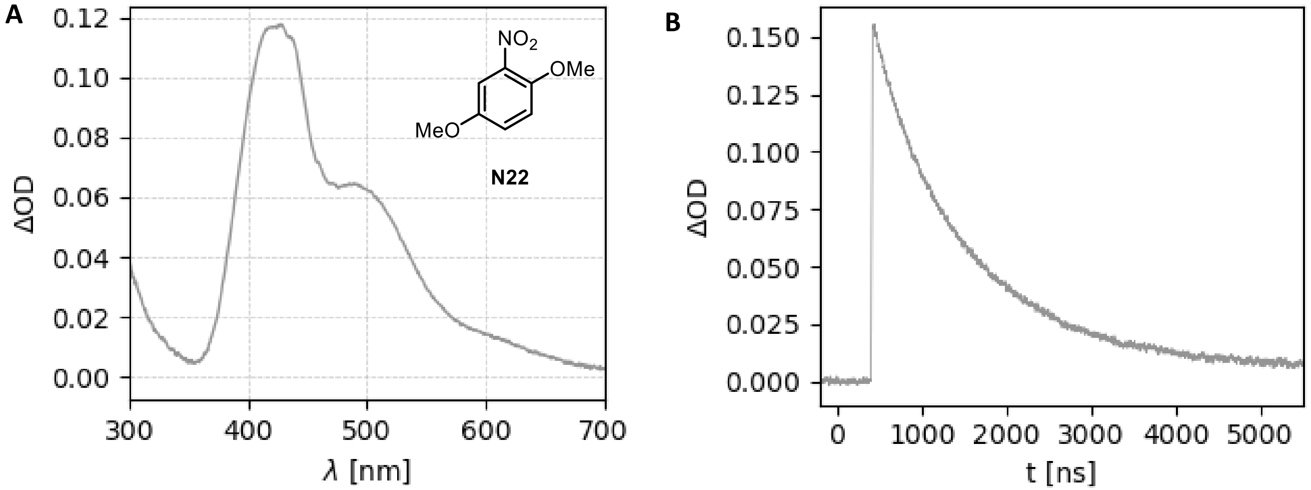

Supplementary Figure 16. **A**. Transient absorption spectrum of **N22** in deaerated CH_2Cl_2 (λ_{exc} = 266 nm). **B**. Transient decay trace for **N22** in deaerated CH_2Cl_2 (λ_{exc} = 266 nm) monitored at 425 nm. The decay was fitted to a monoexponential function, yielding τ = 1.0 μs.

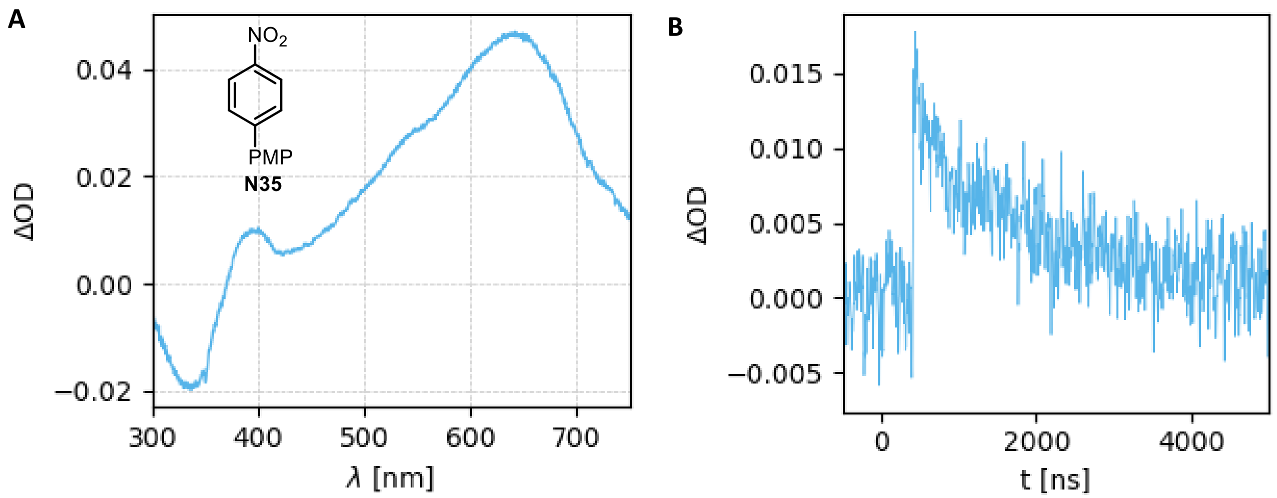

Supplementary Figure 17. **A.** Transient absorption spectrum of **N35** in deaerated CH_2Cl_2 (λ_{exc} = 355 nm). **B**. Transient decay trace for **N35** in deaerated CH_2Cl_2 (λ_{exc} = 355 nm) monitored at 640 nm. The decay was fitted to a monoexponential function, yielding τ = 1.2 μs.

Computational Absorption Spectra of ^{3}N23

As **N23** is the only nitroarene in our dataset with a low-lying ^{3}n,π* state that produces a transient signal, we wanted to corroborate that we are really detecting said triplet state or if it is the very low population of the ^{3}π,π* state the responsible for the transient spectrum. To this end, we decided to simulate the excited-state absorption of these triplet states using TD-DFT (Supplementary Figure 18). According to these results, the ^{3}π,π* state would show a transient signal at *ca*. 500 nm, which is not observed in the experiments (see Supplementary Figure 14). In contrast, the experimental spectrum correlates well with the calculated one for the ^{3}n,π* state, with bands at *ca*. 300 and 600 nm.

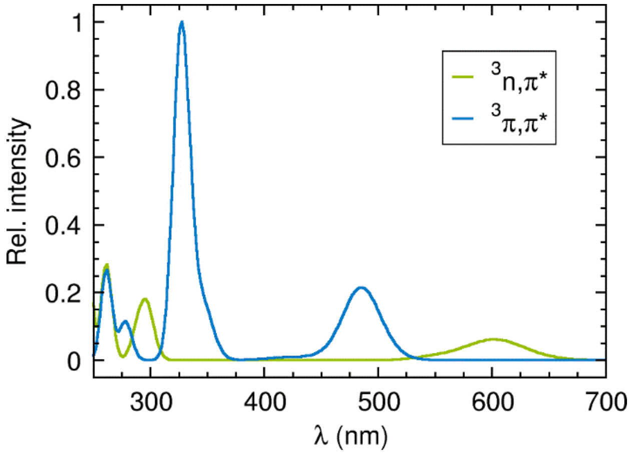

Supplementary Figure 18. Calculated UV-vis absorption spectra for the ^{3}n,π* (green) and ^{3}π,π* (blue) states of **N23**.

Supplementary Note 10: Intramolecular [2+2] Cycloaddition

Optimization

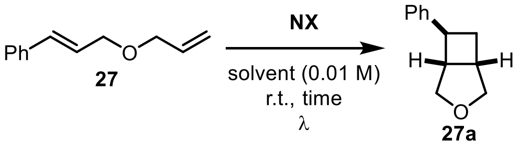

An oven-dried microwave tube containing a stir-bar was charged with **27** (9 mg, 0.05 mmol, 1.0 equiv.) and the nitroarene (10 mol%). The vial was capped with a Supelco aluminium crimp seal with septum (PTFE/butyl) and was evacuated and refilled with Ar (x 3). Dry and degassed solvent (5 mL, 0.01 M) was added, and the reaction mixture was stirred (> 500 rpm) under irradiation for the specified time. A solution of 1,3-dinitrobenzene (250 µL of 0.2 M CH_3CN solution) was added to the reaction mixture and the sample was concentrated. The residue was dissolved in $CDCl_3$ (700 µL) and analyzed using quantitative ^{1}H NMR spectroscopy to obtain ^{1}H NMR yield and diastereoselectivity.

Supplementary Table 4. Optimization table for the [2+2]-cycloaddition of **27**.

Entry	Nitroarene	Nitroarene loading (mol%)	Wavelength (nm)	Solvent	Time	27a (%)	d.r.
**1**	**N1**	10	390	CH_2Cl_2	24 h	18	n.d.
**2**	**N9**	10	390	CH_2Cl_2	24 h	63	6:1
**3**	**N14**	10	390	CH_2Cl_2	24 h	15	n.d.
**4**	**N15**	10	390	CH_2Cl_2	24 h	28	n.d.
**5**	**N16**	10	390	CH_2Cl_2	24 h	13	n.d.
**6**	**N18**	10	390	CH_2Cl_2	24 h	18	n.d.
**7**	**N24**	10	390	CH_2Cl_2	24 h	58	6:1
**8**	**N25**	10	390	CH_2Cl_2	24 h	41	6:1
**9**	**N29**	10	390	CH_2Cl_2	24 h	51	7:1
**10**	**N33**	10	390	CH_2Cl_2	24 h	3	n.d.
**11**	**N34**	10	390	CH_2Cl_2	24 h	72	8:1
**12**	**N35**	10	390	CH_2Cl_2	24 h	24	n.d.
**13**	**N36**	10	390	CH_2Cl_2	24 h	74	5:1
**14**	**N34**	10	390	CH_3CN	24 h	19	n.d.
**15**	**N34**	10	390	THF	24 h	53	6:1
**16**	**N34**	10	390	$PhCF_3$	24 h	19	n.d.
**17**	**N34**	10	390	DMF	24 h	46	7:1
**18**	**N34**	10	390	EtOAc	24 h	27	n.d.
**19**	**N34**	10	390	EtOH	24 h	43	6:1

**20**	**N34**	10	390	HFIP	24 h	40	6:1
**21**	**N34**	10	390	DMSO	24 h	38	n.d.
**22**	**N34**	10	390	CH_2Cl_2 (0.02 M)	24 h	56	7:1
**23**	**N34**	10	390	CH_2Cl_2 (0.05 M)	24 h	61	6:1
**24**	**N34**	10	390	CH_2Cl_2 (0.10 M)	24 h	55	7:1
**25**	**N34**	2.5	390	CH_2Cl_2	24 h	65	7:1
**26**	**N34**	5	390	CH_2Cl_2	24 h	71	7:1
**27**	**N34**	20	390	CH_2Cl_2	24 h	56	9:1
**28**	**N34**	10	427	CH_2Cl_2	24 h	6	n.d.
**29**	**N34**	10	390	CH_2Cl_2	1 h	1	n.d.
**30**	**N34**	10	390	CH_2Cl_2	2 h	3	n.d.
**31**	**N34**	10	390	CH_2Cl_2	4 h	8	n.d.
**32**	**N34**	10	390	CH_2Cl_2	6 h	18	n.d.
**33**	**N34**	10	390	CH_2Cl_2	8 h	24	n.d.
**34**	**N34**	10	390	CH_2Cl_2	16 h	45	n.d.
**35**	**N34**	-	390	CH_2Cl_2	24 h	0	n.d.
**36**	**N34**	10	-	CH_2Cl_2	24 h	0	n.d.
**37**	**N34**	10	390	CH_2Cl_2 (air)	24 h	19	n.d.

Intramolecular [2+2] Cycloaddition Time Profile

The time profile for the cycloaddition reaction using compound **27** was undertaken to understand the course of the reaction (Supplementary Figure 19). From the reaction monitoring it was evident that the initial reaction was not the cycloaddition but the *E*/*Z* isomerization. At around the 6 h mark the maximum concentration of the *Z*-product is reached then decreases. Meanwhile, the cycloaddition product **27a** shows a steady increase in concentration throughout the experiment.[19]

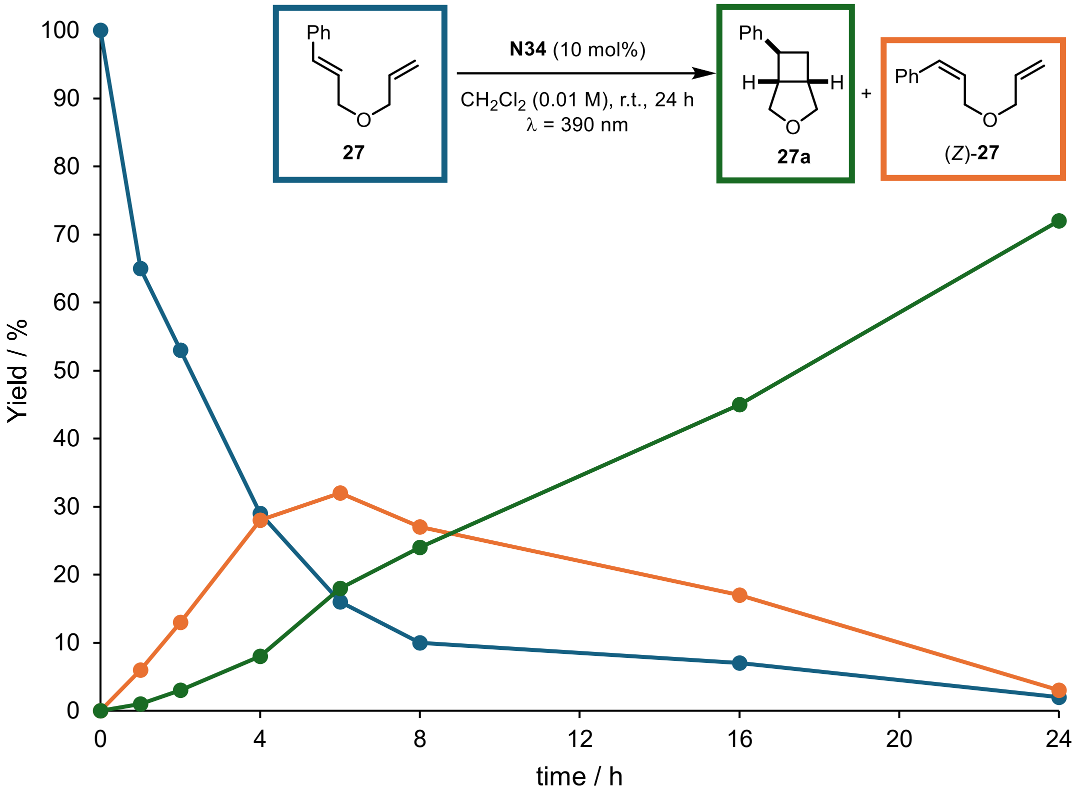

Supplementary Figure 19. Time profile for [2+2] cycloaddition of **27**.

Transient Absorption Spectra and Stern−Volmer Experiments for [2+2] Cycloaddition

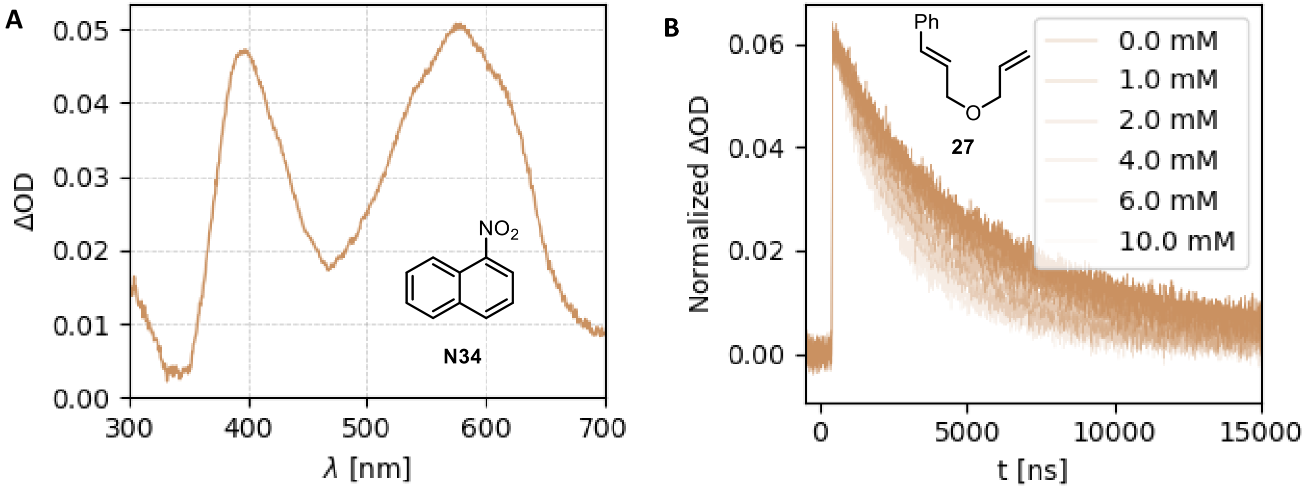

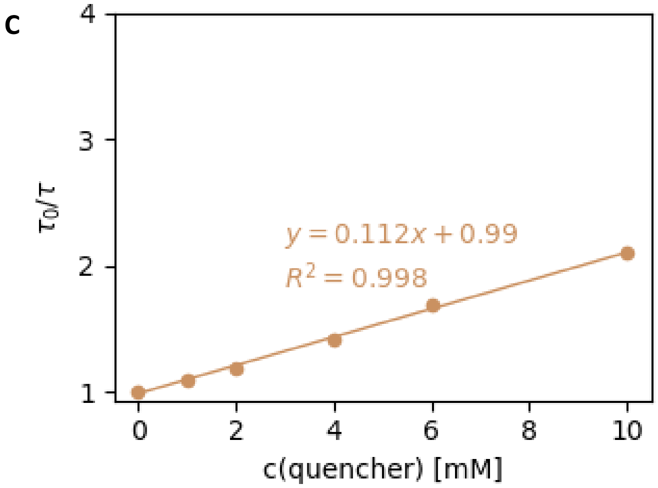

D

Q [mM]	τ [ns]	τ_0/τ
0.0	4975	1.000
1.0	4541	1.096
2.0	4173	1.192
4.0	3506	1.419
6.0	2957	1.682
10.0	2372	2.097
K_q	$0.23*10^8$ M^{-1} s^{-1}	

Supplementary Figure 20. **A**. Transient absorption spectrum of **N34** in deaerated CH_2Cl_2 (λ_{exc} = 355 nm). **B**. Normalized transient decay traces for **N34** in deaerated CH_2Cl_2 (λ_{exc} = 355 nm) monitored at 566 nm upon addition of increasing amounts of **27**. In all cases, the decays were fitted to a monoexponential function. **C**. Corresponding Stern−Volmer plot. **D**. Apparent lifetimes τ at increasing concentrations of quencher and calculated K_q.

Supplementary Note 11: Optimization of other EnT transformations

Intermolecular [2+2] cycloaddition

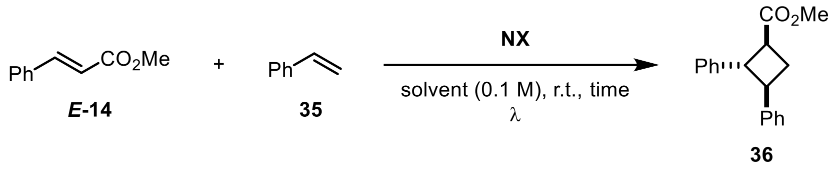

An oven-dried microwave tube containing a stir-bar was charged with ***E*-14** (16 mg, 0.1 mmol, 1.0 equiv.) and the nitroarene (10 mol%). The vial was capped with a Supelco aluminium crimp seal with septum (PTFE/butyl) and was evacuated and refilled with Ar (x 3). Styrene (115μL, 1.0 mmol, 10.0 equiv.) and dry and degassed solvent (1 mL, 0.1 M) were added, and the reaction mixture was stirred (> 500 rpm) under irradiation for the specified time. A solution of 1,3,5-trimethoxybenzene (0.5 mL of 0.2 M $CDCl_3$ solution) was added to the reaction mixture. An aliquot (100 μL) of the mixture was transferred to a NMR tube, diluted with 600 μL of $CDCl_3$ and analyzed using quantitative ^{1}H NMR spectroscopy to obtain ^{1}H NMR yield and the diastereoselectivity.

Supplementary Table 5. Optimization table for the intermolecular [2+2]-cycloaddition of ***E*-14**.

Entry	Nitroarene	Loading (mol%)	Wavelength (nm)	Solvent	Time	36 (%)	d.r.
**1**	**N9**	10	390	1,2-DCE	16 h	7	1.3:1
**2**	**N15**	10	390	1,2-DCE	16 h	8	1.7:1
**3**	**N17**	10	390	1,2-DCE	16 h	3	2.0:1
**4**	**N18**	10	390	1,2-DCE	16 h	1	1.0:0
**5**	**N24**	10	390	1,2-DCE	16 h	39	2.0:1
**6**	**N25**	10	390	1,2-DCE	16 h	7	1.3:1
**7**	**N33**	10	390	1,2-DCE	16 h	8	1.7:1
**8**	**N34**	10	390	1,2-DCE	16 h	13	2.2:1
**9**	**N35**	10	390	1,2-DCE	16 h	37	2.1:1
**10**	**N36**	10	390	1,2-DCE	16 h	6	2.0:1
**11**	**N24**	15	390	1,2-DCE	16 h	48	2.2:1
**12**	**N24**	20	390	1,2-DCE	16 h	56	2.1:1
**13**	**N24**	25	390	1,2-DCE	16 h	57	2.0:1
**14**	**N24**	20	390	CH_3CN	16 h	66	1.9:1
**15**	**N24**	20	390	$PhCH_3$	16 h	71	2.6:1
**16**	**N24**	20	390	THF	16 h	76	2.2:1
**17**	**N24**	20	390	EtOAc	16 h	71	2.0:1

**18**	**N24**	20	390	EtOH	16 h	33	1.7:1
**19**	**N24**	20	390	DMF	16 h	61	1.4:1
**20**	**N24**	20	390	DMSO	16 h	58	1.8:1
**21**	**N24**	20	390	Dioxane	16 h	58	2.2:1
**22**	**N24**	20	390	CH_2Cl_2	16 h	51	2.4:1
**23**	**N24**	20	390	THF (0.2 M)	16 h	75	2.3:1
**24**	**N24**	20	390	THF (0.05 M)	16 h	56	1.9:1
**25**	**N24**	20	390	THF (0.033 M)	16 h	34	2.1:1
**26**	**N24**	20 (Styrene 5.0 eq.)	390	THF	16 h	58	2.2:1
**27**	**N24**	20 (Styrene 5.0 eq.)	390	THF	40 h	60	2.2:1
**28**	**N24**	10	390	THF	16 h	45	2.0:1
**29**	**N24**	10	390	THF	40 h	44	2.4:1
**30**	-	-	390	THF	16 h	0	n.d.
**31**	**N24**	20	-	THF	16 h	0	n.d.

Aza-Paternò–Büchi

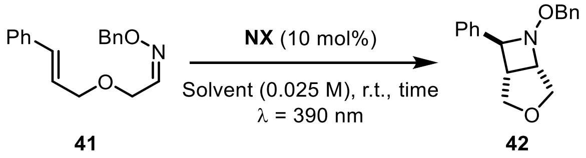

An oven-dried microwave tube containing a stir-bar was charged with **41** (14 mg, 0.05 mmol, 1.0 equiv.) and the nitroarene (10 mol%). The vial was capped with a Supelco aluminium crimp seal with septum (PTFE/butyl) and was evacuated and refilled with Ar (x 3). Dry and degassed solvent (2 mL, 0.025 M) was added, and the reaction mixture was stirred (> 500 rpm) under 390 nm irradiation for the specified time. A solution of 1,3-dinitrobenzene (250 µL of 0.4 M $CDCl_3$ solution) was added to the reaction mixture. An aliquot (100 µL) of the mixture was transferred to a NMR tube, diluted with 600 µL of $CDCl_3$ and analyzed using quantitative ^{1}H NMR spectroscopy to obtain ^{1}H NMR yield.

Supplementary Table 6. Optimization table for the intramolecular cycloaddition of **41**.

Entry	Nitroarene	Concentration (M)	Solvent	Time	42 (%)
**1**	**N24**	0.025	Me_3CN	1 h	17
**2**	**N24**	0.025	CH_2Cl_2	1 h	19
**3**	**N24**	0.025	THF	1 h	15
**4**	**N24**	0.025	EtOAc	1 h	54
**5**	**N34**	0.025	Me_3CN	1 h	0
**6**	**N34**	0.025	CH_2Cl_2	1 h	0
**7**	**N34**	0.025	THF	1 h	0
**8**	**N34**	0.025	EtOAc	1 h	0
**9**	**N24**	0.1	EtOAc	1 h	56
**10**	**N24**	0.05	EtOAc	1 h	58
**11**	**N24**	0.01	EtOAc	1 h	59
**12**	**N24**	0.025	EtOAc	30 min	58
**13**	**-**	0.025	EtOAc	1 h	0

Translocation

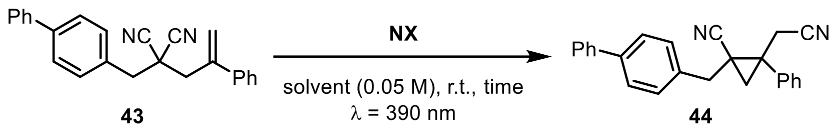

An oven-dried microwave tube containing a stir-bar was charged with **43** (17 mg, 0.05 mmol, 1.0 equiv.) and the nitroarene (10 mol%). The vial was capped with a Supelco aluminium crimp seal with septum (PTFE/butyl) and was evacuated and refilled with Ar (x 3). Dry and degassed solvent (1 mL, 0.05 M) was added, and the reaction mixture was stirred (> 500 rpm) under irradiation for the specified time. A solution of 1,3,5-trimethoxybenzene (1.0 mL of 0.0333 M $CDCl_3$ solution) was added to the reaction mixture. An aliquot (100 µL) of the mixture was transferred to a NMR tube, diluted with 600 µL of $CDCl_3$ and analyzed using quantitative ^{1}H NMR spectroscopy to obtain ^{1}H NMR yield and the diastereoselectivity.

Supplementary Table 7. Optimization table for the translocation reaction of **43**.

Entry	Nitroarene	Loading (mol%)	Solvent	Time	44 (%)	d.r.
**1**	**N9**	10	EtOAc	20 h	8	n.d.
**2**	**N24**	10	EtOAc	20 h	16	n.d.
**3**	**N34**	10	EtOAc	20 h	0	n.d.
**4**	**N35**	10	EtOAc	20 h	14	n.d.
**5**	**N36**	10	EtOAc	20 h	24	1:1
**6**	**N36**	10	CH_3CN	20 h	9	n.d.
**7**	**N36**	10	CH_2Cl_2	20 h	21	1:1
**8**	**N36**	10	1,2-DCE	20 h	18	1:1
**9**	**N36**	10	$CHCl_3$	20 h	24	1:1
**10**	**N36**	10	dioxane	20 h	0	n.d.
**11**	**N36**	10	THF	20 h	0	n.d.
**12**	**N36**	10	EtOH	20 h	41	1:1
**13**	**N36**	10	$PhCF_3$	20 h	21	1:1
**14**	**N36**	10	acetone	20 h	0	n.d.
**15**	**N36**	10	DMSO	20 h	10	n.d.
**16**	**N36**	10	DMF	20 h	0	n.d.
**17**	**N36**	20	EtOH	20 h	31	n.d.
**18**	**N36**	10	EtOH	40 h	78	1:1
**19**	**-**	-	EtOH	20 h	0	n.d.

Supplementary Note 12: Substrate Scope

Ethyl (*Z*)-3-Phenylpent-2-enoate (*Z*-1)

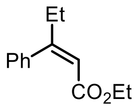

Following **GP6**, ***E*-1** (20 mg, 0.10 mmol, 1.0 equiv.) gave ***Z*-1** (quant., 99:1 *Z*/*E*) as an oil. ^1H NMR (600 MHz, $CDCl_3$) δ 7.37–7.32 (2H, m), 7.33–7.27 (1H, m), 7.18–7.13 (2H, m), 5.87 (1H, s), 3.99 (2H, q, J = 7.2 Hz), 2.46 (2H, q, J = 7.4 Hz), 1.07 (3H, t, J = 7.4 Hz), 1.06 (3H, t, J = 7.4 Hz); ^{13}C NMR (151 MHz, $CDCl_3$) δ 166.3, 161.2, 140.6, 128.0, 127.6, 127.1, 116.5, 59.9, 33.5, 14.1, 12.2. Data in accordance with the literature.[8]

Ethyl (*Z*)-3-Phenylbut-2-enoate (*Z*-3)

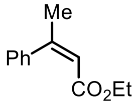

Following **GP6**, ***E*-3** (19 mg, 0.10 mmol, 1.0 equiv.) gave ***Z*-3** (quant., 96:4 *Z*/*E*) as an oil. ^1H NMR (600 MHz, $CDCl_3$) δ 7.37–7.33 (2H, m), 7.33–7.29 (1H, m), 7.21 (2H, d, J = 7.8 Hz), 5.91 (1H, q, J = 1.9 Hz), 4.00 (2H, q, J = 7.1 Hz), 2.18 (3H, d, J = 1.9 Hz), 1.08 (3H, t, J = 7.1 Hz); ^{13}C NMR (151 MHz, $CDCl_3$) δ 166.1, 155.5, 141.0, 128.0, 127.8, 126.9, 117.9, 59.9, 27.3, 14.1. Data in accordance with the literature.[8]

Ethyl (*E*)-4,4,4-Trifluoro-3-phenylbut-2-enoate (*E*-4)

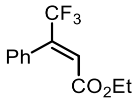

Following **GP6**, ***Z*-4** (24 mg, 0.10 mmol, 1.0 equiv.) gave ***E*-4** (quant., 8:92 *Z*/*E*) as an oil. ^1H NMR (600 MHz, $CDCl_3$) δ 7.45–7.38 (3H, m), 7.31–7.27 (2H, m), 6.61 (1H, q, J = 1.4 Hz), 4.05 (2H, q, J = 7.1 Hz), 1.06 (3H, t, J = 7.1 Hz); ^{13}C NMR (151 MHz, $CDCl_3$) δ 164.3, 142.5 (q, J = 31.1 Hz), 131.2, 129.4, 128.8, 128.3, 124.7 (q, J = 5.5 Hz), 122.7 (q, J = 274.9 Hz), 61.2, 13.8; ^{19}F NMR (564 MHz, $CDCl_3$) δ –67.58 (s). Data in accordance with the literature. [8]

Ethyl (*Z*)-3-(Pyridin-3-yl)but-2-enoate (*Z*-5)

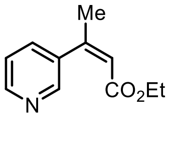

Following **GP6**, ***E*-5** (22 mg, 0.10 mmol, 1.0 equiv.) gave ***Z*-5** (quant., 99:1 *Z*/*E*) as an oil. ^1H NMR (600 MHz, $CDCl_3$) δ 8.53 (1H, dd, J = 4.9, 1.7 Hz), 8.44 (1H, dd, J = 2.3, 0.9 Hz), 7.53 (1H, dt, J = 7.8, 2.0 Hz), 7.27 (1H, ddd, J = 7.8, 4.8, 0.9 Hz), 5.99 (1H, q, J = 1.5 Hz), 4.00 (2H, q, J = 7.1 Hz), 2.18 (3H, d, J = 1.6 Hz), 1.08 (3H, t, J = 7.1 Hz); ^{13}C NMR (151 MHz, $CDCl_3$) δ 165.5, 151.8, 148.9, 147.9, 136.6, 134.5, 122.8, 119.5, 60.1, 27.0, 14.0. Data in accordance with the literature.[8]

Methyl (*Z*)-3-(4-Methoxyphenyl)but-2-enoate (*Z*-6)

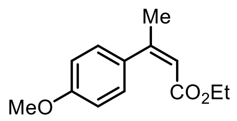

Following **GP6**, ***E*-6** (22 mg, 0.10 mmol, 1.0 equiv.) gave ***Z*-6** (quant., 96:4 *Z*/*E*) as an oil. ^{1}H NMR (600 MHz, $CDCl_3$) δ 7.22–7.16 (2H, m), 6.92–6.85 (2H, m), 5.87 (1H, q, *J* = 1.4 Hz), 4.04 (2H, q, *J* = 7.1 Hz), 3.81 (3H, s), 2.17 (3H, d, *J* = 1.5 Hz), 1.14 (3H, t, *J* = 7.1 Hz); ^{13}C NMR (151 MHz, $CDCl_3$) δ 166.2, 159.5, 155.0, 132.8, 128.7, 117.2, 113.3, 59.8, 55.3, 27.2, 14.2. Data in accordance with the literature.[9]

Ethyl (*Z*)-3-(3-Bromo-2-fluorophenyl)but-2-enoate (*Z*-7)

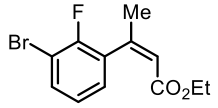

Following **GP6**, ***E*-7** (22 mg, 0.10 mmol, 1.0 equiv.) gave ***Z*-7** (quant., 95:5 *Z*/*E*) as an oil. R_f 0.56 [pentane:Et_2O (30:1)]; ^{1}H NMR (600 MHz, $CDCl_3$) δ 7.49 (1H, ddd, *J* = 8.1, 6.5, 1.8 Hz), 7.07 (1H, ddd, *J* = 7.9, 6.3, 1.8 Hz), 7.01 (1H, td, *J* = 7.8, 0.8 Hz), 6.03 (1H, q, *J* = 1.5 Hz), 4.01 (2H, q, *J* = 7.2 Hz), 2.16 (3H, d, *J* = 1.5 Hz), 1.09 (3H, t, *J* = 7.1 Hz); ^{13}C NMR (151 MHz, $CDCl_3$) δ 165.1, 154.9 (d, *J* = 246.0 Hz), 148.3, 132.8, 130.3 (d, *J* = 17.8 Hz), 127.9 (d, *J* = 3.3 Hz), 124.8 (d, *J* = 4.5 Hz), 120.9, 109.3 (d, *J* = 21.6 Hz), 60.1, 26.3, 14.0; ^{19}F NMR (564 MHz, $CDCl_3$) δ –109.70 (t, *J* = 6.4 Hz); HRMS (ESI) found $[M+Na]^+$ 308.98922, $C_{12}H_{12}O_2BrFNa$ requires 308.98969.

Ethyl (*Z*)-3-(4-Bromophenyl)pent-2-enoate (*Z*-8)

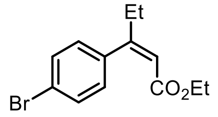

Following **GP6**, ***E*-8** (28 mg, 0.10 mmol, 1.0 equiv.) gave ***Z*-8** (quant., 99:1 *Z*/*E*) as an oil. ^{1}H NMR (600 MHz, $CDCl_3$) δ 7.49–7.45 (2H, m), 7.05–7.01 (2H, m), 5.88 (1H, t, *J* = 1.4 Hz), 4.00 (2H, q, *J* = 7.2 Hz), 2.43 (2H, qd, *J* = 7.4, 1.4 Hz), 1.11 (3H, t, *J* = 7.1 Hz), 1.04 (3H, t, *J* = 7.4 Hz); ^{13}C NMR (151 MHz, $CDCl_3$) δ 166.0, 160.0, 139.4, 131.2, 128.9, 121.7, 116.9, 60.0, 33.4, 14.1, 12.2. Data in accordance with the literature.[10]

(*Z*)-4,4,5,5-Tetramethyl-2-(2-phenylbut-1-en-1-yl)-1,3,2-dioxaborolane (*Z*-9)

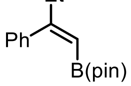

Following **GP7**, ***E*-9** (26 mg, 0.10 mmol, 1.0 equiv.) gave ***Z*-9** (98%, 99:1 *Z*/*E*) as an oil. ^{1}H NMR (600 MHz, $CDCl_3$) δ 7.31–7.20 (5H, m), 5.45 (1H, s), 2.48 (2H, q, *J* = 7.3 Hz), 1.12 (12H, s), 1.04 (3H, t, *J* = 7.4 Hz); ^{13}C NMR (151 MHz, $CDCl_3$) δ 163.8, 143.3, 128.0, 127.7, 127.3, 83.1, 33.5, 24.7, 12.7 (the boron-bearing carbon was not observed due to fast quadrupole relaxation); ^{11}B NMR (193 MHz, $CDCl_3$) δ 30.27. Data in accordance with the literature.[11]

(*Z*)-4,4,5,5-Tetramethyl-2-(2-phenylprop-1-en-1-yl)-1,3,2-dioxaborolane (*Z*-10)

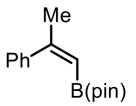

Following **GP7**, ***E*-10** (24 mg, 0.10 mmol, 1.0 equiv.) gave ***Z*-10** (97%, 96:4 *Z*/*E*) as an oil. ^{1}H NMR (600 MHz, $CDCl_3$) δ 7.39–7.26 (5H, m), 5.50 (1H, s), 2.24 (3H, d, *J* = 1.3 Hz), 1.17 (12H, s); ^{13}C NMR (151 MHz, $CDCl_3$) δ 157.8, 143.3, 127.7, 127.7, 127.5, 83.1, 27.9, 24.7 (the boron-bearing carbon was not observed due to fast quadrupole relaxation); ^{11}B NMR (193 MHz, $CDCl_3$) δ 29.96. Data in accordance with the literature.[11]

(*Z*)-2-(2-(4-Bromophenyl)prop-1-en-1-yl)-4,4,5,5-tetramethyl-1,3,2-dioxaborolane (*Z*-11)

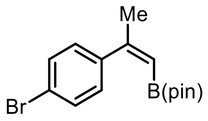

Following **GP7**, ***E*-11** (32 mg, 0.10 mmol, 1.0 equiv.) gave ***Z*-11** (quant., 93:7 *Z*/*E*) as an oil. ^{1}H NMR (600 MHz, $CDCl_3$) δ 7.40 (2H, d, *J* = 8.3 Hz), 7.18 (2H, d, *J* = 8.3 Hz), 5.48 (1H, s), 2.18 (3H, s), 1.15 (12H, s); ^{13}C NMR (151 MHz, $CDCl_3$) δ 156.6, 142.1, 130.7, 129.5, 121.5, 83.2, 27.8, 24.7 (the boron-bearing carbon was not observed due to fast quadrupole relaxation); ^{11}B NMR (193 MHz, $CDCl_3$) δ 30.01. Data in accordance with the literature.[11]

(*Z*)-4,4,5,5-Tetramethyl-2-(2-(4-(trifluoromethyl)phenyl)prop-1-en-1-yl)-1,3,2-dioxaborolane (*Z*-12)

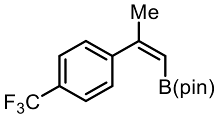

Following **GP7**, ***E*-12** (31 mg, 0.10 mmol, 1.0 equiv.) gave ***Z*-12** (95%, 96:4 *Z*/*E*) as an oil. R_f 0.58 [pentane:Et_2O (30:1)]; ^{1}H NMR (600 MHz, $CDCl_3$) δ 7.54 (2H, d, *J* = 7.9 Hz), 7.39 (2H, d, *J* = 7.8 Hz), 5.56 (1H, s), 2.21 (3H, s), 1.13 (12H, s); ^{13}C NMR (151 MHz, $CDCl_3$) δ 156.6, 147.0, 129.5 (q, *J* = 32.3 Hz), 128.1, 124.6 (q, *J* = 3.8 Hz), 124.5 (q, *J* = 271.9 Hz), 83.3, 27.8, 24.7 (the boron-bearing carbon was not observed due to fast quadrupole relaxation); ^{11}B NMR (193 MHz, $CDCl_3$) δ 29.68; ^{19}F NMR (564 MHz, $CDCl_3$) δ –62.45 (s); HRMS (ESI) found $[M+Na]^+$ 335.13921, $C_{16}H_{20}O_2BF_3Na$ requires 335.14007.

(*Z*)-4,4,5,5-Tetramethyl-2-(2-(m-tolyl)prop-1-en-1-yl)-1,3,2-dioxaborolane (*Z*-13)

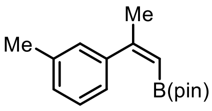

Following **GP7**, ***E*-13** (32 mg, 0.10 mmol, 1.0 equiv.) gave ***Z*-13** (quant., 96:4 *Z*/*E*) as an oil. ^{1}H NMR (600 MHz, $CDCl_3$) δ 7.18 (1H, t, *J* = 7.5 Hz), 7.15–7.10 (2H, m), 7.08 (1H, d, *J* = 7.6 Hz), 5.45 (1H, s), 2.34 (3H, s), 2.21 (3H, s), 1.16 (12H, s); ^{13}C NMR (151 MHz, $CDCl_3$) δ 157.8, 143.2, 137.0, 128.6, 128.3, 127.7, 124.7, 83.0, 27.8, 24.8, 21.5 (the boron-bearing

carbon was not observed due to fast quadrupole relaxation); ^{11}B NMR (193 MHz, $CDCl_3$) δ 30.13. Data in accordance with the literature.[11]

Methyl (*Z*)-3-Phenylacrylate (*Z*-14)

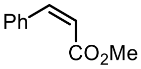

Following **GP6**, (*E*)-methyl cinnamate (*E*)- **14** (16 mg, 0.10 mmol, 1.0 equiv.) gave ***Z*-14** (quant., 71:29 *Z*/*E*) as an oil. ^{1}H NMR (600 MHz, $CDCl_3$) δ 7.59 (2H, d, *J* = 7.3 Hz), 7.42–7.29 (3H, m), 6.96 (1H, d, *J* = 12.6 Hz), 5.96 (1H, d, *J* = 12.6 Hz), 3.72 (3H, s); ^{13}C NMR (151 MHz, $CDCl_3$) δ 166.7, 143.5, 134.9, 129.8, 129.2, 128.2, 119.4, 51.5. Data in accordance with the literature.[8]

(*Z*)-3-Phenylprop-2-en-1-ol (*Z*-15)

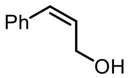

Following **GP6**but with **N34** (2 mg, 10 mol%), CH_3CN (2 mL, 0.05 M), and 60 h irradiation, (*E*)-cinnamyl alcohol (13 mg, 0.1 mmol, 1.0 equiv.) gave ***Z*-15** (59%, 78:22 *Z*/*E*) as an oil. ^{1}H NMR (400 MHz, $CDCl_3$) δ 7.35 (2H, t, *J* = 7.5 Hz), 7.33–7.24 (1H, m), 7.21 (2H, d, *J* = 7.5 Hz), 6.58 (1H, d, *J* = 11.7 Hz), 5.88 (1H, dt, *J* = 12.2, 6.4 Hz), 4.45 (2H, d, *J* = 6.4 Hz), 1.53 (1H, s); ^{13}C NMR (101 MHz, $CDCl_3$) δ 136.7, 131.3, 131.3, 129.0, 128.5, 127.4, 59.9. Data in accordance with the literature.[28]

Ethyl (*Z*)-3-(Pyridin-2-yl)acrylate (*Z*-16)

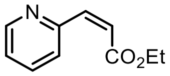

Following **GP6** but with **N24** (2 mg, 10 mol%), CH_3CN (2 mL, 0.05 M), and 6 h irradiation, ***E*-16** (18 mg, 0.1 mmol, 1.0 equiv.) gave ***Z*-16** (90%, 94:6 *Z*/*E*) as an oil. ^{1}H NMR (600 MHz, $CDCl_3$) δ 8.58 (1H, ddd, *J* = 4.9, 1.9, 1.1 Hz), 7.68–7.59 (2H, m), 7.19 (1H, ddd, *J* = 7.1, 4.8, 1.5 Hz), 6.93 (1H, d, *J* = 12.5 Hz), 6.12 (1H, d, *J* = 12.5 Hz), 4.20 (2H, q, *J* = 7.2 Hz), 1.24 (3H, t, *J* = 7.1 Hz); ^{13}C NMR (151 MHz, $CDCl_3$) δ 166.9, 153.7, 149.3, 139.8, 136.1, 124.5, 123.2, 123.2, 60.7, 14.2. Data in accordance with the literature.[29]

(*Z*)-3-Phenylallyl acetate (*Z*-17)

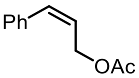

Following **GP6** but with **N24** (2 mg, 10 mol%), CH_3CN (2 mL, 0.05 M), and 6 h irradiation, ***E*-17** (18 mg, 0.1 mmol, 1.0 equiv.) gave ***Z*-17** (84%, 82:18 *Z*/*E*) as an oil. ^{1}H NMR (400 MHz, $CDCl_3$) δ 7.35–7.28 (2H, m), 7.28–7.21 (1H, m), 7.21–7.15 (2H, m), 6.64 (1H, dt, *J* = 11.9, 1.4 Hz Hz), 5.78 (1H, dt, *J* = 11.8, 6.6 Hz), 4.81 (2H, dd, *J* = 6.6, 1.7 Hz), 2.05 (3H,

s); ^{13}C NMR (151 MHz, $CDCl_3$) δ 171.1, 136.2, 133.2, 128.9, 128.6, 127.7, 126.0, 61.7, 21.2. Data in accordance with the literature.[9]

(*Z*)-3-Phenylpent-2-enal (*Z*-18)

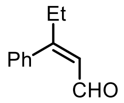

Following **GP6**, but with **N24** (2 mg, 10 mol%), ***E*-18** (16 mg, 0.1 mmol, 1.0 equiv.) gave ***Z*-18** (86%, Z/E 99:1) as an oil. ^{1}H NMR ($CDCl_3$, 400 MHz) δ 9.38 (1H, d, J = 8.1 Hz), 7.40–7.29 (3H, m), 7.23–7.16 (2H, m), 6.04 (1H, ddt, J = 8.1, 1.3 Hz), 2.53 (2H, qd, J = 7.5, 1.3 Hz), 1.03 (3H, t, J = 7.4 Hz); ^{13}C NMR ($CDCl_3$, 101 MHz) δ 194.0, 168.1, 138.1, 129.0, 128.6, 128.5, 127.6, 32.9, 12.1. Data in accordance with the literature.[8]

(*Z*)-3-Phenylbut-2-enenitrile (*Z*-19)

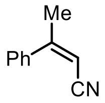

Following **GP6**, but with **N24** (4 mg, 10 mol%) and 10 min irradiation, ***E*-19** (29 mg, 0.2 mmol, 1 equiv.) gave ***Z*-19** (96%, Z/E 86:14) as an oil. ^{1}H NMR ($CDCl_3$, 400 MHz) δ 7.58–7.50 (2H, m), 7.48–7.38 (3H, m), 5.40 (1H, q, J = 1.5 Hz), 2.29 (3H, d, J = 1.4 Hz); ^{13}C NMR ($CDCl_3$, 101 MHz) δ 161.1, 138.0, 130.0, 128.8, 127.2, 117.7, 95.6, 24.8. Data in accordance with the literature.[16]

(*Z*)-3-Phenylpent-2-enoic Acid (*Z*-20)

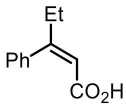

Following **GP6**, but with **N24** (2 mg, 10 mol%), CH_3CN (0.05 M) and 30 min irradiation, gave ***E*-20** (18 mg, 0.1 mmol, 1.0 equiv.) gave ***Z*-20** (95%, Z/E 99:1) as a solid. ^{1}H NMR (DMSO-d_6, 400 MHz) δ 11.84 (1H, s), 7.34–7.23 (3H, m), 7.15 (2H, d, J = 7.2 Hz), 5.81 (1H, s), 2.40 (2H, q, J = 7.3 Hz), 0.92 (3H, t, J = 7.4 Hz); ^{13}C NMR (DMSO-d_6, 101 MHz) δ 166.9, 158.6, 140.1, 127.7, 127.3, 127.2, 117.0, 32.4, 12.1. Data in accordance with the literature.[30]

(*Z*)-3-(4-Bromophenyl)pent-2-enoic Acid (*Z*-22)

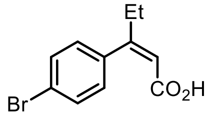

Following **GP6**, but with **N24** (2 mg, 10 mol%), CH_3CN (0.05 M) and 30 min irradiation**,** ***E*-22** (26 mg, 0.1 mmol, 1.0 equiv.) gave ***E*-22** (96%, Z/E 99:1) as a solid. R_f 0.64 [pentane:Et_2O(1:1)]; ^{1}H NMR (MeOD-d_4, 400 MHz) δ 7.49 (2H, d, J = 8.5 Hz), 7.10 (2H, d, J = 8.4 Hz), 5.91 (1H, t, J = 1.4 Hz), 2.48 (2H, qd, J = 7.5, 1.4 Hz), 1.03 (3H, t, J = 7.4 Hz); ^{13}C

NMR (MeOD-d_4, 101 MHz) δ 169.4, 161.3, 140.8, 132.0, 130.3, 122.4, 118.0, 34.1, 12.5. HRMS (APCI) Found $[M + H]^+$ 255.0011, $C_8H_9O_4NNa$ requires 255.0021.

(*Z*)-3-(3-Bromo-2-fluorophenyl)but-2-enoic Acid (*Z*-23)

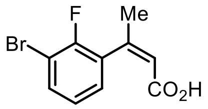

Following **GP6**, but with **N24** (2 mg, 10 mol%), CH_3CN (0.05 M) and 30 min irradiation, ***E*-23** (26 mg, 0.1 mmol, 1.0 equiv.) gave ***Z*-23** (quant., Z/E 90:10) as a solid. R_f 0.79 [pentane:Et_2O(1:1)]; ^1H NMR (MeOD-d_4, 600 MHz) δ 7.52 (1H, ddd, J = 8.1, 6.6, 1.6 Hz), 7.15 (1H, ddd, J = 7.9, 6.6, 1.6 Hz), 7.06 (1H, t, J = 7.8 Hz), 6.06 (1H, q, J = 1.5 Hz), 2.15 (3H, d, J = 1.4 Hz); ^{13}C NMR (MeOD-d_4, 151 MHz) δ 168.4, 156.0 (d, J = 245.2 Hz), 149.8, 134.8, 133.7, 131.7 (d, J = 17.7 Hz), 129.3 (d, J = 2.8 Hz), 126.2 (d, J = 4.2 Hz), 121.9, 109.7 (d, J = 22.0 Hz), 26.3; ^{19}F NMR (MeOD-d_4, 564 MHz) δ -111.8 (t, J = 6.5 Hz). HRMS (APCI) Found $[M + H]^+$ 258.9761, $C_8H_9O_4NNa$ requires 258.9770.

Methyl (Z)-2-Methyl-3-phenylacrylate (*Z*-24)

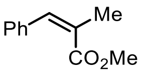

Following **GP6**, but using **N35** (7 mg, 10 mol%) and 1 h reaction time, ***E*-24** (38 mg, 0.2 mmol, 1 equiv.) gave ***Z*-24** (quant. Z/E 79:21) as an oil. ^1H NMR ($CDCl_3$, 400 MHz) δ 7.25–7.12 (5H, m), 6.63 (1H, s), 4.04 (2H, q, J = 7.1 Hz), 2.02 (3H, d, J = 1.4 Hz), 1.03 (3H, t, J = 7.1 Hz); ^{13}C NMR ($CDCl_3$, 101 MHz) δ 169.7, 136.5, 134.3, 130.2, 128.1, 128.0, 127.5, 60.6, 21.4, 13.8. Data in accordance with the literature.[9]

(Z)-1,2-Diphenylethene (*Z*-25)

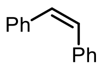

Following **GP6**, but using **N24** (7 mg, 20 mol%), CH_3CN (2 mL) and 10 minute irradiation, ***E*-25** (36 mg, 0.2 mmol, 1.0 equiv.) gave ***Z*-25** (99%, Z/E 70:30) as a solid. ^1H NMR ($CDCl_3$, 600 MHz) δ 7.29–7.17 (10H, m), 6.61 (2H, s); ^{13}C NMR ($CDCl_3$, 151 MHz) δ 137.4, 130.4, 129.0, 128.4, 127.3. Data in accordance with the literature.[31]

(*Z*)-1,2-Di(pyridin-4-yl)ethene (*Z*-26)

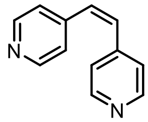

Following **GP6**, but using **N24** (7 mg, 20 mol%), CH_3CN (2 mL) and 16 h irradiation, ***E*-26** (36 mg, 0.2 mmol, 1.0 equiv.) and gave, ***Z*-26** (93%, Z/E 83:17) as a solid. ^1H NMR ($CDCl_3$, 600 MHz) δ 8.61–8.38 (4H, m), 7.12–7.02 (4H, m), 6.71 (2H, s); ^{13}C NMR ($CDCl_3$, 151 MHz) δ 150.3, 144.0, 131.3, 123.5. Data in accordance with the literature.[32]

(1*S,5*R**,6*S**)-6-Phenyl-3-oxabicyclo[3.2.0]heptane (27a)**

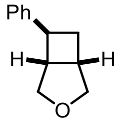

Following **GP8**, **27** (9 mg, 0.05 mmol, 1.0 equiv.) and **N34** (1 mg, 10 mol%) gave **27a** (72%, 8:1 d.r.) as an oil. ^{1}H NMR (400 MHz, $CDCl_3$) δ 7.37–7.29 (2H, m), 7.29–7.23 (2H, m), 7.23–7.15 (1H, m), 4.00 (1H, d, *J* = 6.7 Hz), 3.98 (1H, d, *J* = 6.9 Hz), 3.62 (1H, dd, *J* = 9.3, 5.6 Hz), 3.52 (1H, dd, *J* = 9.3, 4.6 Hz), 3.24 (1H, td, *J* = 8.4, 5.2 Hz), 3.09–2.88 (2H, m), 2.31 (1H, dt, *J* = 12.3, 8.1 Hz), 2.17 (1H, ddd, *J* = 12.6, 9.5, 3.8 Hz); ^{13}C NMR (151 MHz, $CDCl_3$) δ 146.3, 128.6, 126.6, 126.1, 74.7, 74.2, 47.4, 42.1, 35.5, 32.0. Data in accordance with the literature.[33]

(1*R,5*R**,7*S**)-6,6-Dimethyl-7-phenyl-3-oxabicyclo[3.2.0]heptane (28a)**

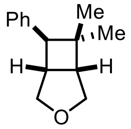

Following **GP8**, **28** (10 mg, 0.05 mmol, 1.0 equiv.) and **N34** (1 mg, 10 mol%) gave **28a** (61%, >20:1 d.r.) as an oil. ^{1}H NMR (600 MHz, $CDCl_3$) δ 7.30 (2H, t, *J* = 7.5 Hz), 7.20 (1H, t, *J* = 7.4 Hz), 7.14 (2H, d, *J* = 7.5 Hz), 4.17 (1H, d, *J* = 10.1 Hz), 3.79 (1H, d, *J* = 9.0 Hz), 3.51 (1H, dd, *J* = 10.1, 6.8 Hz), 3.45 (1H, dd, *J* = 9.0, 4.5 Hz), 3.28 (1H, td, *J* = 7.7, 4.5 Hz), 2.99 (1H, d, *J* = 7.3 Hz), 2.41 (1H, t, *J* = 7.5 Hz), 1.11 (3H, s), 0.74 (3H, s); ^{13}C NMR (151 MHz, $CDCl_3$) δ 140.8, 128.2, 127.8, 126.1, 72.3, 69.3, 52.1, 46.8, 38.2, 37.5, 26.4, 24.4. Data in accordance with the literature.[19]

(1*R,5*R**,7*S**)-7-(4-Methoxyphenyl)-6,6-dimethyl-3-oxabicyclo[3.2.0]heptane (29a)**

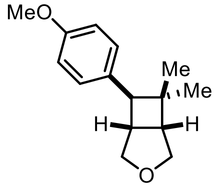

Following **GP8**, but using stock solutions, **29** (12 mg, 0.05 mmol, 1.0 equiv.) and **N34** (0.4 mg, 5 mol%) gave **29a** (47%, >20:1 d.r.) as an oil. ^{1}H NMR (600 MHz, $CDCl_3$) δ 7.09–7.03 (2H, m), 6.88–6.82 (2H, m), 4.16 (1H, dd, *J* = 10.1, 1.2 Hz), 3.80 (3H, s), 3.78 (1H, d, *J* = 8.8 Hz), 3.50 (1H, dd, *J* = 10.1, 6.7 Hz), 3.43 (1H, dd, *J* = 9.0, 4.5 Hz), 3.21 (1H, td, *J* = 7.7, 4.5 Hz), 2.91 (1H, d, *J* = 7.3 Hz), 2.39 (1H, ddd, *J* = 8.0, 6.7, 1.3 Hz), 1.08 (3H, s), 0.73 (3H, s); ^{13}C NMR (151 MHz, $CDCl_3$) δ 158.1, 132.9, 128.8, 113.6, 72.2, 69.3, 55.4, 51.4, 46.7, 38.6, 37.4, 26.4, 24.3. Data in accordance with the literature.[19]

(1*R,5*R**,7*S**)-6,6-Dimethyl-7-(4-(trifluoromethyl)phenyl)-3-oxabicyclo[3.2.0]heptane (30a)**

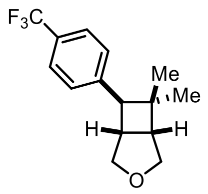

Following **GP8**, but using CH_2Cl_2 (2.5 mL, 0.02 M), **30** (14 mg, 0.05 mmol, 1.0 equiv.) and **N24** (1 mg, 10 mol%) gave **30a** (74%, 10:1 d.r.) as an oil. ^{1}H NMR (600 MHz, $CDCl_3$) δ 7.55 (2H, d, *J* = 8.1 Hz), 7.24 (2H, d, *J* = 8.0 Hz), 4.18 (1H, d, *J* = 10.1 Hz), 3.79 (1H, d, *J* = 9.1 Hz), 3.52 (1H, dd, *J* = 10.2, 6.7 Hz), 3.45 (1H, dd, *J* = 9.1, 4.4 Hz), 3.29 (1H, td, *J* = 7.7, 4.4 Hz), 3.04 (1H, d, *J* = 7.3 Hz), 2.43 (1H, t, *J* = 7.4 Hz), 1.13 (3H, s), 0.74 (3H, s); ^{13}C NMR (151 MHz, $CDCl_3$) δ 145.0, 128.4 (q, *J* = 32.3 Hz), 128.0, 125.1 (q, *J* = 3.7 Hz), 124.5 (q, *J* = 271.6 Hz), 72.1, 69.3, 51.9, 46.8, 38.1, 37.7, 26.4, 24.4; ^{19}F NMR (565 MHz, $CDCl_3$) δ –62.30 (s). Data in accordance with the literature.[34]

(1*S,5*R**,7*R**)-7-(Furan-2-yl)-6,6-dimethyl-3-oxabicyclo[3.2.0]heptane (31a)**

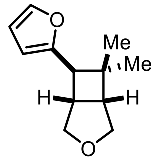

Following **GP8**, but using CH_2Cl_2 (2.5 mL, 0.02 M) and 6 h irradiation, **31** (10 mg, 0.05 mmol, 1.0 equiv.) and **N24** (1 mg, 10 mol%) gave **31a** (76%, 10:1 d.r.) as an oil. ^{1}H NMR (400 MHz, $CDCl_3$) δ 7.33 (1H, d, *J* = 1.9 Hz), 6.31 (1H, dd, *J* = 3.3, 1.9 Hz), 6.03 (1H, d, *J* = 3.1 Hz), 4.11 (1H, d, *J* = 10.1 Hz), 3.79 (1H, d, *J* = 9.1 Hz), 3.45 (1H, dd, *J* = 10.0, 6.6 Hz), 3.40 (1H, dd, *J* = 9.1, 4.5 Hz), 3.16 (1H, td, *J* = 7.5, 4.4 Hz), 2.86 (1H, d, *J* = 7.0 Hz), 2.40 (1H, t, *J* = 7.3 Hz), 1.06 (3H, s), 0.88 (3H, s); ^{13}C NMR (101 MHz, $CDCl_3$) δ 156.3, 141.4, 110.1, 105.8, 72.1, 69.3, 46.7, 46.0, 38.6, 37.8, 26.1, 23.8. Data in accordance with the literature.[19]

2-((1*R,5*R**,6*R**)-7,7-Dimethyl-3-oxabicyclo[3.2.0]heptan-6-yl)pyridine (32a)**

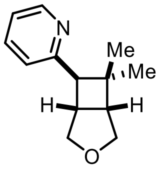

Following **GP8**, but using CH_2Cl_2 (2.5 mL, 0.02 M) and 48 h of irradiation, **32** (10 mg, 0.05 mmol, 1.0 equiv.) and **N24** (2 mg, 15 mol%) gave **32a** (40%, >20:1 d.r.) as an oil. ^{1}H NMR (600 MHz, $CDCl_3$) δ 8.57 (1H, ddd, *J* = 4.9, 2.0, 1.0 Hz), 7.57 (1H, td, *J* = 7.6, 1.9 Hz), 7.09 (1H, ddd, *J* = 7.5, 4.9, 1.2 Hz), 7.02 (1H, dt, *J* = 8.0, 1.2 Hz), 4.16 (1H, dd, *J* = 10.0, 1.1 Hz), 3.81 (1H, d, *J* = 9.1 Hz), 3.66 (1H, ddd, *J* = 8.1, 6.9, 4.6 Hz), 3.50 (1H, dd, *J* = 10.1, 6.6 Hz), 3.46 (1H, dd, *J* = 9.1, 4.6 Hz), 3.05 (1H, d, *J* = 6.9 Hz), 2.42 (1H, ddd, *J* = 8.0, 6.7, 1.3 Hz), 1.15 (3H, s), 0.73 (3H, s); ^{13}C NMR (151 MHz, $CDCl_3$) δ 160.6, 149.1, 135.8, 123.1, 121.1, 72.5, 69.3, 53.7, 46.7, 37.8, 36.7, 26.0, 24.4. Data in accordance with the literature.[19]

(1*R,5*R**,7*S**)-6,6-Dimethyl-7-phenyl-3-tosyl-3-azabicyclo[3.2.0]heptane (33a)**

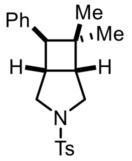

Following **GP8**, but using CH_2Cl_2 (2.5 mL, 0.02 M), **33** (17.8 mg, 0.05 mmol, 1.0 equiv.) and **N24** (1 mg, 10 mol%) gave **33a** (74%, >20:1 d.r.) as a solid. ^{1}H NMR (600 MHz, $CDCl_3$) δ 7.73 (2H, d, *J* = 8.3 Hz), 7.34 (2H, d, *J* = 7.9 Hz), 7.29 (2H, t, *J* = 7.6 Hz), 7.20 (1H, t, *J* = 7.4 Hz), 7.09 (2H, d, *J* = 7.8 Hz), 3.72 (1H, d, *J* = 10.6 Hz), 3.42 (1H, d, *J* = 9.4 Hz), 3.21 (1H, d, *J* = 7.5 Hz), 3.16 (1H, td, *J* = 7.7, 5.3 Hz), 2.64 (1H, dd, *J* = 10.6, 7.9 Hz), 2.61 (1H, dd, *J* = 9.5, 5.4 Hz), 2.45 (3H, s), 2.28 (1H, t, *J* = 7.9 Hz), 1.17 (3H, s), 0.70 (3H, s); ^{13}C NMR (151 MHz, $CDCl_3$) δ 143.7, 140.1, 132.2, 129.7, 128.3, 128.2, 127.8, 126.3, 53.1, 52.0, 49.2, 45.2, 37.9, 36.5, 26.3, 24.4, 21.7. Data in accordance with the literature.[19]

(±)-Ethyl Cannabiorcicyclolic Ester (34a)

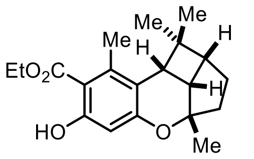

Following **GP8**, but a stock solution of **34** (33 mg, 0.1 mmol, 1.0 equiv.) and **N34** (2 mg, 10 mol%) in CH_2Cl_2 (10 mL) was prepared and a 2 mL aliquote used in the reaction gave **34a** (56%, >20:1 d.r.) as a solid. ^{1}H NMR ($CDCl_3$, 600 MHz) δ 11.91 (1H, s), 6.25 (1H, s), 4.38 (2H, q, *J* = 7.1 Hz), 3.12 (1H, d, *J* = 9.6 Hz), 2.57 (1H, dd, *J* = 9.3, 7.6 Hz), 2.48 (3H, s), 2.41 (1H, t, *J* = 7.4 Hz), 1.92 (1H, td, *J* = 12.7, 7.6 Hz), 1.71–1.63 (2H, m), 1.62–1.55 (1H, m), 1.40 (3H, t, *J* = 7.1 Hz), 1.39 (6H, s), 0.78 (3H, s); ^{13}C NMR ($CDCl_3$, 151 MHz) δ 172.3, 163.6, 157.9, 140.3, 113.2, 109.8, 104.7, 84.3, 61.1, 46.7, 39.3, 38.7, 37.7, 36.1, 33.8, 27.8, 25.9, 24.4, 17.8, 14.4. Data in accordance with the literature.[19]

Methyl (1*S,2*S**,3*R**)-2,3-Diphenylcyclobutane-1-carboxylate (36)**

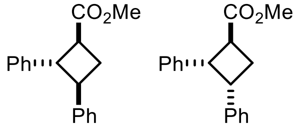

cis-**36** *trans*-**36**

Following the procedure described in **Section 11.1**, **(*E*)-14** (16 mg, 0.1 mmol, 1.0 equiv.), styrene (115µL, 1.0 mmol, 10.0 equiv.), and **N24** (4 mg, 20 mol%) in THF (1.0 mL), gave **36** (76%, 2.2:1 d.r.).

cis-**36**: ^{1}H NMR ($CDCl_3$, 600 MHz): δ 7.35–7.19 (10H, m), 3.86 (1H, t, *J* = 9.8 Hz), 3.73 (3H, s), 3.54 (1H, td, *J* = 10.1, 8.2 Hz), 3.21 (1H, td, *J* = 9.8, 8.2 Hz), 2.64 (1H, dtd, *J* = 10.8, 8.3, 0.8 Hz), 2.42 (1H, q, *J* = 10.4 Hz); ^{13}C NMR ($CDCl_3$, 151 MHz): δ 174.7, 143.3, 142.3, 128.63, 128.62, 127.0, 126.9, 126.8, 126.7, 52.0, 50.9, 43.6, 41.6, 29.7. Data in accordance with the literature.[35]

trans-**36**: R_f 0.52 [pentane:EtOAc (30:1)]; ^{1}H NMR ($CDCl_3$, 600 MHz): δ 7.13–7.00 (6H, m), 7.00–6.95 (2H, m), 6.92–6.88 (2H, m), 4.29 (1H, t, J = 9.3 Hz), 3.99 (1H, td, J = 9.3, 4.1 Hz), 3.72 (3H, s), 3.69 (1H, td, J = 8.9, 1.1 Hz), 2.82 (1H, dddd, J = 12.1, 9.1, 8.6, 0.5 Hz), 2.59 (1H, dddd, J = 12.1, 9.3, 4.1, 0.9 Hz); ^{13}C NMR ($CDCl_3$, 151 MHz): δ 175.3, 140.5, 139.3, 128.2, 128.1, 127.9, 127.7, 126.2, 126.1, 52.0, 47.7, 42.4, 40.8, 27.2.; HRMS (ESI) found $[M+Na]^+$ 289.11965, $C_{16}H_{20}O_2Na$ requires 289.11977.

(1*R,2*R**,3*S**)-3-Methyl-2-phenyl-3-(prop-1-en-2-yl)cyclobutan-1-ol (S8)**

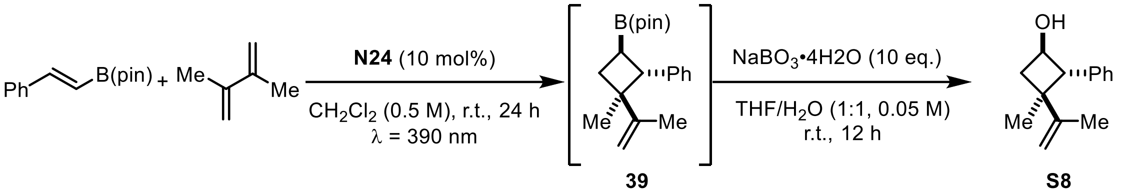

An oven-dried microwave vial containing a stir-bar was charged with the (*E*)-4,4,5,5-tetramethyl-2-styryl-1,32-dioxaborolane (115 mg, 0.50 mmol, 1 equiv.) and **N24** (9 mg, 10 mol%). The vial was capped with a Supelco aluminium crimp seal with septum (PTFE/butyl) and was evacuated and refilled with Ar (x 3). Dry and degassed CH_2Cl_2 (1.0 mL, 0.5 M) was added and the reaction mixture was stirred (> 500 rpm) under irradiation with a 390 nm Kessil LED lamp (5 cm distance, 100% intensity, fan on) for 24 h. NMR analysis of the crude mixture with 1,3-dinitrobenzene as an internal standard provided the NMR yield of [2+2] cycloaddition product (**39**) and the diastereomeric ratio. Otherwise, solvent was removed under reduced pressure and the crude residue dissolved in THF (5 mL). Sodium perborate tetrahydrate (769 mg, 5.00 mmol, 10 equiv.) and H_2O (5.0 mL) were added sequentially. The reaction mixture was stirred at r.t. After 12 h, the mixture was diluted with H_2O (30 mL) and EtOAc (20 mL). The phases were separated, and the aqueous layer extracted with EtOAc (2 x 20 mL). The combined organic layers were dried ($MgSO_4$), filtered and evaporated. The crude residue was purified by flash column chromatography to give **S8** (15 mg, 15%) as an oil. ^{1}H NMR ($CDCl_3$, 600 MHz) δ 7.32–7.28 (2H, m), 7.27–7.24 (2H, m), 7.21 (1H, tt, J = 7.2, 1.4 Hz), 4.82 (1H, s), 4.77 (1H, t, J = 1.5 Hz), 4.54–4.46 (1H, m), 3.39 (1H, d, J = 8.2 Hz), 2.22 (1H, dd, J = 10.7, 7.1 Hz), 1.96 (1H, dd, J = 10.7, 7.9 Hz), 1.72 (3H, t, J = 0.9 Hz), 0.96 (3H, s); ^{13}C NMR (151 MHz, CDCl3) δ 153.3, 139.5, 128.3, 128.2, 126.5, 108.4, 66.9, 57.6, 41.8, 40.6, 21.9, 19.1. Data in accordance with the literature.[36]

Dimethyl (1*R,2*R**,3*S**,4*S**)-3,4-Diphenylcyclobutane-1,2-dicarboxylate (40)**

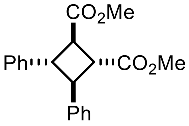

Following **GP8**, ***E*-14** (32 mg, 0.2 mmol, 1.0 equiv.) and **N24** (2 mg, 10 mol%) in CH_3CN (1.0 mL, 0.1 M), gave **40** (34%, >10:1 d.r.) as an oil. ^{1}H NMR (400 MHz, $CDCl_3$) δ 7.32–7.23 (8H, m), 7.23–7.18 (2H, m), 3.77–3.66 (2H, m), 3.70 (6H, s), 3.53–3.42 (2H, m); ^{13}C NMR (101 MHz, $CDCl_3$) δ 173.1, 141.1, 128.8, 127.3, 127.0, 52.3, 47.5, 44.6. Data in accordance with the literature.[37]

(1*R,5*S**,7*S**)-6-(Benzyloxy)-7-phenyl-3-oxa-6-azabicyclo[3.2.0]heptane (42)**

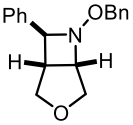

Following the procedure described in **Section 11.2**, **41** (14 mg, 0.05 mmol 1 equiv.) and **N24** (1 mg, 10 mol%) gave **42** (59%) as an oil. ^{1}H NMR ($CDCl_3$, 400 MHz) δ 7.35–7.12 (10H, m), 4.67 (1H, d, *J* = 10.6 Hz), 4.54 (2H, s), 4.40 (1H, d, *J* = 5.8 Hz), 4.21 (1H, t, *J* = 5.5 Hz), 3.86 (1H, d, *J* = 9.4 Hz), 3.42–3.34 (2H, m), 2.56 (1H, dt, *J* = 5.8, 3.6 Hz); ^{13}C NMR ($CDCl_3$, 101 MHz) δ 141.9, 138.4, 128.6, 128.5, 128.3, 127.7, 127.5, 126.5, 75.6, 74.1, 70.8, 68.5, 67.5, 42.6. Data in accordance with the literature.[23]

1-([1,1'-Biphenyl]-4-ylmethyl)-2-(cyanomethyl)-2-phenylcyclopropane-1-carbonitrile (44)

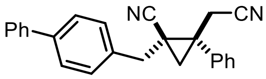

cis-**44**

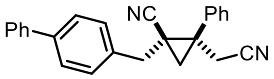

trans-**44**

Following the procedure described in **Section 11.3**, **43** (17 mg, 0.05 mmol, 1.0 equiv.) and **N36** (0.8 mg, 10 mol%) in EtOH (1.0 mL), gave **44** (78%, 1:1 d.r.).

cis-**44**: ^{1}H NMR ($CDCl_3$, 400 MHz) δ 7.59–7.51 (4H, m), 7.50–7.39 (7H, m), 7.37–7.30 (1H, m), 7.23–7.15 (2H, m), 3.14 (1H, dd, *J* = 17.1, 1.2 Hz), 3.02 (1H, d, *J* = 14.9 Hz), 2.86 (1H, d, *J* = 17.1 Hz), 1.96 (1H, d, *J* = 14.9 Hz), 1.75 (1H, dd, *J* = 6.3, 1.2 Hz), 1.68 (1H, dd, *J* = 6.2, 1.1 Hz). ^{13}C NMR ($CDCl_3$, 101 MHz) δ 140.7, 140.1, 135.4, 135.2, 129.73, 129.66, 129.36, 129.35, 129.0, 127.7, 127.6, 127.2, 120.9, 116.7, 38.2, 34.4, 29.0, 24.7, 23.4.

trans-**44**: ^{1}H NMR ($CDCl_3$, 400 MHz) δ 7.67–7.61 (4H, m), 7.52–7.32 (10H, m), 3.28 (1H, d, *J* = 15.2 Hz), 3.18 (1H, d, *J* = 15.2 Hz), 2.85 (2H, s), 2.03 (1H, d, *J* = 6.2 Hz), 1.52 (1H, d, *J* = 6.6 Hz); ^{13}C NMR ($CDCl_3$, 101 MHz) δ 140.9, 140.6, 137.8, 135.3, 129.5, 129.3 (2 x C), 129.0, 128.0, 127.7, 127.3, 120.6, 116.9, 36.8, 34.2, 25.9, 25.3, 23.6. Data in accordance with the literature.[24]

Supplementary Note 13: EnT Reactivities of Unactivated Substrates

Alkyl-Substituted Acrylates

We tested two acyclic acrylates **S11** and **S12** (both lacking the aromatic group in conjugation with the alkene) and in both cases we did not observe any *E/Z* isomerization (Supplementary Figure 21).

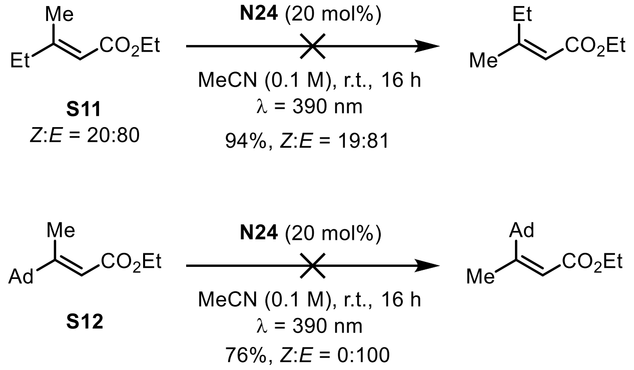

Supplementary Figure 21. Examples of alkyl acrylates examined under nitroarene catalyzed isomerization conditions.

To better understand these results, we have performed additional computational studies on **S11**. The reaction outcomes are consistent with the calculated triplet energies of the *E*- and *Z*-isomers of **S11** which are higher than the triplet energy of **N24** (compare entries 1 and 2 with entry 3 in Supplementary Table 8).

Supplementary Table 8. Adiabatic triplet energies of both *E*- and Z-isomer of **S11** and **N24** for comparison.

Entry	Species	E_T (kcal/mol)
1	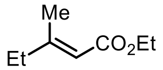	62.0
2	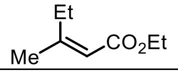	62.2
3	**N24**	59.6

Supplementary Note 14: NMR Spectra

N22 – ^{1}H NMR (400 MHz, $CDCl_3$)

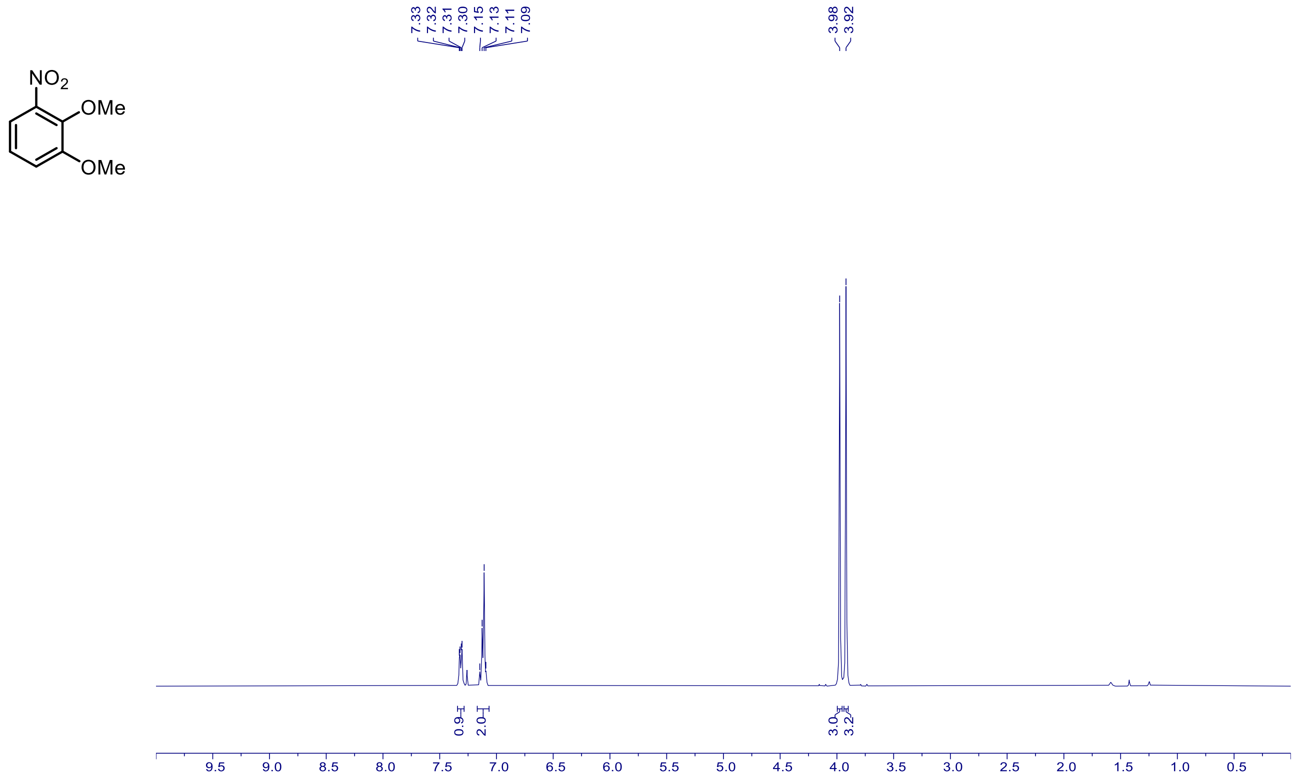

N22 – ^{13}C NMR (101 MHz, $CDCl_3$)

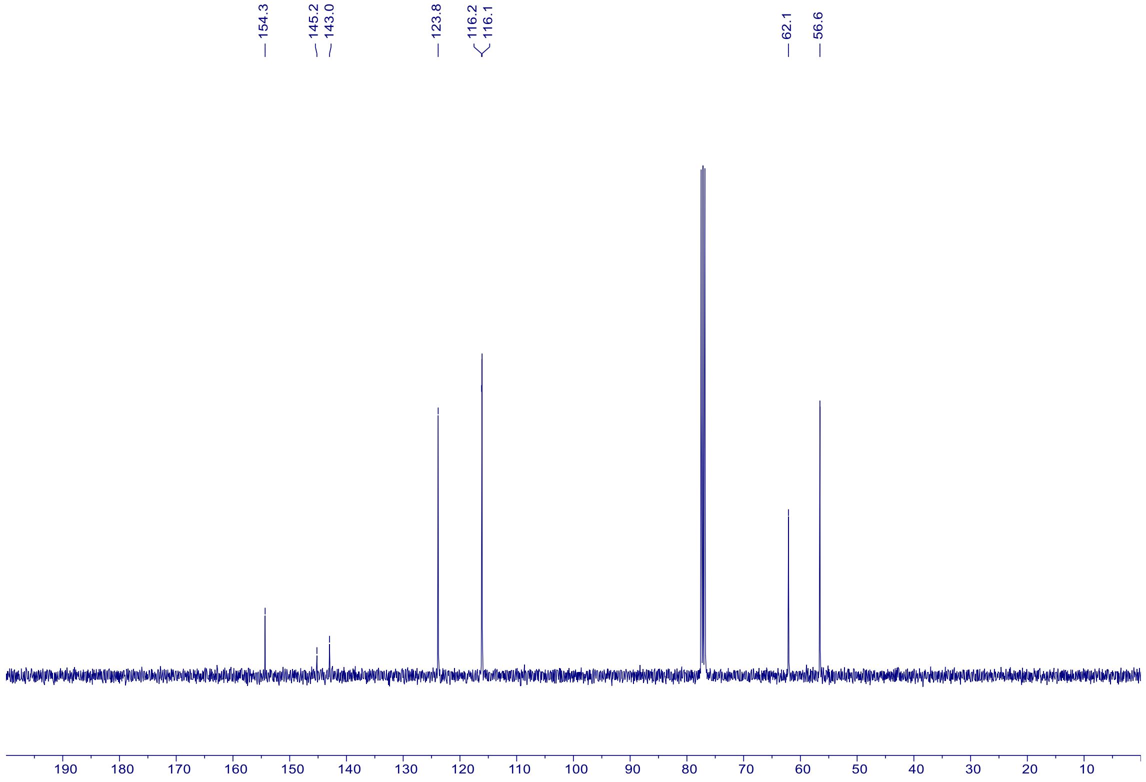

S1 – ^{1}H NMR (400 MHz, DMSO-d_6)

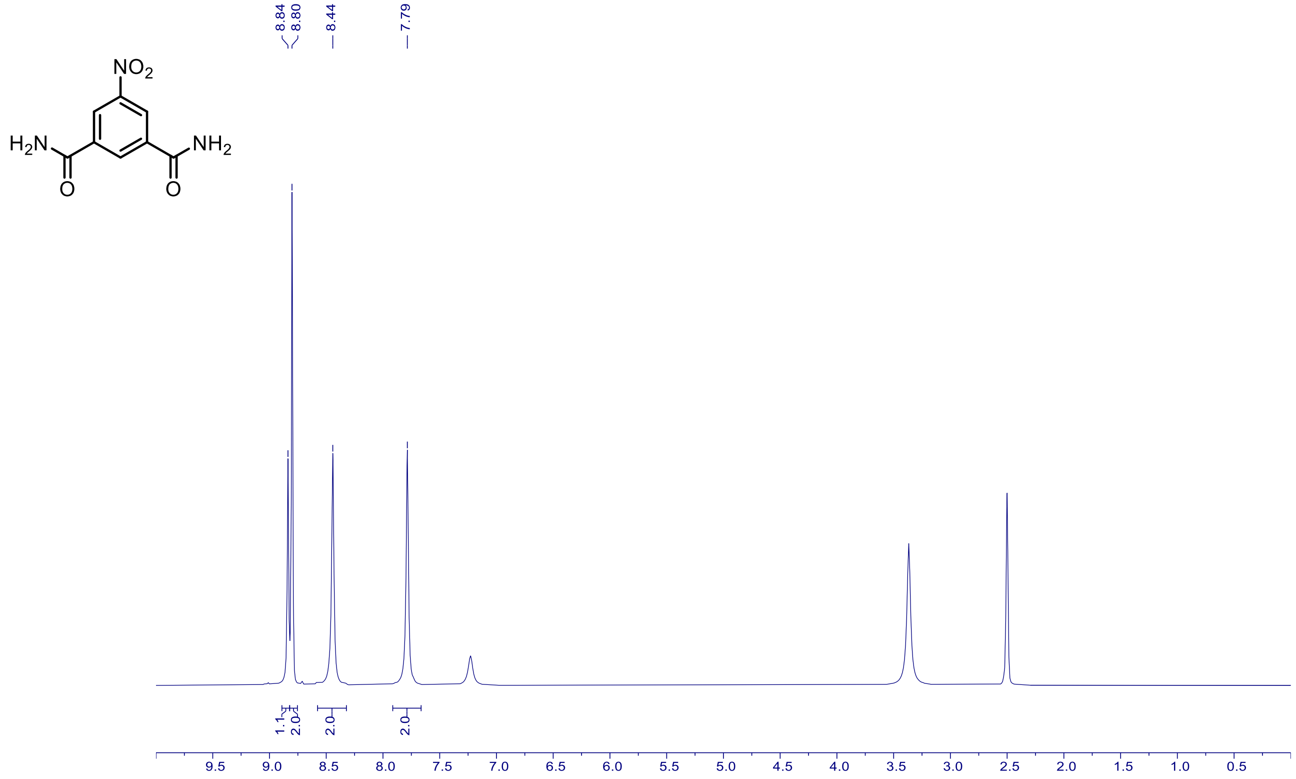

S1 – ^{13}C NMR (101 MHz, DMSO-d_6)

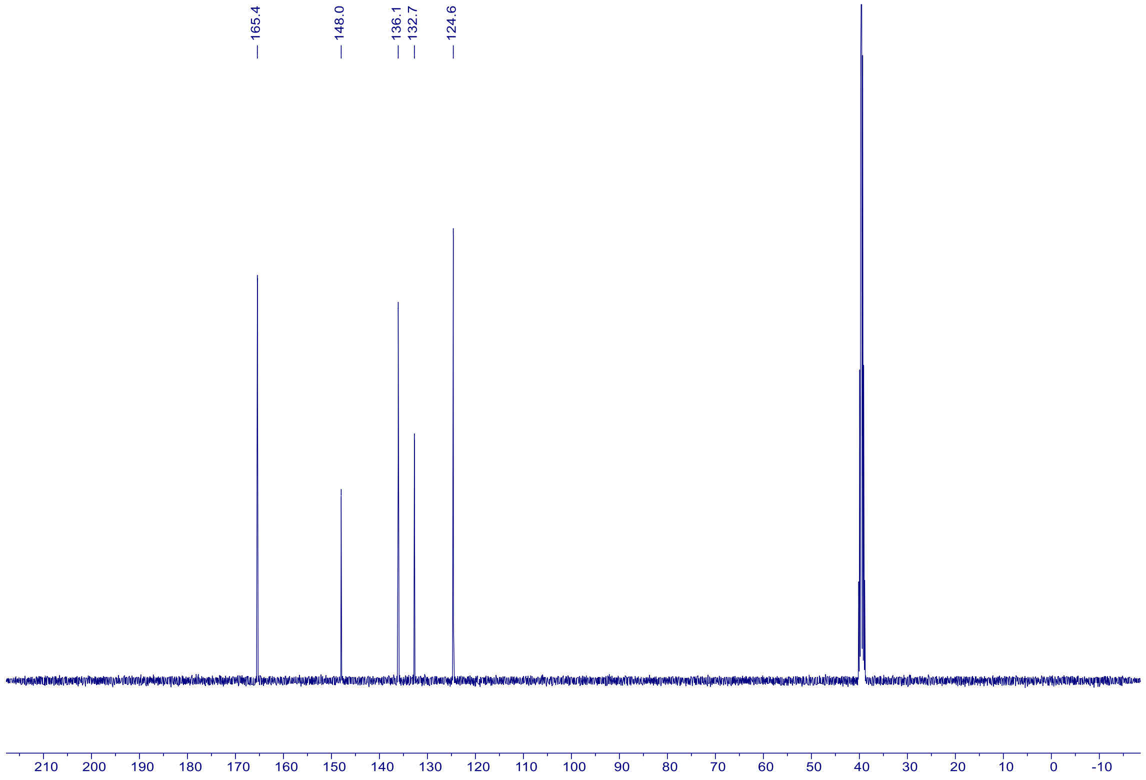

***E*-7** – ^{1}H NMR (600 MHz, $CDCl_3$)

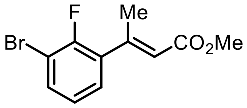

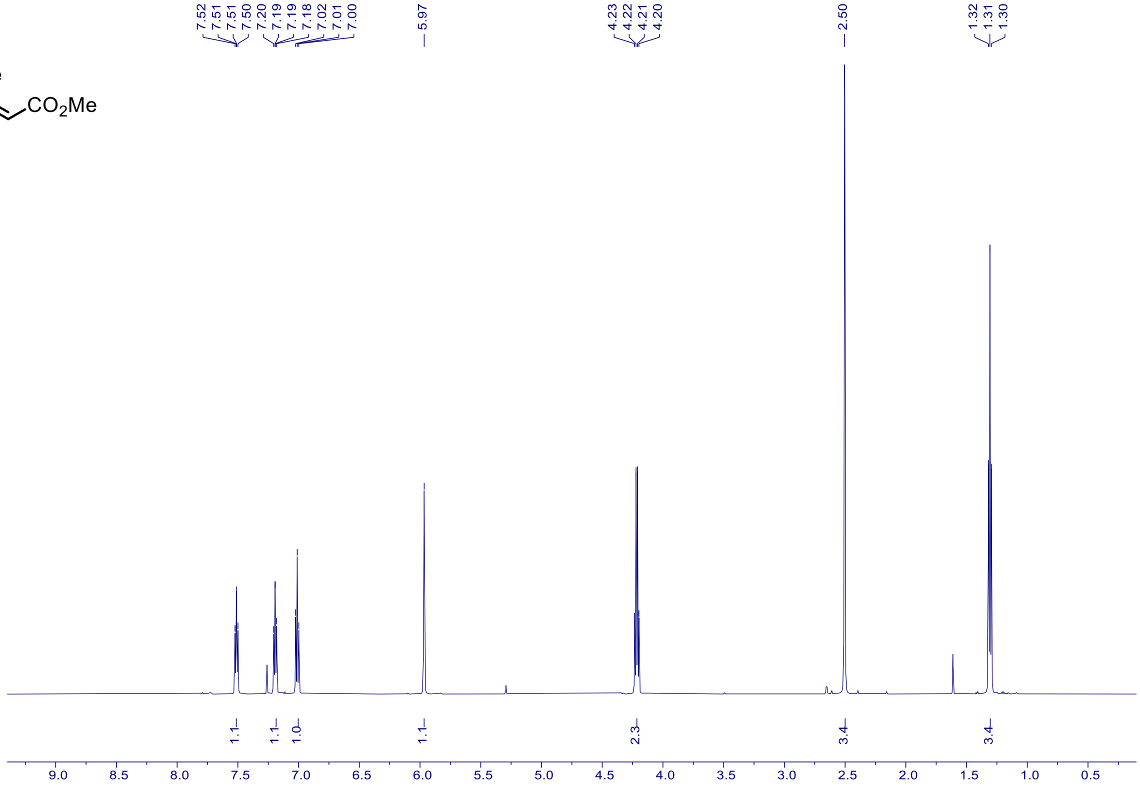

***E*-7** – ^{13}C NMR (151 MHz, $CDCl_3$)

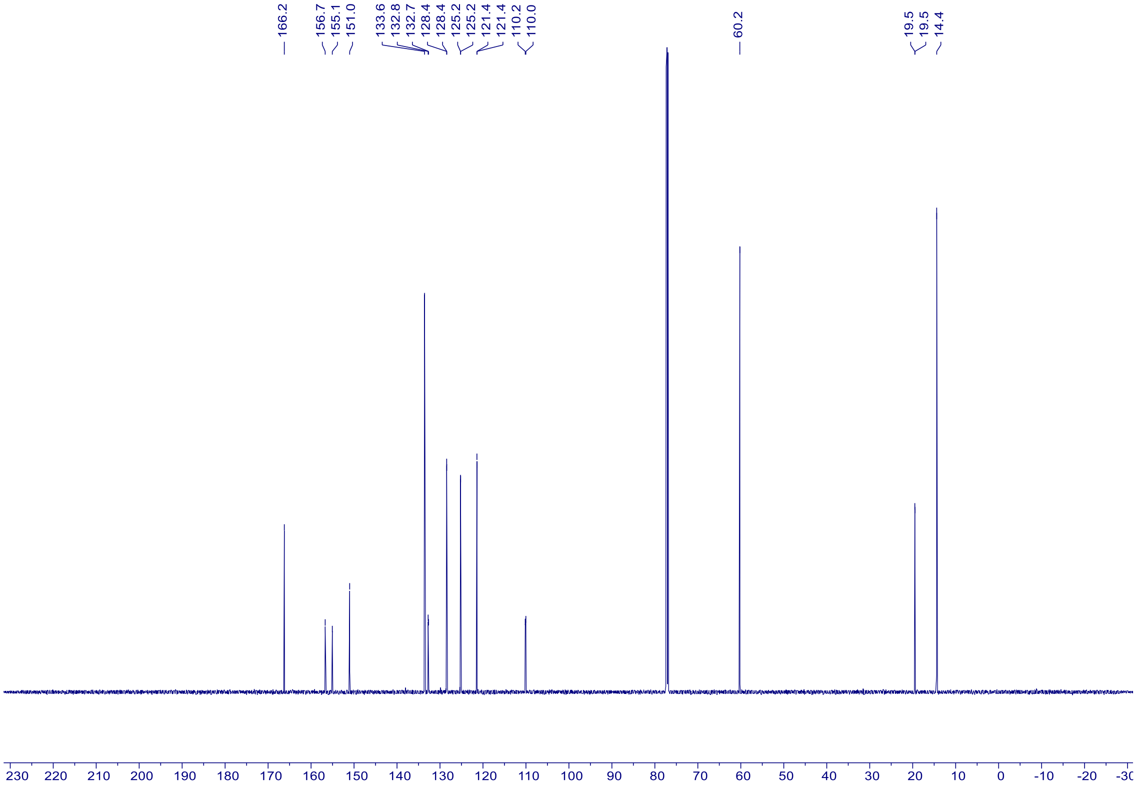

***E*-7** – ^{19}F NMR (564 MHz, $CDCl_3$)

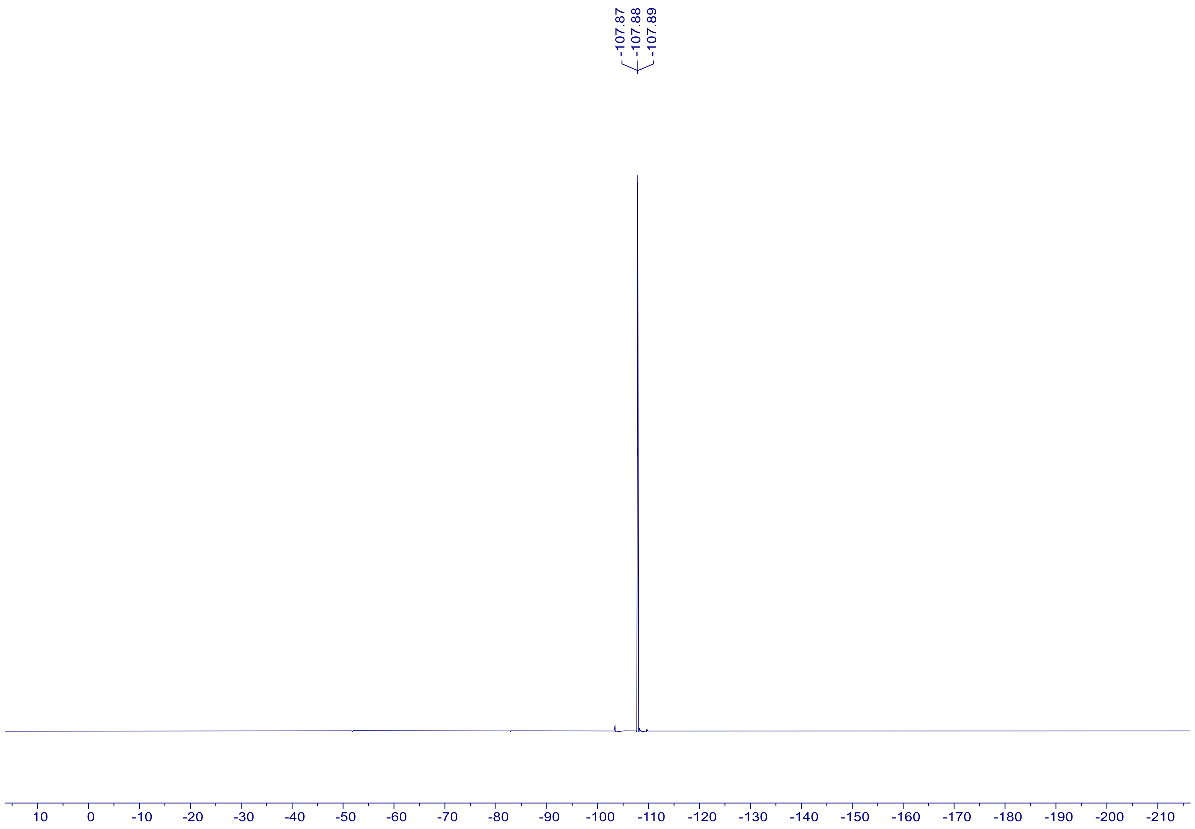

***E*-22** – ^{1}H NMR (400 MHz, MeOD-d_4)

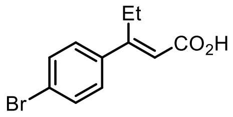

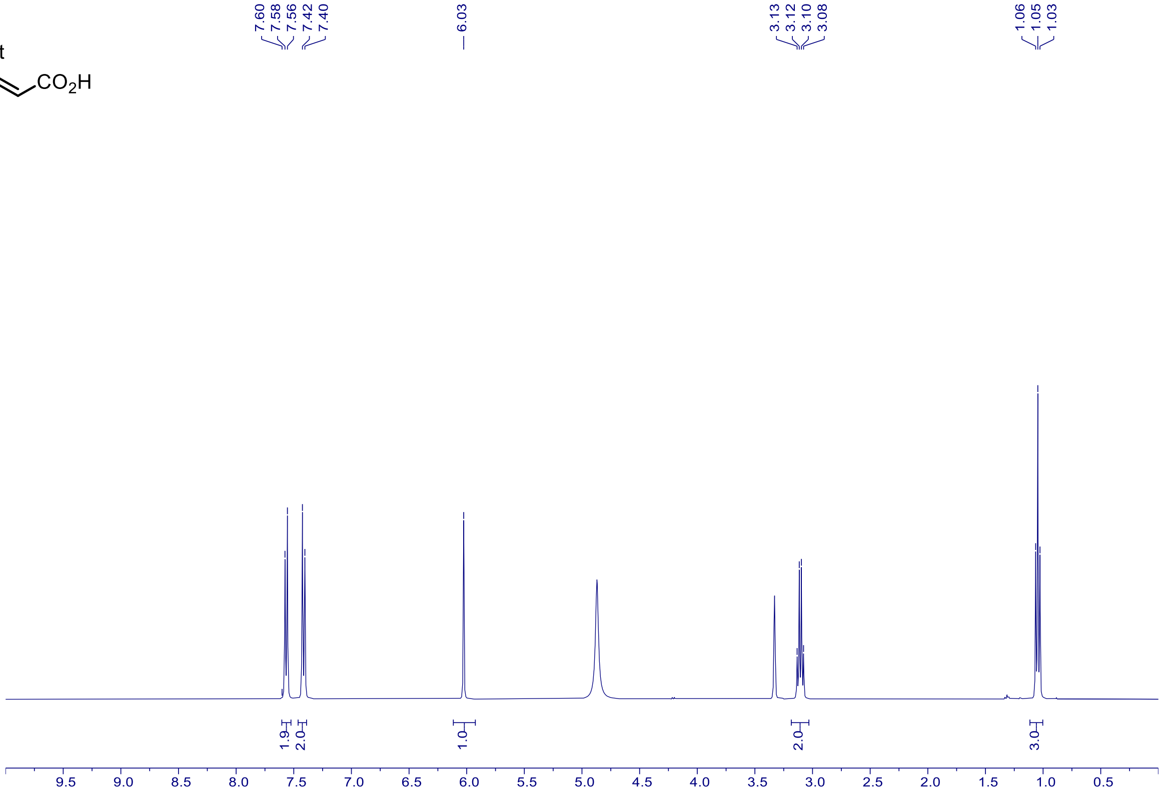

***E*-22** – ^{13}C NMR (101 MHz, MeOD-d_4)

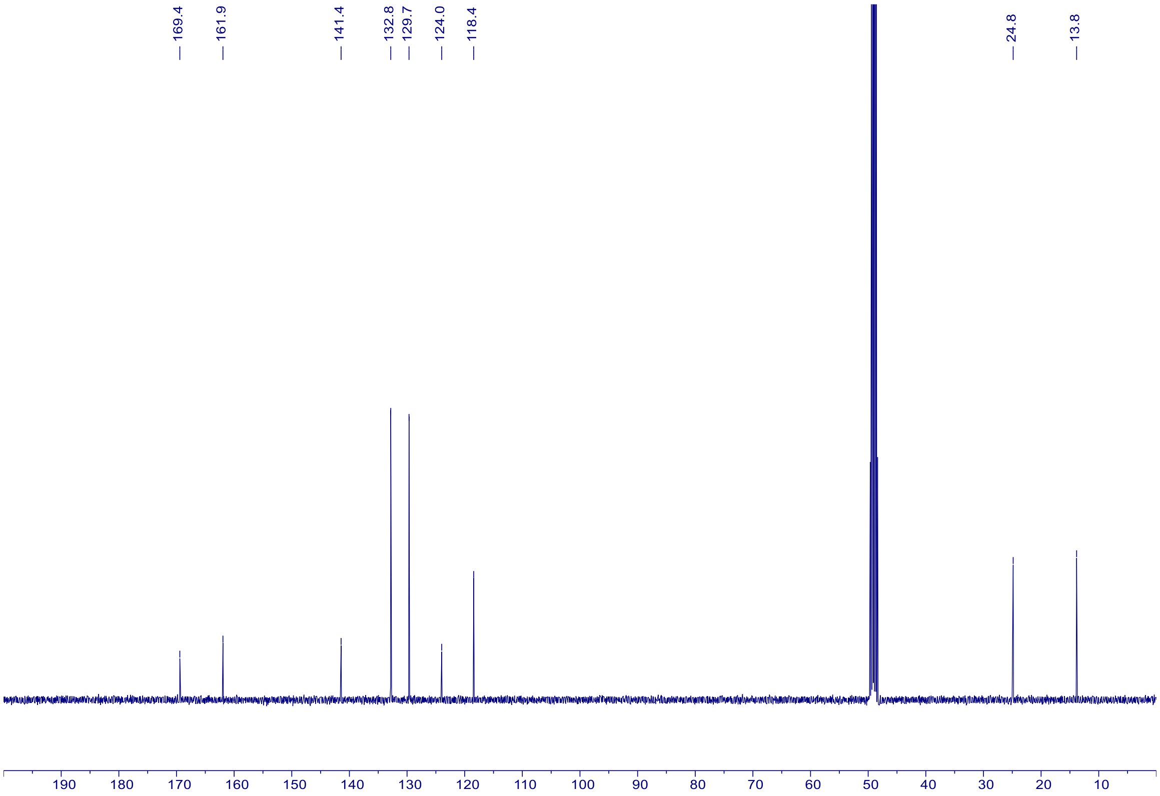

***E*-23** – ^{1}H NMR (600 MHz, MeOD-d_4)

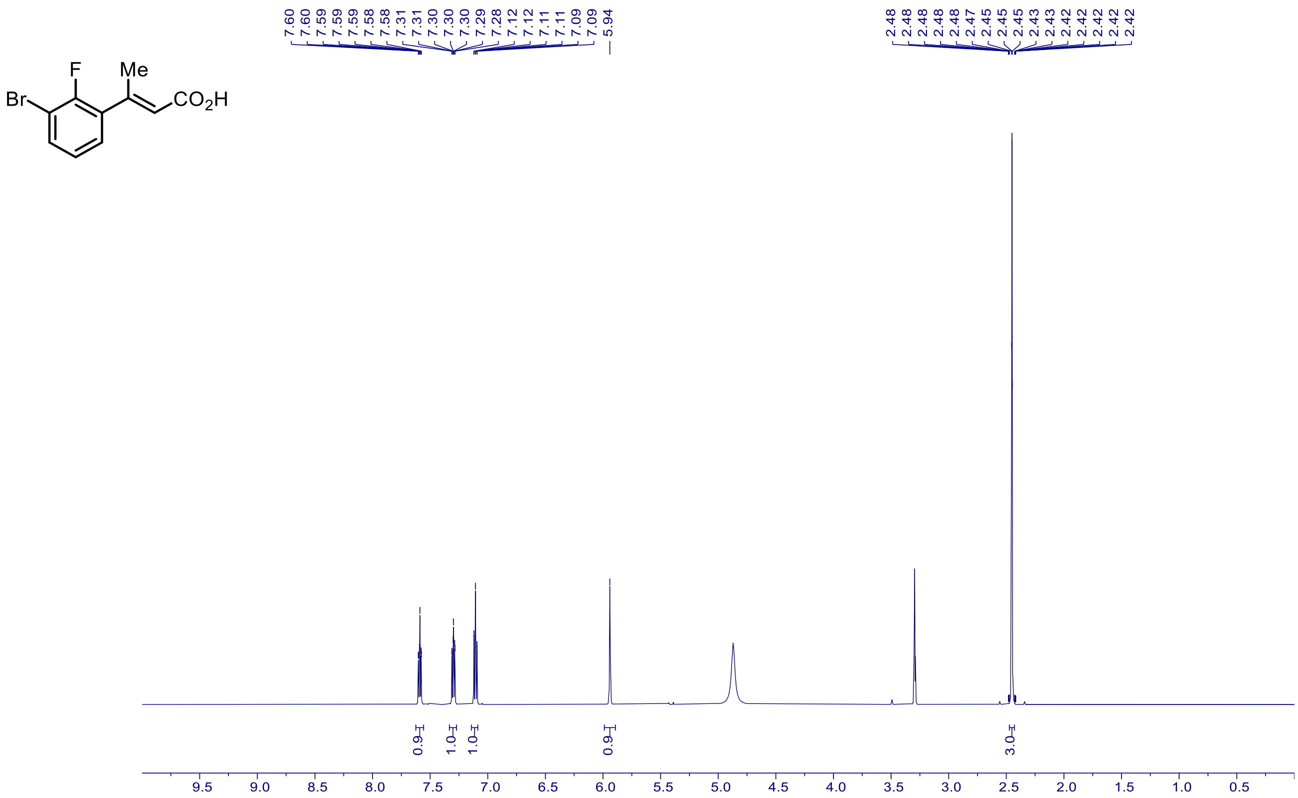

***E*-23** – ^{13}C NMR (151 MHz, MeOD-d_4)

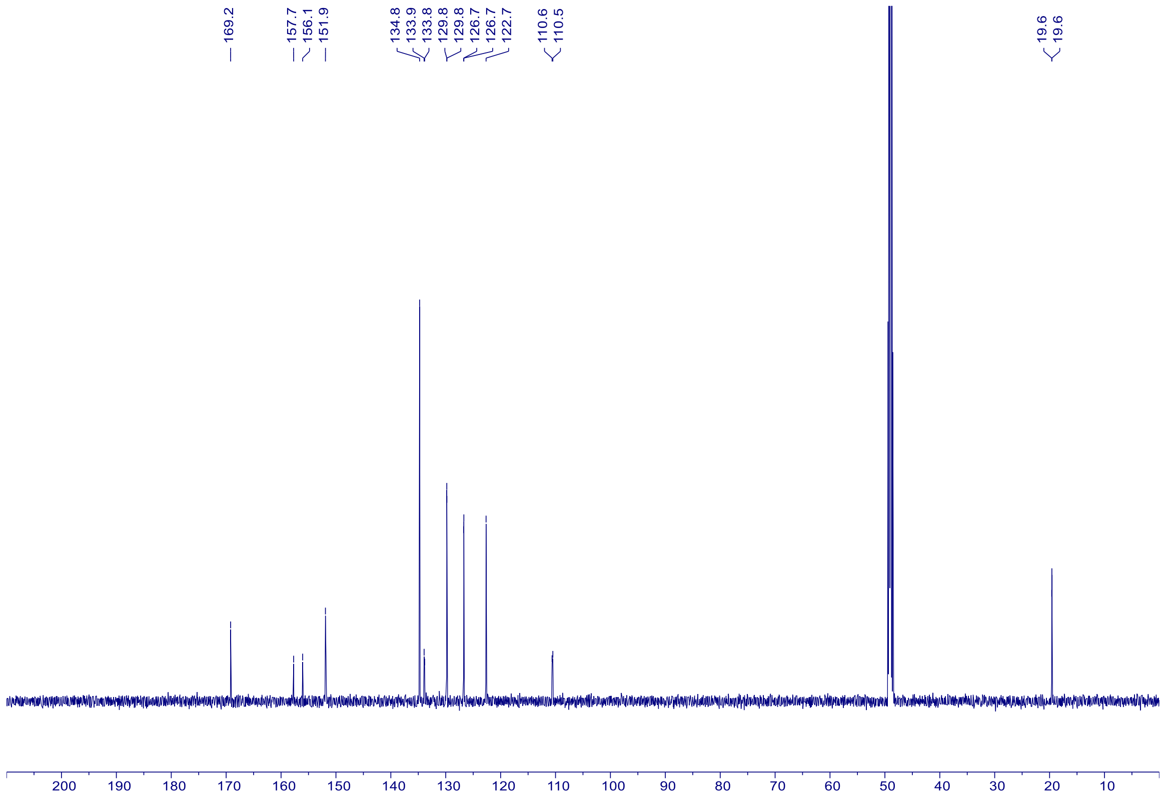

***E*-23** – ^{19}F NMR (564 MHz, MeOD-d_4)

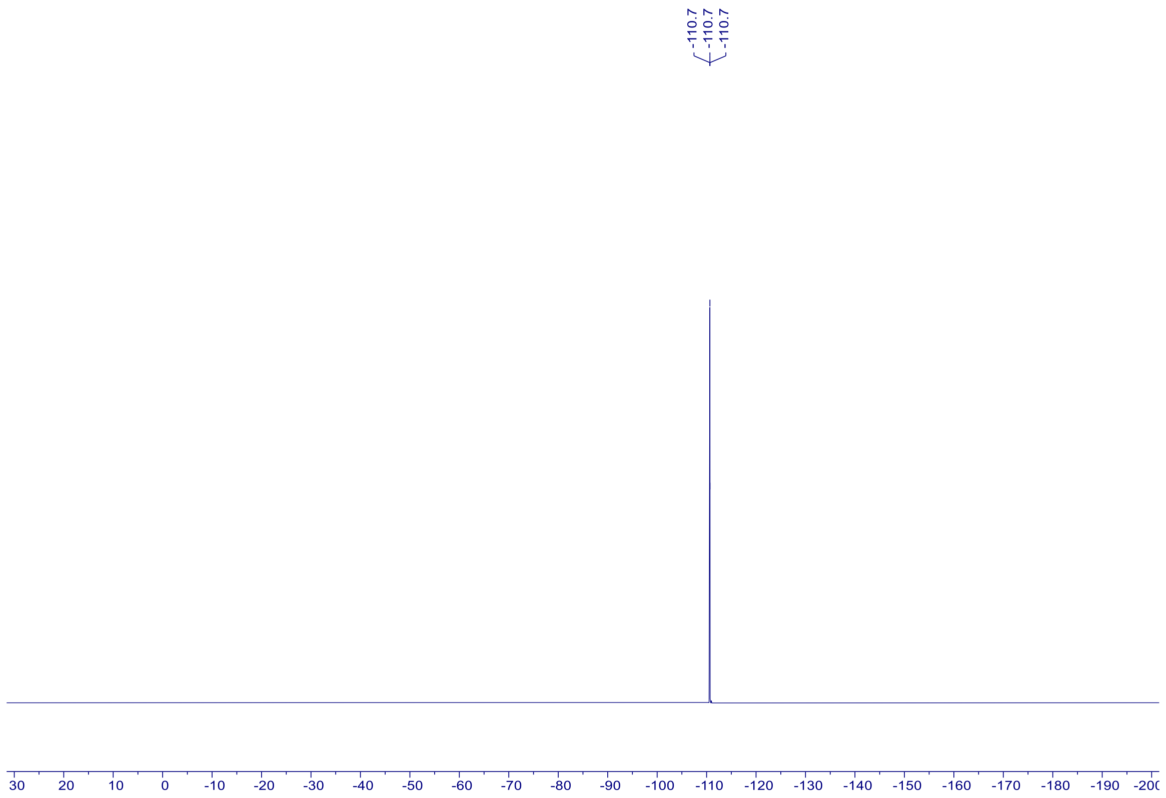

***Z*-7** – ^{1}H NMR (600 MHz, $CDCl_3$)

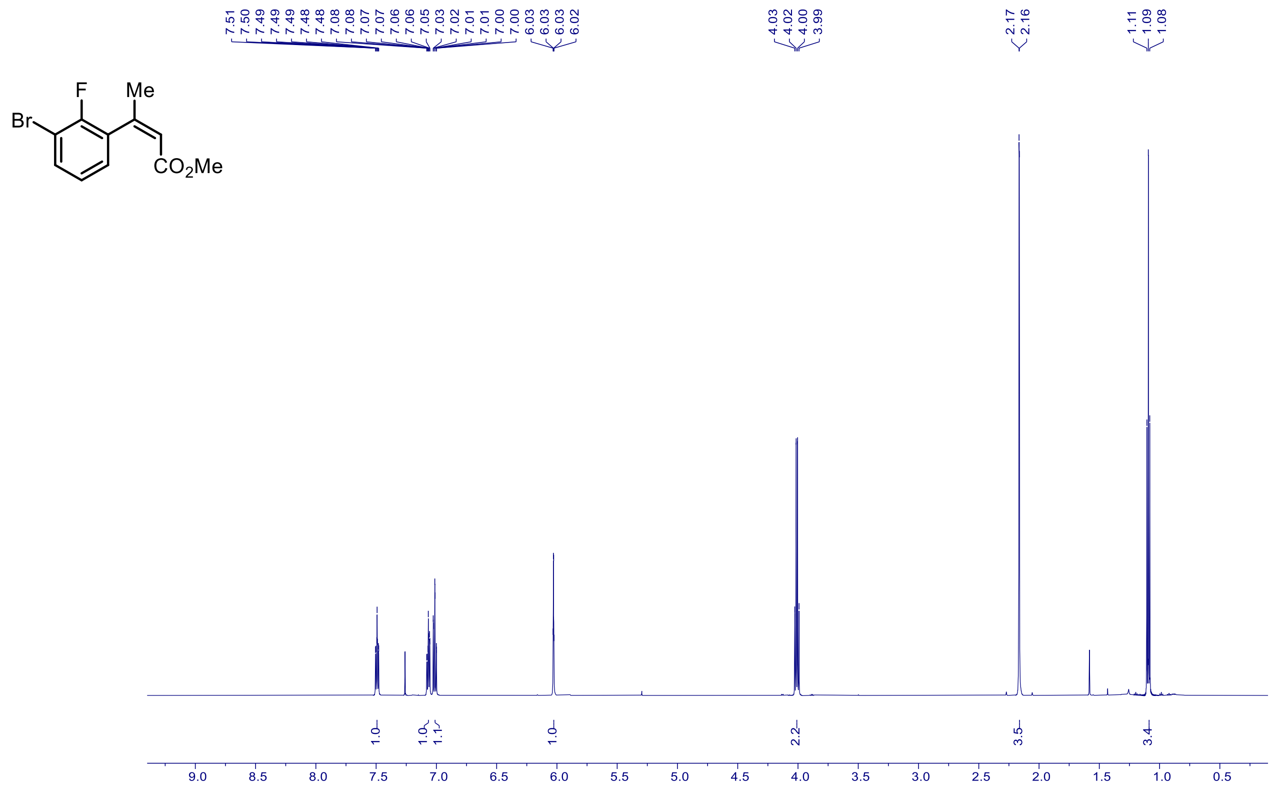

***Z*-7**– ^{13}C NMR (151 MHz, $CDCl_3$)

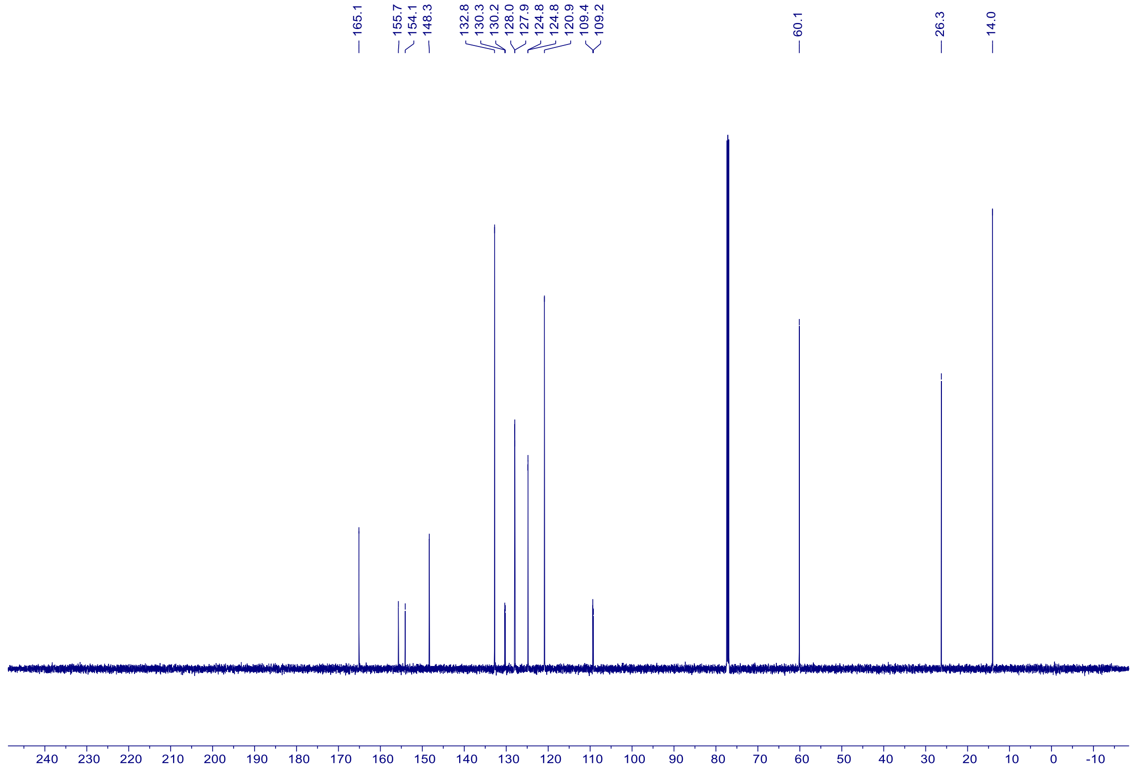

***Z*-7** – ^{19}F NMR (564 MHz, $CDCl_3$)

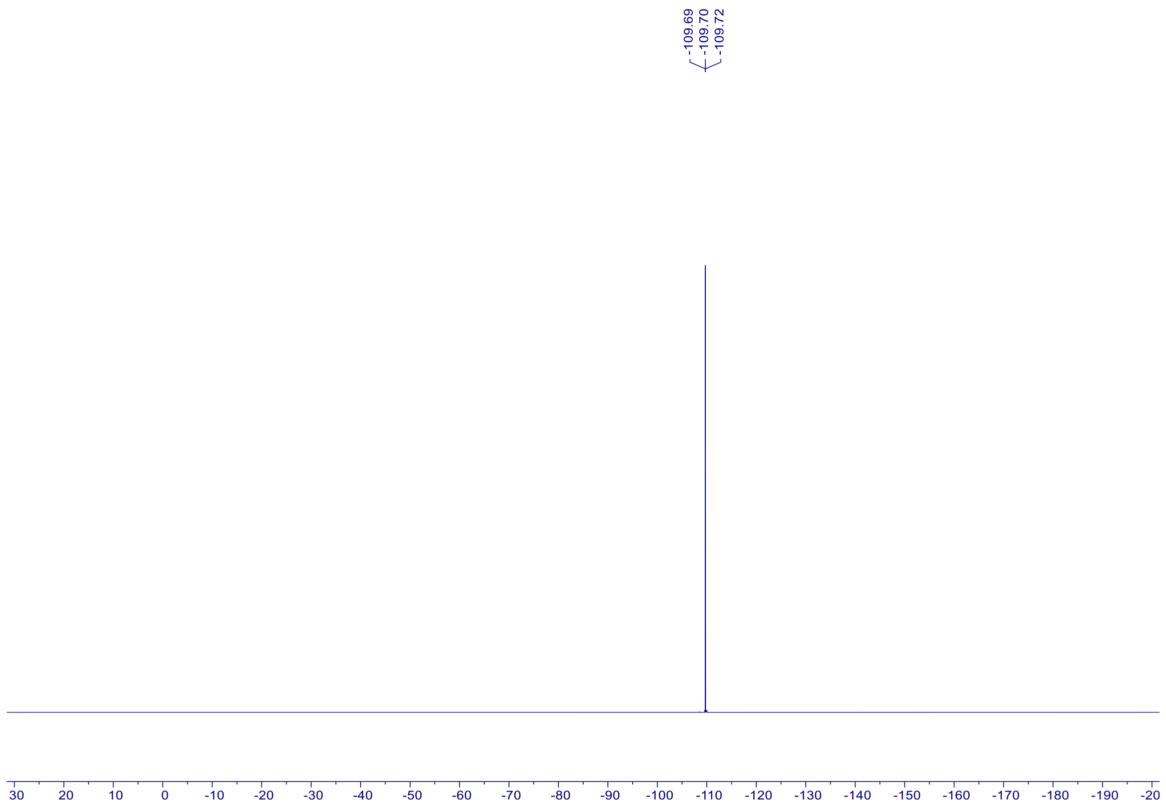

***Z*-12** – ^{1}H NMR (600 MHz, $CDCl_3$)

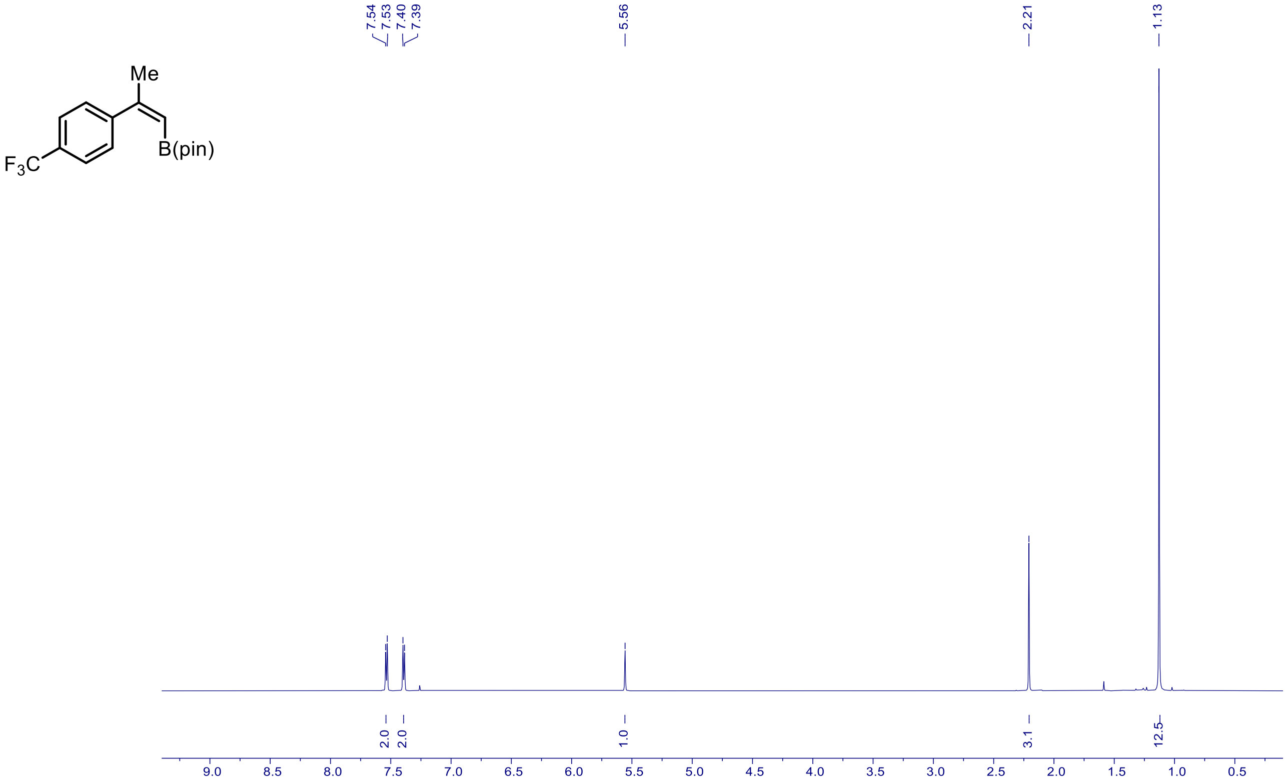

***Z*-12** – ^{13}C NMR (151 MHz, $CDCl_3$)

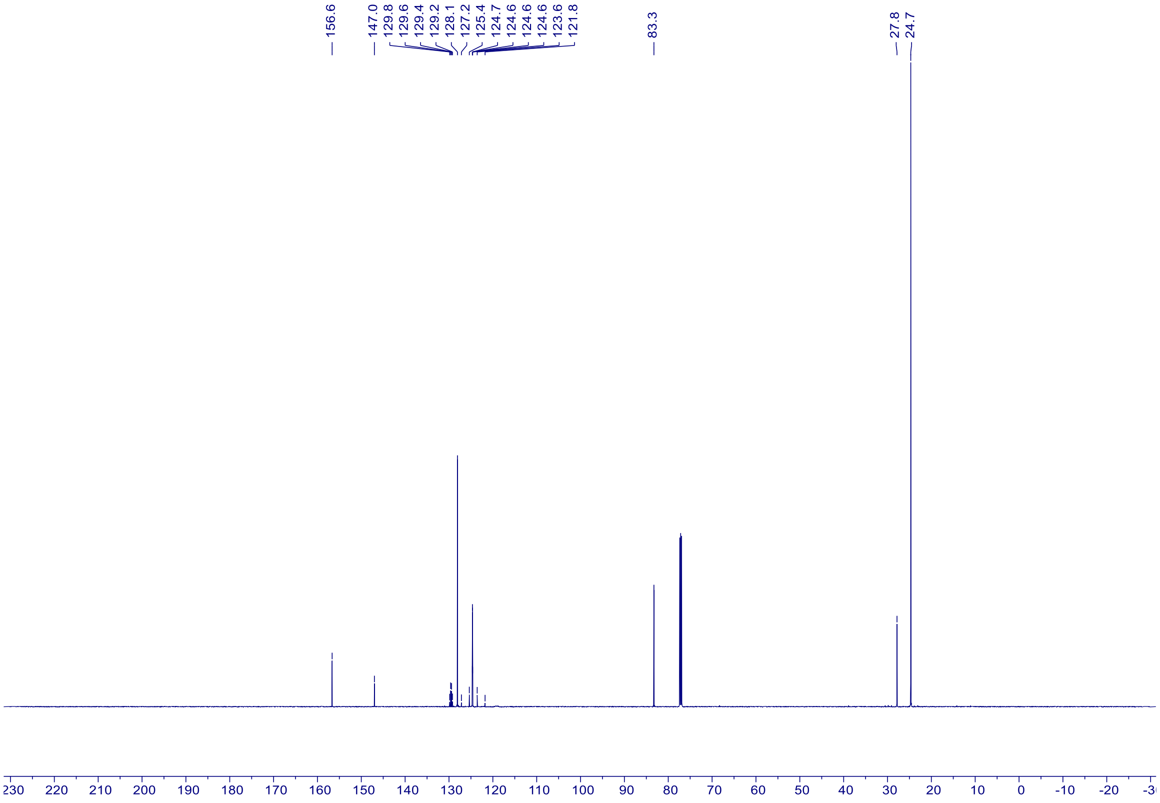

***Z*-12** – ^{11}B NMR (193 MHz, $CDCl_3$)

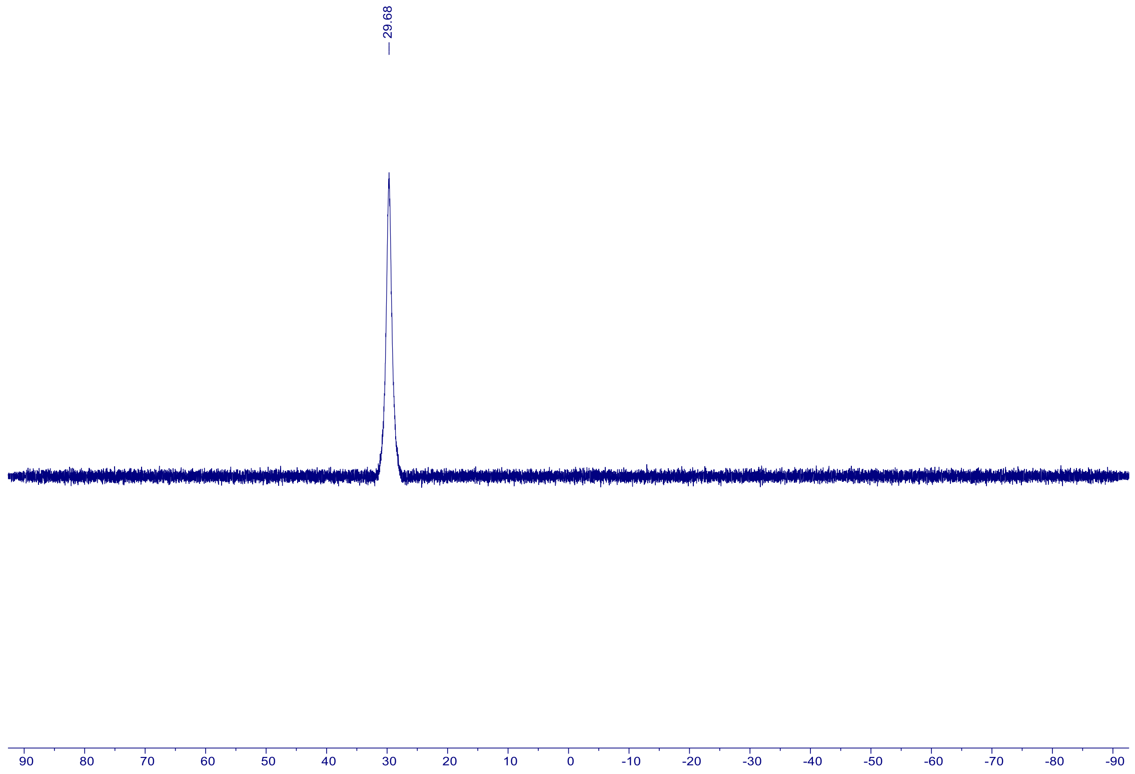

***Z*-12** – ^{19}F NMR (564 MHz, $CDCl_3$)

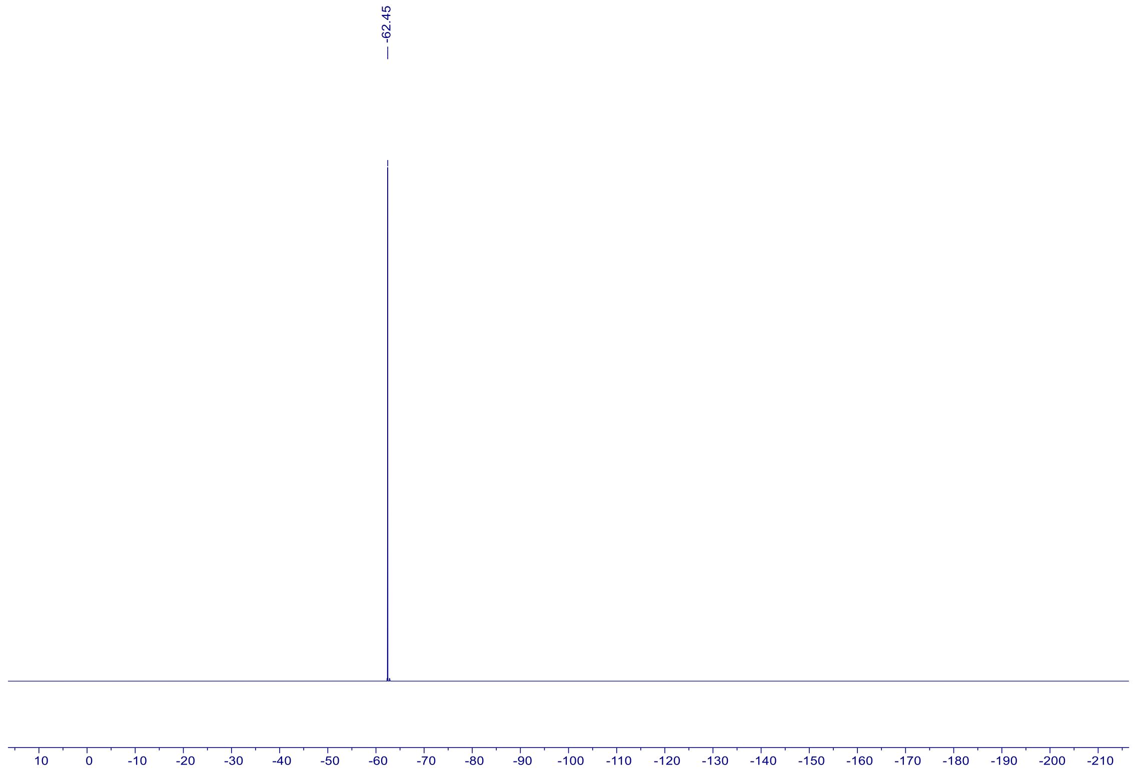

***Z*-22** – ^{1}H NMR (400 MHz, MeOD-d_4)

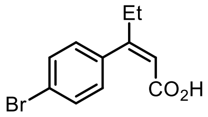

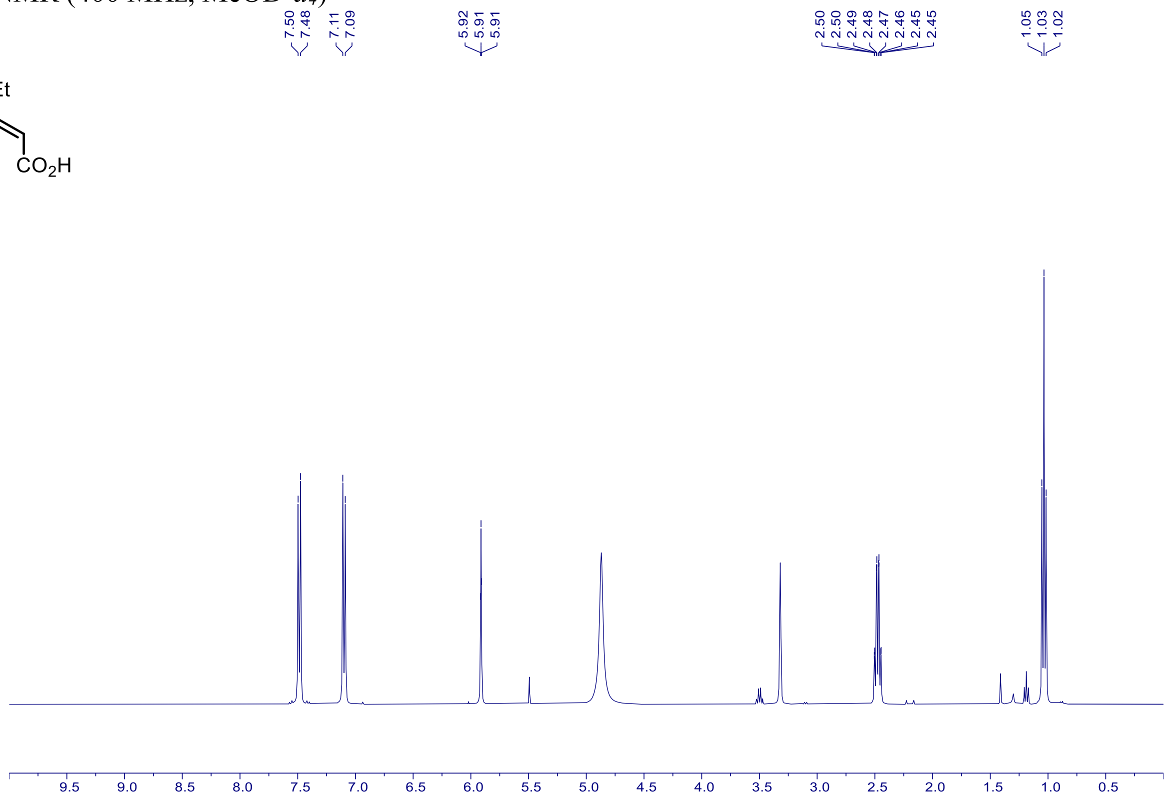

***Z*-22** – ^{13}C NMR (101 MHz, MeOD-d_4)

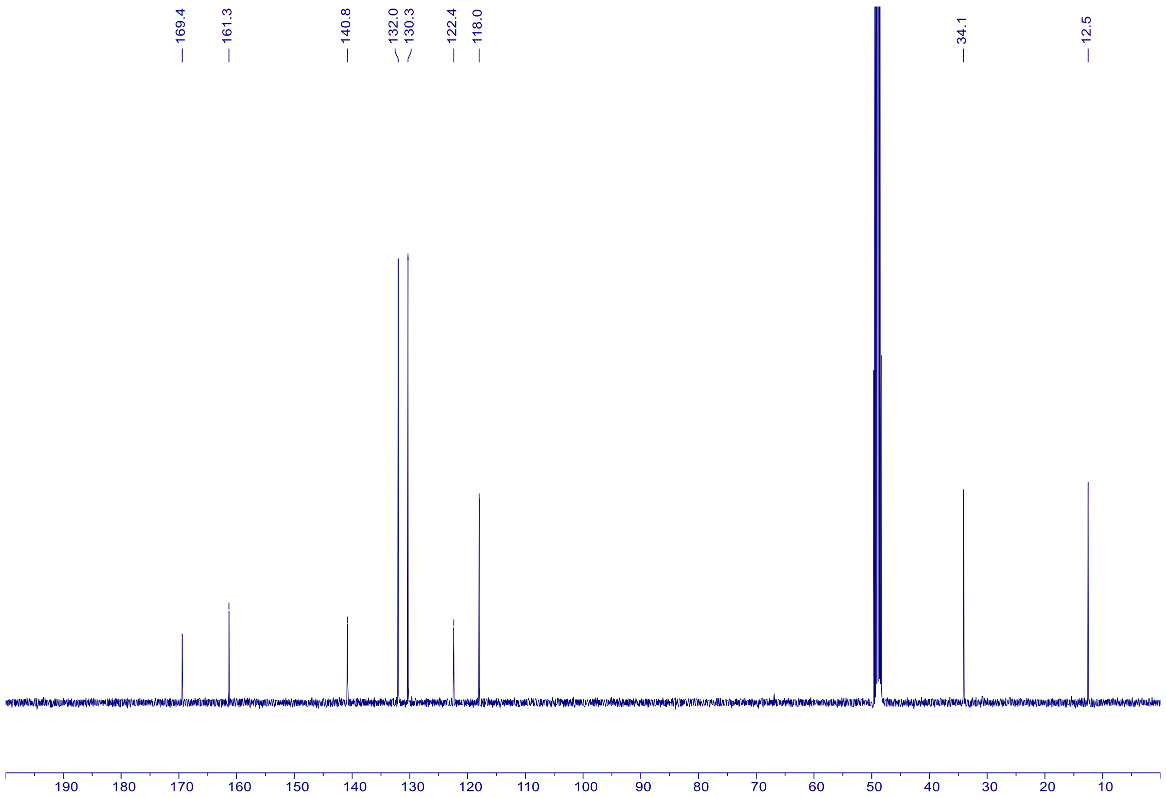

***Z*-23** – ^{1}H NMR (600 MHz, MeOD-d_4)

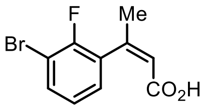

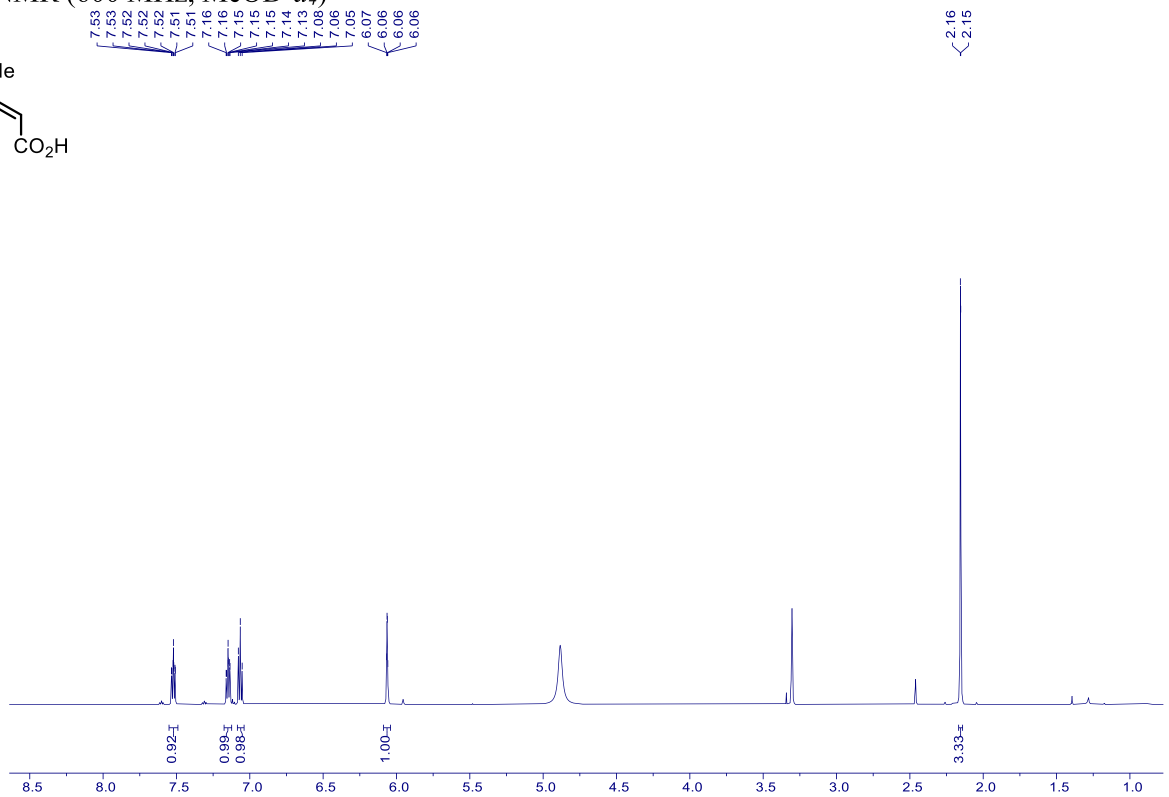

***Z*-23** – ^{13}C NMR (151 MHz, MeOD-d_4)

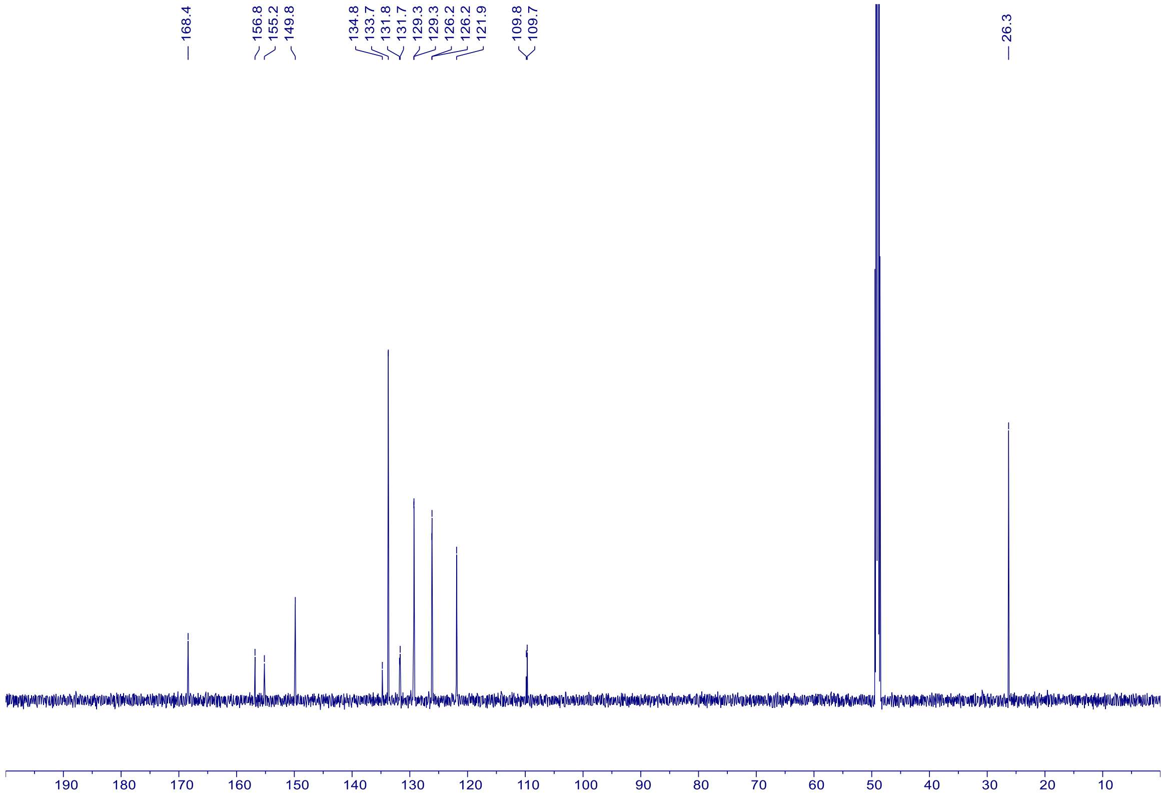

***Z*-23** – ^{19}F NMR (564 MHz, MeOD-d_4)

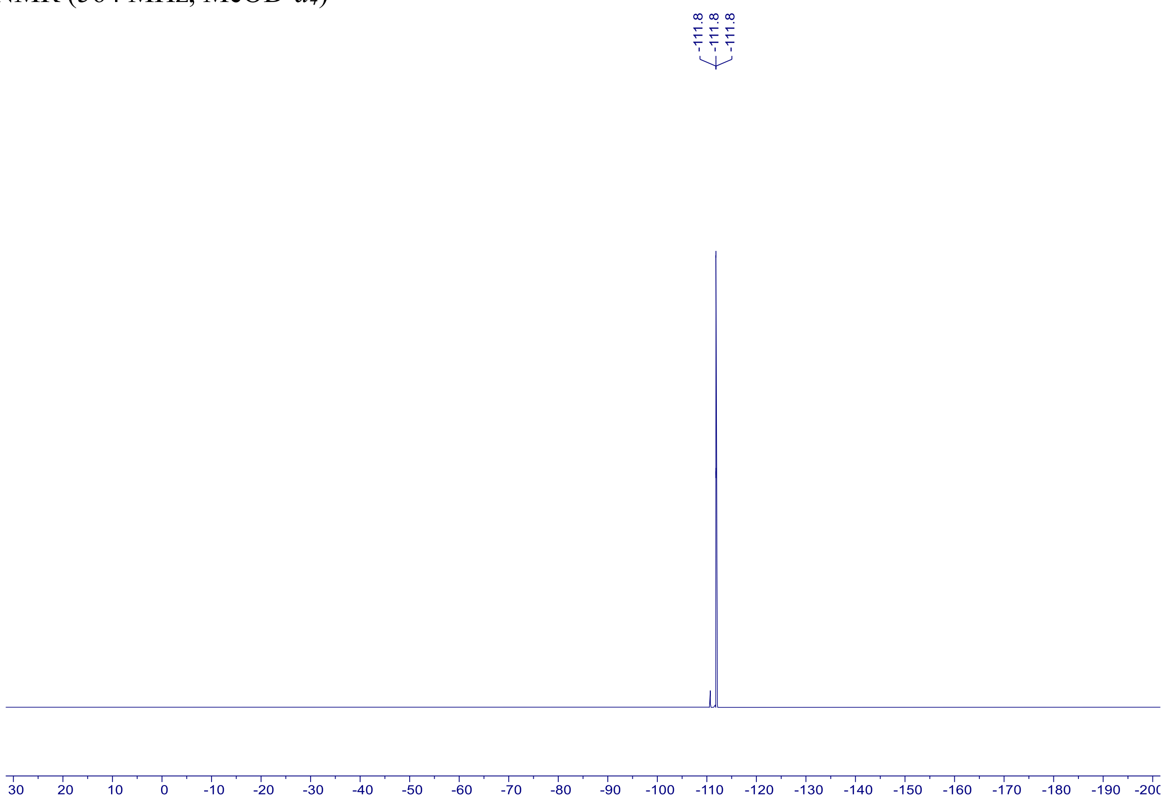

trans-**36** – ^{1}H NMR (600 MHz, $CDCl_3$)

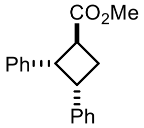

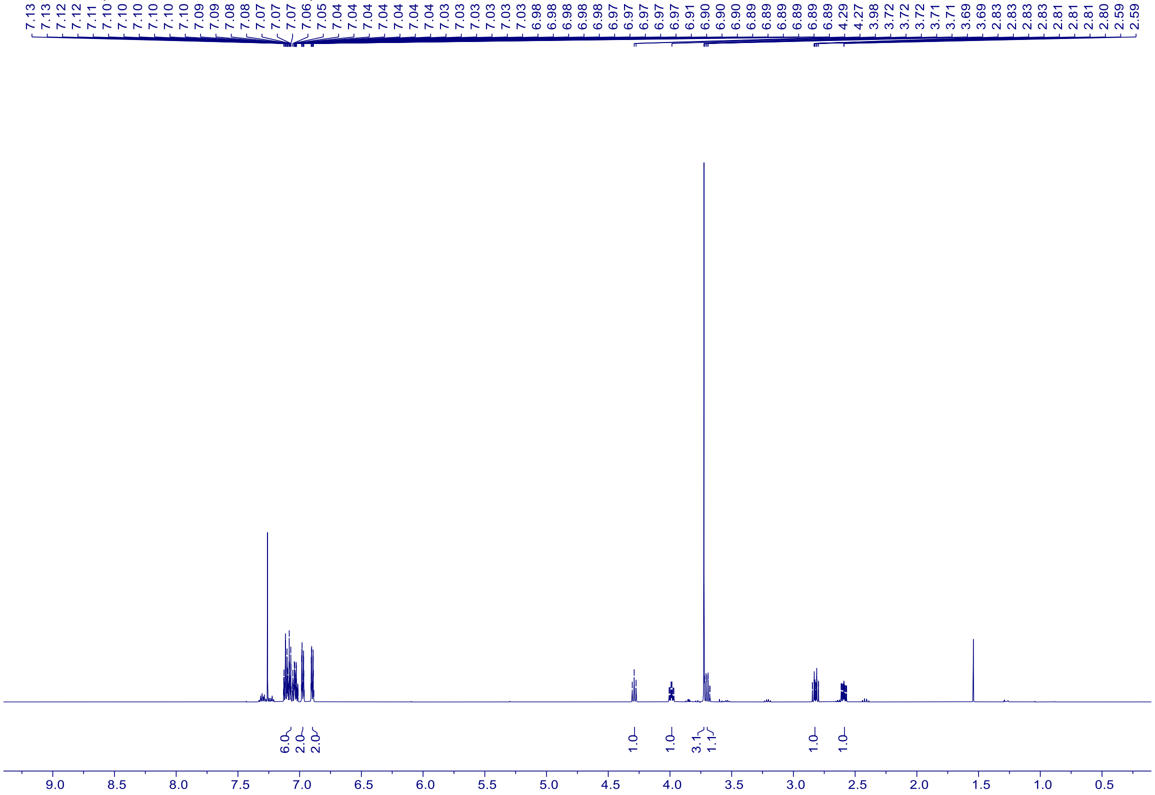

trans-**36** – ^{13}C NMR (151 MHz, $CDCl_3$)

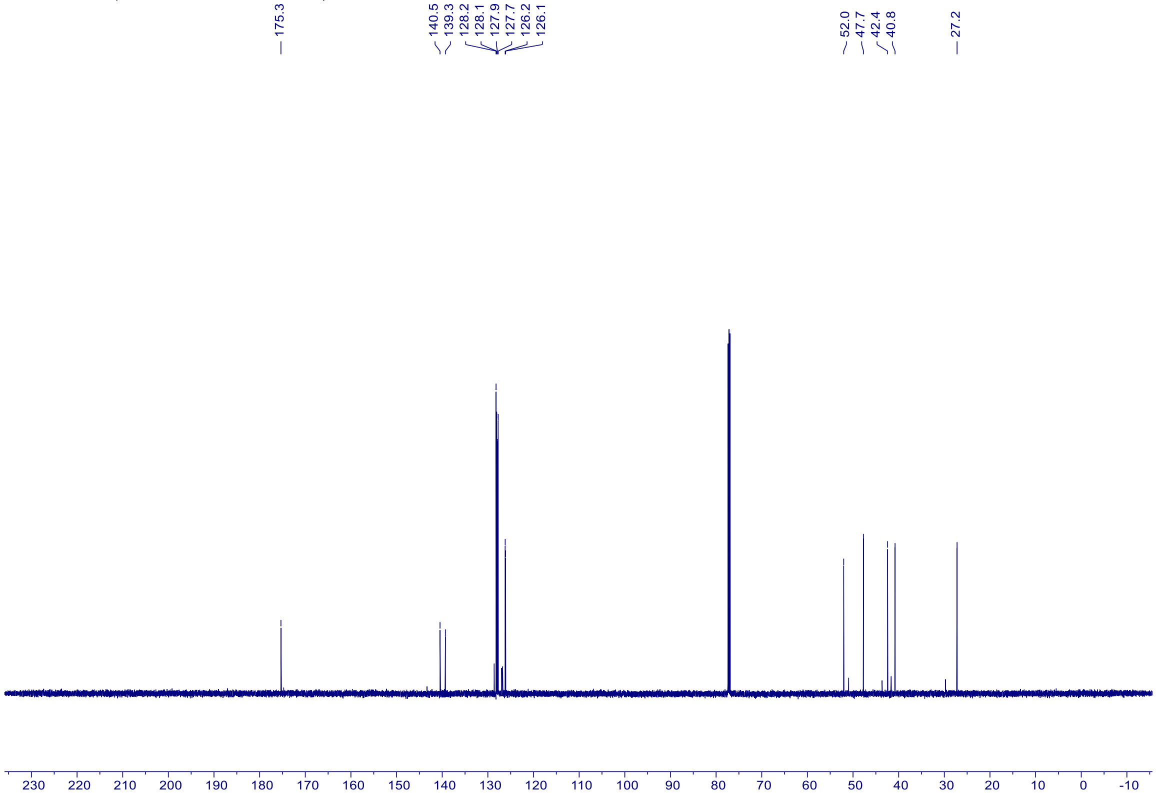

Supplementary References

1. Frisch, M. J. et al., Gaussian 16, Revision C.01, Gaussian, Inc., Wallingford CT (2016).
2. Zhao, Y. & Truhlar, D. G. The M06 Suite of Density Functionals for Main Group Thermochemistry, Thermochemical Kinetics, Noncovalent Interactions, Excited States, and Transition Elements: Two New Functionals and Systematic Testing of Four M06-Class Functionals and 12 Other Functionals. *Theor. Chem. Acc.*, **120**, 215–241 (2008).
3. Dunning Jr., T. H. Gaussian basis sets for use in correlated molecular calculations. I. The atoms boron through neon and hydrogen. *J. Chem. Phys.* **90**, 1007–1023 (1989).
4. Marenich, A. V., Cramer, C. J. & Truhlar, D. G. Universal Solvation Model Based on Solute Electron Density and on a Continuum Model of the Solvent Defined by the Bulk Dielectric Constant and Atomic Surface Tensions. *J. Phys. Chem. B* **113**, 6378–6396 (2009).
5. Schleyer, P. v. R., Maerker, C., Dransfeid, A., Jiao, H. & Hommes, N. J. R. v. E. Nucleus-Independent Chemical Shifts: A Simple and Efficient Aromaticity Probe. *J. Am. Chem. Soc.* **118**, 6317–6318 (1996).
6. Fors, B. P. & Buchwald, S. L. Pd-Catalyzed Conversion of Aryl Chlorides, Triflates, and Nonaflates to Nitroaromatics. *J. Am. Chem. Soc*. **131**, 12898–12899 (2009).
7. Zahid, M. *et al* Synthesis and Photophysical Properties of 2,6-Dicyano-p-phenylenediamine. *Journal of Photochemistry and Photobiology A: Chemistry* **220**, 54–63 (2011).
8. Metternich, J. B. & Gilmour, R. A Bio-Inspired, Catalytic *E→Z* Isomerization of Activated Olefins. *J. Am. Chem. Soc.* **137**, 11254–11257 (2015).
9. Murakami, R., Kojima, N., Kamo, R., Tanishima, H. & Inagaki, F. Photoisomerization of Alkenes via Energy Transfer Enabled by Cu-Acetylide Complexes. *Eur. J. Org. Chem.*, **26**, e202300948 (2023).
10. Zhang, Y. *et al* Energy-Transfer-Mediated Photocatalysis by a Bioinspired Organic Perylenephotosensitizer HiBRCP. *J. Org. Chem.* **86**, 15284–15297 (2021).
11. Molloy, J. J., Metternich, J. B., Daniliuc, C. G., Watson, A. J. B. & Gilmour, R. Contra-Thermodynamic, Photocatalytic *E→Z* Isomerization of Styrenyl Boron Species: Vectors to Facilitate Exploration of Two-Dimensional Chemical Space. *Angew. Chem. Int. Ed.* **57**, 3168–3172 (2018).
12. Wang, C., Wu, C. & Ge, S. Iron-Catalyzed *E*-Selective Dehydrogenative Borylation of Vinylarenes with Pinacolborane. *ACS Catal.* **6**, 7585–7589 (2016).
13. Bitai, J., Nimmo, A. J., Slawin, A. M. Z. & Smith, A. D. Cooperative Palladium/Isothiourea Catalyzed Enantioselective Formal (3+2) Cycloaddition of Vinylcyclopropanes and α,β-Unsaturated Esters. *Angew. Chem. Int. Ed.* **61**, e202202621 (2022).
14. Arai, N., Sato, K., Azuma, K. & Ohkuma, T. Enantioselective Isomerization of Primary Allylic Alcohols into Chiral Aldehydes with the tol-binap/dbapen/Ruthenium(II) Catalyst. *Angew. Chem. Int. Ed.* **52**, 7500–7504 (2013).
15. Cong., X. *et al* Regio- and Diastereoselective Annulation of α,β-Unsaturated Aldimines with Alkenes via Allylic C(sp^3)–H Activation by Rare-Earth Catalysts. *J. Am. Chem. Soc.* **146**, 10187–10198 (2024).

16. Metternich, J. B. *et al* Photocatalytic *E→Z* Isomerization of Polarized Alkenes Inspired by the Visual Cycle: Mechanistic Dichotomy and Origin of Selectivity. *J. Org. Chem.* **82**, 9955−9977 (2017).
17. Pontini, L., Leitch, J. A., and Browne, D. L. Mechanochemical Simmons–Smith Cyclopropanation via Ball-Milling-Enabled Activation of Zinc(0). *Green Chem.* **25**, 4319-4325 (2023).
18. Xu, P.; Wang, F.; Fan, G.; Xu, X. & Tang, P. Hypervalent Iodine (III)-Mediated Oxidative Fluorination of Alkylsilanes by Fluoride Ions. *Angew. Chem. Int. Ed.* **56**, 1101–1104 (2017).
19. Lu, Z. & Yoon, T. P. Visible Light Photocatalysis of [2+2] Styrene Cycloadditions by Energy Transfer. *Angew. Chem. Int. Ed.* **51**, 10329–10332 (2012).
20. Mojr, V. *et al* Tailoring Flavins for Visible Light Photocatalysis: Organocatalytic [2+2] Cycloadditions Mediated by a Flavin Derivative and Visible Light. *Chem. Commun.* **51**, 12036–12039 (2015).
21. Oppolzer, W. & Stammen, B. On the Faciality of Intramolecular Palladium(0)-Catalysed "metallo-Ene-Type" Cyclisations. *Tetrahedron* **53**, 3577–3586 (1997).
22. Lee., Y. R. & Wang, X. First Concise Synthesis of Biologically Interesting Nigrolineabenzopyran A, (±)-Blandachromene II, and (±)-Daurichromene D. *Bull. Korean Chem. Soc.* **28**, 2061–2064 (2007).
23. Becker, M. R., Richardson, A. D. & Schinder, C. S. Functionalized Azetidines & Visible Light-Enabled aza Paternò-Büchi Reactions. *Nat. Comm.* **10**, 5095 (2019). DOI: 10.1038/s41467-019-13072-x.
24. Zheng, Y., Dong, Q.-X., Wen, S.-Y., Ran, H. & Huang, H.-M. Di-π-ethane Rearrangement of Cyano Groups via Energy-Transfer Catalysis. J. Am. Chem. Soc. 146, 27, 18210–18217 (2024).
25. Lima, C. G. S., De M. Lima, T., Duarte, M., Jurberg, I. D. & Paixão, M. W. Organic Synthesis Enabled by Light-Irradiation of EDA Complexes: Theoretical Background and Synthetic Applications. *ACS Catal.* **6**, 1389–1407 (2016).
26. Solé-Daura, A. & Maseras, F. Straightforward Computational Determination of Energy-Transfer Kinetics Through the Application of the Marcus Theory. *Chem. Sci.* **15**, 13650–13658 (2024).
27. Weng, X., Huang, J., Liu, Y. & Chen, S. The Decisive Role of Electrostatic Interactions in Transport Mode and Phase Segregation of Lithium Ions in $LiFePO_4$. *Chem. Sci.* **14**, 13042–13049 (2023).
28. Tian, W. F. *et al Cis*-Selective Transfer Semihydrogenation of Alkynes by Merging Visible-Light Catalysis with Cobalt Catalysis. *Adv. Synth. Catal.* **362**, 1032–1038 (2020).
29. Beveridge, R. E. & Arndtsen, B. A. A Direct, Copper-Catalyzed Functionalization of Pyridines with Alkynes. *Synthesis* **6**, 1000–1008 (2010).
30. Metternich, J. B. & Gilmour, R. One Photocatalyst, *n* Activation Modes Strategy for Cascade Catalysis: Emulating Coumarin Biosynthesis with (−)-Riboflavin. *J. Am. Chem. Soc.* **138**, 1040–1045 (2016).
31. Yang, X. & Wang, C. Dichotomy of Manganese Catalysis via Organometallic or RadicalMechanism: Stereodivergent Hydrosilylation of Alkynes. *Angew. Chem. Int. Ed.* **57**, 923–928 (2018).
32. Fan, S. *et al* Organic Luminescent Cocrystals Based on BenzotriazoleDerivatives: Synthesis, Characterization, Crystal Structureand Fluorescence Behavior. *Chem. Eur. J.* **31**, e202403889 (2025).

33. Scholz, S. O. *et al* Construction of Complex Cyclobutane Building Blocks by Photosensitized [2 + 2] Cycloaddition of Vinyl Boronate Esters. *Org. Lett.* **23**, 3496–3501 (2021).
34. Mojr, V. *et al* Flavin Photocatalysts for Visible-Light [2+2] Cycloadditions: Structure, Reactivity and Reaction Mechanism. *ChemCatChem* **10**, 849–858 (2018).
35. Daub, M. E. *et al* Enantioselective [2+2] Cycloadditions of Cinnamate Esters: Generalizing Lewis Acid Catalysis of Triplet Energy Transfer. *J. Am. Chem. Soc.* **141**, 9543–9547 (2019).
36. Liu, Y. *et al* Photosensitized [2+2]-Cycloadditions of Alkenylboronates and Alkenes. Angew. *Chem. Int. Ed.* **61**, e202200725 (2022).
37. Lei, T. *et al* General and Efficient Intermolecular [2+ 2] Photodimerization of Chalcones and Cinnamic Acid Derivatives in Solution Through Visible-Light Catalysis. *Angew. Chem. Int. Ed.* **56**, 15407–15410 (2017).
